# Supplementary material for: ProminTools: shedding light on proteins of unknown function in biomineralization with user friendly tools illustrated using mollusc shell matrix protein sequences
Source: PeerJ. 2020 Sep 11;8:e9852. doi: 10.7717/peerj.9852 (PMC7489238; doi:10.7717/peerj.9852)
Supplement: Supplemental Information 3 — Full ProminTools output with input files [file peerj-08-9852-s003.zip › Mgal/Mgallo_6_motifs.html]

Overrepresented motifs in your proteins of interest


# Overrepresented motifs in your proteins of interest

#### A tool by Alastair Skeffington hosted by Cyverse and making use of the motif-x motif finding engine

#### 2020-07-06

The main outputs of the program are listed below:

- Mgallo\_6 \_bgmotifs.txt : counts of each of the overrepresented motifs in each of the proteins in the background sequence set
- Mgallo\_6 \_fgmotifs.txt : counts of each of the overrepresented motifs in each of the proteins in the foreground sequence set
- Mgallo\_6 \_fgenrich.txt : the enrichment of each of the overrepresented motifs in each of the proteins in the foreground sequence set with repspect to the background sequence set
- Mgallo\_6 \_motifsummary.txt : for each overrepresented motif, the enrichemnt in the forgraound sequnces relative to the background; the count of proteins in which it appears and the median count of the motif per protein
- Mgallo\_6 \_motifs.html : A html summary of the analysis with some helpful plots
- Mgallo\_6 \_Rscript.R : The R script that generated the html output. This can be used as a basis to produce your own custom figures and plots in R
- Mgallo\_6 \_wordclouds.svg : An svg file of the wordclouds from the html report. This can be imported into Inkscape or other software to make publication ready figures
- Mgallo\_6 \_positions.txt: This contains the sequence composistion biases detected by fLPS

The main outputs of the program are the data tables. For convenience these are processed to a html document with some visualizations of the data. However one analysis pipeline can never be appropriate for every dataset, so the R script used to generate these diagrams is also an output of the program. This is to allow you, or an infromatically minded colleague to easily tweak the plots or to develop these analyses further in a way appropriate for your data.   
 The table below shows the motifs that are significantly overrepresented in your protein set of interest (PSOI) compared to the background proteome. In your dataset 89 overrepresented motifs were identified. If you removed the PSOI from the background proteome fasta file which you used as an input, the you may have motifs that are infinitely enriched (‘Inf’) because they are present in the PSOI but not in the background.

| Motif | Enrichment | Count of proteins with motif | Median motif count per protein |
| --- | --- | --- | --- |
| D..D.VKD | Inf | 1 | 0.0 |
| DSEEF | Inf | 1 | 0.0 |
| GYDKK | Inf | 1 | 0.0 |
| GYSNY | Inf | 2 | 0.0 |
| IG..R.Y.C | Inf | 1 | 0.0 |
| KGY.NY | Inf | 2 | 0.0 |
| P.F.C..C | Inf | 1 | 0.0 |
| PG..G.MG | Inf | 1 | 0.0 |
| Q..PPP..Q | Inf | 1 | 0.0 |
| QL..FD..N | Inf | 1 | 0.0 |
| RTTT…L | Inf | 1 | 0.0 |
| RTTT.A | Inf | 1 | 0.0 |
| VR.Y.C | Inf | 3 | 0.0 |
| YD.KGY | Inf | 2 | 0.0 |
| GP.G.M | 562.35 | 1 | 0.0 |
| YD.KG | 192.81 | 4 | 0.0 |
| G.M.P.G | 180.76 | 2 | 0.0 |
| GP..PP | 96.40 | 3 | 0.0 |
| GPPG..G | 87.03 | 1 | 0.0 |
| DG..L.G | 65.73 | 3 | 0.0 |
| C..GY…G | 59.19 | 1 | 0.0 |
| GL.G..G | 28.29 | 7 | 0.0 |
| GLGG | 24.23 | 9 | 0.0 |
| AAAA | 23.17 | 3 | 0.0 |
| P.Q.Q | 19.88 | 11 | 0.0 |
| YD.K | 18.87 | 6 | 0.0 |
| PQQ | 17.04 | 9 | 0.0 |
| P…PG | 15.49 | 12 | 0.0 |
| G…F…G | 12.11 | 12 | 0.0 |
| G.A..G | 9.93 | 14 | 0.0 |
| G..G..G | 9.53 | 18 | 0.0 |
| G.GG | 9.12 | 22 | 1.0 |
| G…LG | 9.04 | 15 | 0.0 |
| G.Q…G | 8.63 | 13 | 0.0 |
| G…G…G | 7.61 | 18 | 0.0 |
| TT..P | 6.98 | 10 | 0.0 |
| D…D..D | 6.97 | 9 | 0.0 |
| SDS | 6.91 | 8 | 0.0 |
| G..GS | 6.83 | 18 | 0.0 |
| GG..G | 5.06 | 19 | 0.5 |
| TTT | 5.04 | 8 | 0.0 |
| G…IG | 5.01 | 8 | 0.0 |
| Q.Q | 4.37 | 25 | 1.0 |
| GL.G | 4.17 | 15 | 0.0 |
| G.G.G | 4.10 | 18 | 0.0 |
| P…P | 4.10 | 26 | 1.0 |
| PQ | 4.08 | 23 | 1.0 |
| P..P | 3.94 | 25 | 1.0 |
| GP | 3.79 | 29 | 1.0 |
| QP | 3.53 | 21 | 1.0 |
| G..FG | 3.07 | 14 | 0.0 |
| C.P | 3.05 | 12 | 0.0 |
| G.G | 3.04 | 35 | 4.0 |
| GQ | 2.90 | 30 | 2.0 |
| G..G | 2.87 | 37 | 4.0 |
| N.G | 2.79 | 33 | 2.5 |
| G..R | 2.75 | 29 | 2.0 |
| P…M | 2.59 | 20 | 1.0 |
| G.N | 2.57 | 31 | 2.0 |
| G.A | 2.45 | 33 | 2.0 |
| AP | 2.40 | 24 | 1.0 |
| P.T | 2.33 | 25 | 1.0 |
| GY | 2.29 | 18 | 0.0 |
| GAG | 2.27 | 13 | 0.0 |
| P..R | 2.25 | 20 | 1.0 |
| G…R | 2.18 | 32 | 2.0 |
| D.S | 2.13 | 25 | 1.0 |
| G..D | 2.12 | 29 | 2.0 |
| T.T | 2.10 | 24 | 1.0 |
| N..G | 2.09 | 30 | 2.0 |
| V.D | 2.09 | 27 | 1.0 |
| DS | 2.06 | 26 | 1.0 |
| G.S | 2.01 | 34 | 3.5 |
| VP | 1.97 | 24 | 1.0 |
| EP | 1.87 | 18 | 0.0 |
| SG | 1.76 | 34 | 4.0 |
| I.P | 1.62 | 19 | 0.5 |
| D…G | 1.61 | 33 | 2.0 |
| A..S | 1.54 | 32 | 2.0 |
| G…I | 1.51 | 28 | 2.0 |
| PS | 1.50 | 26 | 1.0 |
| G.F | 1.30 | 28 | 2.0 |
| L.P | 1.21 | 26 | 1.0 |
| G…T | 1.20 | 31 | 2.0 |
| VG | 1.07 | 25 | 2.0 |
| W.W | 1.02 | 4 | 0.0 |
| W..P | 1.01 | 9 | 0.0 |
| G.V | 1.00 | 31 | 2.0 |
| G…M | 0.92 | 18 | 0.0 |

The data in the table above can be visualized as wordclouds. To avoid plotting problems, any infinitely enriched motifs were first removed from the data. In this analyses we are not interested in the peculiarities of particular proteins, but in the properties of this group of proteins as a whole, so any motifs found in less than 5% of the proteins or in only one protein, were also removed before plotting.

In the wordcoulds the height of the letters relates to either the number of proteins in the set of interest containing a given motif (left), the enrichment of a motif relative to the background proteome (middle), or the product of the scaled values of these two measures (right). In some datasets with very few motifs, the scaled values are numerically undefined and no wordcloud will be plotted.

In general there tends to be a negative correlation between the enrichment of a motif and the number of proteins in which it is found. It is possible that motifs that deviate from this trend (ie they are unusually enriched given the number of proteins in which they are found, or are in an unusually large number of proteins given their enrichment) might have particular biological significance. The scatter plots below plot the enrichment of the motifs against the number of proteins in which they are found (and vice versa) to help you visualize any deviations from the expected negative correlation. If a linear regression is appropriate, a regression line (either linear or polynomial) is shown in red and the shaded area represents the 95% confidence interval of the regression. No regression line is shown in cases with strong non-linearity. Points are labelled only if there is sufficient space in the plot.

You may be interested in grouping or subdividing your PSOI based on the motif content of the proteins. The heatmaps below provide a starting point for such approaches. In the first heatmap, proteins are clustered based on motif enrichment in a given protein sequence with respect the the background sequences, and motifs are clustered based on their enrichment amongst proteins. Clustering is restricted to proteins displaying a high degree of compositional bias (depending on the value of the fLPS p-value parameter chosen by the user). This is because a relationship between cluster membership and shared function has only been demonstrated for this class of proteins. Infinitely enriched motifs, if present in the data, are excluded. Clustering is further restricted to the 70 motifs with the greatest overall enrichment in this set, and a motif must be found in at least 3 proteins to enter the clustering processes. This prevents the heatmap being dominated by motifs that are extremely enriched, but only found in one or two proteins. This filter is not applied if the number of proteins is small (<10). Inevitably some proteins in the resulting data set have little similarity to other proteins. To ensure we only group proteins that have true similarity, proteins are required to have a distance correlation of at least 0.65 with one other protein in the data set to be inlcuded in the set for clustering. Proteins containing none of the motifs are not displayed.

In the second heatmap, proteins are clustered based on motif enrichment exactly as in heatmap 1, except that the filter requiring that motifs are found in at least 4 proteins is not applied. These heatmaps are therefore more often dominated by unusally enriched motifs found in only one or two proteins.

In the third heatmap, proteins are clustered based on the absolute number of motifs. This heatmap is intended as context for the previous two. It displays the same set of proteins and motifs as in heatmap 2.
