# Supplementary material for: ProminTools: shedding light on proteins of unknown function in biomineralization with user friendly tools illustrated using mollusc shell matrix protein sequences
Source: PeerJ. 2020 Sep 11;8:e9852. doi: 10.7717/peerj.9852 (PMC7489238; doi:10.7717/peerj.9852)
Supplement: Supplemental Information 7 — Details of the procedure and descriptions of files can be found in the R markdown document. [file peerj-08-9852-s007.zip › Supp7/top500_motifs.html]

Overrepresented motifs in your proteins of interest


# Overrepresented motifs in your proteins of interest

#### A tool by Alastair Skeffington hosted by Cyverse and making use of the motif-x motif finding engine

#### 2020-05-15

The main outputs of the program are listed below:

- top500 \_bgmotifs.txt : counts of each of the overrepresented motifs in each of the proteins in the background sequence set
- top500 \_fgmotifs.txt : counts of each of the overrepresented motifs in each of the proteins in the foreground sequence set
- top500 \_fgenrich.txt : the enrichment of each of the overrepresented motifs in each of the proteins in the foreground sequence set with repspect to the background sequence set
- top500 \_motifsummary.txt : for each overrepresented motif, the enrichemnt in the forgraound sequnces relative to the background; the count of proteins in which it appears and the median count of the motif per protein
- top500 \_motifs.html : A html summary of the analysis with some helpful plots
- top500 \_Rscript.R : The R script that generated the html output. This can be used as a basis to produce your own custom figures and plots in R
- top500 \_wordclouds.svg : An svg file of the wordclouds from the html report. This can be imported into Inkscape or other software to make publication ready figures

The main outputs of the program are the data tables. For convenience these are processed to a html document with some visualizations of the data. However one analysis pipeline can never be appropriate for every dataset, so the R script used to generate these diagrams is also an output of the program. This is to allow you, or an infromatically minded colleague to easily tweak the plots or to develop these analyses further in a way appropriate for your data.

| Motif | Enrichment | Count of proteins with motif | Median motif count per protein |
| --- | --- | --- | --- |
| A..SR.IIS | Inf | 5 | 0.0 |
| A.EVVPEK | Inf | 1 | 0.0 |
| A.PLPVTSF | Inf | 21 | 0.0 |
| A.STG.AT | Inf | 82 | 0.0 |
| A.STG.TTP | Inf | 79 | 0.0 |
| A.T.DTT.L | Inf | 82 | 0.0 |
| A.T.RTRTS | Inf | 79 | 0.0 |
| A.YRKD..A | Inf | 1 | 0.0 |
| A.YRKDL | Inf | 1 | 0.0 |
| AA.IQSD | Inf | 5 | 0.0 |
| AA.THT..A | Inf | 79 | 0.0 |
| AAKAAK.GV | Inf | 11 | 0.0 |
| AAKIQSDR | Inf | 5 | 0.0 |
| AALVTF.PE | Inf | 68 | 0.0 |
| AAPSSH..T | Inf | 79 | 0.0 |
| ADAGDL..V | Inf | 17 | 0.0 |
| ADTP.M | Inf | 5 | 0.0 |
| ADTPLM | Inf | 5 | 0.0 |
| AEPTE..QR | Inf | 2 | 0.0 |
| AEPTEH.E | Inf | 2 | 0.0 |
| AEPTENG | Inf | 2 | 0.0 |
| AF..LEQQH | Inf | 1 | 0.0 |
| AG..G.SRT | Inf | 79 | 0.0 |
| AGAGDL..V | Inf | 46 | 0.0 |
| AGT.W.W.L | Inf | 40 | 0.0 |
| AIPLPVTS | Inf | 78 | 0.0 |
| AITTTT.VT | Inf | 4 | 0.0 |
| AKAAK.GV | Inf | 11 | 0.0 |
| AKKCQVL | Inf | 5 | 0.0 |
| AL.TFD.E | Inf | 68 | 0.0 |
| ALHDTDAS | Inf | 61 | 0.0 |
| ALPVT.TSS | Inf | 69 | 0.0 |
| ALQ.AP.SH | Inf | 79 | 0.0 |
| ALQ.S.K.Y | Inf | 5 | 0.0 |
| AMTHT…E | Inf | 79 | 0.0 |
| AN.KTT | Inf | 2 | 0.0 |
| AN.TLR.S | Inf | 25 | 0.0 |
| ANE.TT.S | Inf | 2 | 0.0 |
| ANEKTT | Inf | 2 | 0.0 |
| ANENTT | Inf | 2 | 0.0 |
| ANP.H..SP | Inf | 68 | 0.0 |
| AP.PTGDG | Inf | 79 | 0.0 |
| APP.E.TTS | Inf | 79 | 0.0 |
| APT.DGH | Inf | 79 | 0.0 |
| APTTAL..A | Inf | 79 | 0.0 |
| AQTQRTR | Inf | 79 | 0.0 |
| AS..HATHL | Inf | 82 | 0.0 |
| AS.GDTST | Inf | 79 | 0.0 |
| AS.GHAT.V | Inf | 79 | 0.0 |
| ASI.HAT.L | Inf | 68 | 0.0 |
| ASPLLVT | Inf | 79 | 0.0 |
| ASQ.GTLW | Inf | 40 | 0.0 |
| ASS.STDHA | Inf | 68 | 0.0 |
| ASS.STGHA | Inf | 82 | 0.0 |
| ASSASTGH | Inf | 82 | 0.0 |
| ASSGH.T.L | Inf | 82 | 0.0 |
| ASSLSTRH | Inf | 81 | 0.0 |
| ASSVFT..A | Inf | 69 | 0.0 |
| ASSVPT.H | Inf | 79 | 0.0 |
| AST.DSTP | Inf | 79 | 0.0 |
| AST.HATL | Inf | 68 | 0.0 |
| ASTGDTT | Inf | 82 | 0.0 |
| ASTGHTT.L | Inf | 82 | 0.0 |
| ASTGQ..PL | Inf | 82 | 0.0 |
| ASTRHAT | Inf | 58 | 0.0 |
| AT.LPVT.L | Inf | 82 | 0.0 |
| AT.LPVTDT | Inf | 82 | 0.0 |
| ATALH.T | Inf | 82 | 0.0 |
| ATP.PVTNT | Inf | 79 | 0.0 |
| ATPLPLT | Inf | 79 | 0.0 |
| ATSL.VT | Inf | 82 | 0.0 |
| ATSLLVT | Inf | 82 | 0.0 |
| ATSLPVT | Inf | 82 | 0.0 |
| ATSPSSSP | Inf | 79 | 0.0 |
| AVSQRGH.Q | Inf | 79 | 0.0 |
| AWE.VK.T | Inf | 5 | 0.0 |
| AY.LQSDS | Inf | 5 | 0.0 |
| AYD.QSDS | Inf | 5 | 0.0 |
| C.ADV…V | Inf | 19 | 0.0 |
| CC.YGP.G | Inf | 25 | 0.0 |
| CFP.Q.D.I | Inf | 5 | 0.0 |
| CL.WLKS | Inf | 25 | 0.0 |
| CSYGP.G | Inf | 25 | 0.0 |
| D…DAIGI | Inf | 5 | 0.0 |
| D..TT.APT | Inf | 79 | 0.0 |
| D..YKT.YE | Inf | 5 | 0.0 |
| D.EGQS.VT | Inf | 11 | 0.0 |
| D.K.KGYD | Inf | 5 | 0.0 |
| D.KYK..YC | Inf | 5 | 0.0 |
| D.PLK.E.P | Inf | 58 | 0.0 |
| D.SSASTG | Inf | 82 | 0.0 |
| D.SSISTG | Inf | 72 | 0.0 |
| D.SSVST.H | Inf | 82 | 0.0 |
| D.SSVSTG | Inf | 82 | 0.0 |
| D.TPLP.T | Inf | 82 | 0.0 |
| D.TPVPVT | Inf | 79 | 0.0 |
| DAI.I.HAK | Inf | 5 | 0.0 |
| DASSLS.G | Inf | 81 | 0.0 |
| DASSV.TG | Inf | 82 | 0.0 |
| DDPKSV | Inf | 5 | 0.0 |
| DH.AKI..P | Inf | 5 | 0.0 |
| DI..DAI.I | Inf | 5 | 0.0 |
| DI.SD.LYK | Inf | 5 | 0.0 |
| DIISD.KY | Inf | 5 | 0.0 |
| DIR.DAI | Inf | 5 | 0.0 |
| DLEWLKG | Inf | 5 | 0.0 |
| DLEWLKG.G | Inf | 5 | 0.0 |
| DLGAP.G | Inf | 1 | 0.0 |
| DMMSI…K | Inf | 5 | 0.0 |
| DREYKKA | Inf | 5 | 0.0 |
| DS.YK.D.E | Inf | 5 | 0.0 |
| DST.LP.T | Inf | 79 | 0.0 |
| DT..LPVT | Inf | 82 | 0.0 |
| DT.ASS.ST | Inf | 61 | 0.0 |
| DT.SAST | Inf | 82 | 0.0 |
| DTDTSS.ST | Inf | 68 | 0.0 |
| DTP.MLQ | Inf | 5 | 0.0 |
| DTPEVI.A | Inf | 5 | 0.0 |
| DTSS.STG | Inf | 82 | 0.0 |
| DTSSA.T | Inf | 82 | 0.0 |
| DTSSAS.G | Inf | 82 | 0.0 |
| DTSSEST | Inf | 79 | 0.0 |
| DTSSSSTG | Inf | 79 | 0.0 |
| DTTPL.VT | Inf | 82 | 0.0 |
| DTTR.PV | Inf | 82 | 0.0 |
| DV.YR.Y.H | Inf | 5 | 0.0 |
| E..QMR..T | Inf | 19 | 0.0 |
| E.EKANV | Inf | 5 | 0.0 |
| E.IQAK.NA | Inf | 5 | 0.0 |
| E.QRVKH.Q | Inf | 5 | 0.0 |
| E.T.NE.TT | Inf | 2 | 0.0 |
| E.TANE.T | Inf | 2 | 0.0 |
| E.TTPSPA | Inf | 2 | 0.0 |
| E.VLAKA | Inf | 5 | 0.0 |
| E.VLAKAN | Inf | 5 | 0.0 |
| EAL.FTSI | Inf | 5 | 0.0 |
| ECL.WLK | Inf | 25 | 0.0 |
| EDANFT | Inf | 25 | 0.0 |
| EDPKLV | Inf | 5 | 0.0 |
| EETSLS..G | Inf | 79 | 0.0 |
| EG.ST.ASS | Inf | 78 | 0.0 |
| EH.ERTA.E | Inf | 2 | 0.0 |
| EK.KG.HN | Inf | 5 | 0.0 |
| EM.RVK.AQ | Inf | 5 | 0.0 |
| EPTENG..T | Inf | 2 | 0.0 |
| EQQHVGL | Inf | 1 | 0.0 |
| ER..HNQE | Inf | 7 | 0.0 |
| ER.KLNQE | Inf | 5 | 0.0 |
| ERLYR..PE | Inf | 5 | 0.0 |
| ESQCLY | Inf | 23 | 0.0 |
| ESQTT.S.S | Inf | 79 | 0.0 |
| ETAPPD..T | Inf | 79 | 0.0 |
| ETHTLS.S | Inf | 68 | 0.0 |
| ETP.MTTPS | Inf | 58 | 0.0 |
| ETSLSV.G | Inf | 79 | 0.0 |
| ETSSASTG | Inf | 21 | 0.0 |
| ETTTSSPSS | Inf | 68 | 0.0 |
| EWLKG.G | Inf | 5 | 0.0 |
| EYK.AYEK | Inf | 5 | 0.0 |
| F…PTDVP | Inf | 7 | 0.0 |
| F.MPTS..S | Inf | 68 | 0.0 |
| F.P.PTGDG | Inf | 79 | 0.0 |
| F.S.PSR.S | Inf | 79 | 0.0 |
| F.VSTG.TT | Inf | 21 | 0.0 |
| FDPEG.SP | Inf | 68 | 0.0 |
| FIR.TR.S | Inf | 25 | 0.0 |
| FPSGET.TS | Inf | 68 | 0.0 |
| FPYGA..G | Inf | 63 | 0.0 |
| FPYGAD.G | Inf | 17 | 0.0 |
| FS..VSQE | Inf | 79 | 0.0 |
| FSPTPT | Inf | 16 | 0.0 |
| FT.RD.CT | Inf | 25 | 0.0 |
| FY.LEQQH | Inf | 1 | 0.0 |
| G..EM.RVK | Inf | 5 | 0.0 |
| G..TPLPVT | Inf | 87 | 0.0 |
| G..TSLPVT | Inf | 82 | 0.0 |
| G..TSPPH | Inf | 16 | 0.0 |
| G.A.PLPVT | Inf | 82 | 0.0 |
| G.AGTI..V | Inf | 79 | 0.0 |
| G.ATL.PVT | Inf | 69 | 0.0 |
| G.D.RADAI | Inf | 5 | 0.0 |
| G.EGQS..T | Inf | 79 | 0.0 |
| G.G.MPE.S | Inf | 5 | 0.0 |
| G.GIPIP | Inf | 5 | 0.0 |
| G.PQFPP | Inf | 14 | 0.0 |
| G.PVLMG | Inf | 25 | 0.0 |
| G.TQAP.TT | Inf | 79 | 0.0 |
| G.TSP.TET | Inf | 68 | 0.0 |
| GAGAGDL | Inf | 46 | 0.0 |
| GD..PLLVT | Inf | 79 | 0.0 |
| GD.HTT..P | Inf | 79 | 0.0 |
| GD.MPLP.T | Inf | 79 | 0.0 |
| GDTSTLP.T | Inf | 79 | 0.0 |
| GDTTP.P | Inf | 82 | 0.0 |
| GDTTP.P.T | Inf | 82 | 0.0 |
| GDTTPL..T | Inf | 82 | 0.0 |
| GET.T.SPS | Inf | 68 | 0.0 |
| GF.TL..DP | Inf | 5 | 0.0 |
| GFG.VPG | Inf | 13 | 0.0 |
| GGPHFI | Inf | 40 | 0.0 |
| GH..PL.VT | Inf | 82 | 0.0 |
| GH.TP.PVT | Inf | 82 | 0.0 |
| GH.TPL.VT | Inf | 82 | 0.0 |
| GH.TS.PV | Inf | 82 | 0.0 |
| GHA.HL.V | Inf | 82 | 0.0 |
| GHATAL..T | Inf | 82 | 0.0 |
| GHHMG.R | Inf | 5 | 0.0 |
| GHTTS…S | Inf | 21 | 0.0 |
| GI.WLPEG | Inf | 5 | 0.0 |
| GIGW..EG | Inf | 5 | 0.0 |
| GIGW.PE | Inf | 5 | 0.0 |
| GKPEGP.S | Inf | 5 | 0.0 |
| GNPSTGT | Inf | 68 | 0.0 |
| GPH.I..TR | Inf | 25 | 0.0 |
| GPT.NRE.T | Inf | 2 | 0.0 |
| GQ.TA.PVT | Inf | 69 | 0.0 |
| GQS.A.SSR | Inf | 78 | 0.0 |
| GT..P.TPE | Inf | 5 | 0.0 |
| GTDTSS.ST | Inf | 82 | 0.0 |
| GTLWP…R | Inf | 40 | 0.0 |
| GTM.TS..V | Inf | 40 | 0.0 |
| GTPLPVTS | Inf | 58 | 0.0 |
| GTTFYQS | Inf | 14 | 0.0 |
| GTVSQK.S | Inf | 79 | 0.0 |
| GVS.E.FTL | Inf | 79 | 0.0 |
| H.TPL.VT | Inf | 82 | 0.0 |
| H.TSLHV | Inf | 81 | 0.0 |
| H.WSC.PD | Inf | 5 | 0.0 |
| HA.PL.VT | Inf | 82 | 0.0 |
| HAK.SR.IA | Inf | 5 | 0.0 |
| HAKA.R.I | Inf | 5 | 0.0 |
| HASPL…D | Inf | 79 | 0.0 |
| HAT.L..TD | Inf | 82 | 0.0 |
| HAT.LPVT | Inf | 82 | 0.0 |
| HATPLP.T | Inf | 82 | 0.0 |
| HATPVPVT | Inf | 79 | 0.0 |
| HDT.ASS.S | Inf | 61 | 0.0 |
| HKPGPP | Inf | 10 | 0.0 |
| HMGFR…D | Inf | 5 | 0.0 |
| HMGFR.L | Inf | 5 | 0.0 |
| HNVTGT.S | Inf | 79 | 0.0 |
| HP..KPGPP | Inf | 10 | 0.0 |
| HQAES.E.S | Inf | 79 | 0.0 |
| HT..A.STE | Inf | 79 | 0.0 |
| HT.SPSGS | Inf | 79 | 0.0 |
| HTQ.P.TT | Inf | 79 | 0.0 |
| HVGLAVDM | Inf | 1 | 0.0 |
| HVT.PS.AS | Inf | 82 | 0.0 |
| I..DTPEM | Inf | 5 | 0.0 |
| I..SVSTGD | Inf | 58 | 0.0 |
| I.HAKASR | Inf | 5 | 0.0 |
| I.STG.T.P | Inf | 68 | 0.0 |
| IASDYLY | Inf | 5 | 0.0 |
| IG..TPLPV | Inf | 10 | 0.0 |
| IGW.PE.S | Inf | 5 | 0.0 |
| IHAAK…D | Inf | 5 | 0.0 |
| IKHAK.S | Inf | 5 | 0.0 |
| IKPEA…T | Inf | 5 | 0.0 |
| IKT.T.PSS | Inf | 79 | 0.0 |
| INA.QIS | Inf | 5 | 0.0 |
| IP.GS.E.M | Inf | 25 | 0.0 |
| IPILPE..E | Inf | 16 | 0.0 |
| IPIP.TPE | Inf | 5 | 0.0 |
| IPIPI.PE | Inf | 5 | 0.0 |
| IPRVPS..S | Inf | 79 | 0.0 |
| IPS.SS.GH | Inf | 79 | 0.0 |
| IQHAK…D | Inf | 5 | 0.0 |
| IR.DAI.I | Inf | 5 | 0.0 |
| IR.TRRS | Inf | 25 | 0.0 |
| IS.AK.GQ | Inf | 5 | 0.0 |
| IS.KLY.E | Inf | 5 | 0.0 |
| IS.PVDM | Inf | 5 | 0.0 |
| ISN.L..KD | Inf | 5 | 0.0 |
| IST.TTV.P | Inf | 4 | 0.0 |
| ITAGQ.G.S | Inf | 79 | 0.0 |
| ITGSK.P.P | Inf | 10 | 0.0 |
| ITTTTMVT | Inf | 2 | 0.0 |
| ITTTTTMTP | Inf | 3 | 0.0 |
| IVDTPE.I | Inf | 5 | 0.0 |
| K…PGMTT | Inf | 58 | 0.0 |
| K..FRDGL | Inf | 1 | 0.0 |
| K.GVG.P.A | Inf | 6 | 0.0 |
| K.N.LQIS | Inf | 5 | 0.0 |
| K.PTDVP | Inf | 7 | 0.0 |
| K.QVLVSD | Inf | 5 | 0.0 |
| KE.P.PPE | Inf | 14 | 0.0 |
| KG.YIG..S | Inf | 5 | 0.0 |
| KGY.IRA | Inf | 5 | 0.0 |
| KIS.PVDM | Inf | 5 | 0.0 |
| KISI..DM | Inf | 5 | 0.0 |
| KP..LKFT | Inf | 5 | 0.0 |
| KPE.P..TV | Inf | 1 | 0.0 |
| KPGPPQ.P | Inf | 10 | 0.0 |
| KQ.GH.MG | Inf | 5 | 0.0 |
| KQ.GHYIG | Inf | 5 | 0.0 |
| KT.TSPSSS | Inf | 79 | 0.0 |
| KT.YE..KG | Inf | 5 | 0.0 |
| KTPAP.TST | Inf | 10 | 0.0 |
| KV..MPTS | Inf | 68 | 0.0 |
| KVA.D..NE | Inf | 5 | 0.0 |
| KVSAI.E | Inf | 79 | 0.0 |
| KYG.VPG | Inf | 8 | 0.0 |
| L..DAIGI | Inf | 5 | 0.0 |
| L..QQQQHQ | Inf | 14 | 0.0 |
| L..TDTSSV | Inf | 82 | 0.0 |
| L..TET.SA | Inf | 21 | 0.0 |
| L..TGHTT | Inf | 11 | 0.0 |
| L.DDPK..W | Inf | 5 | 0.0 |
| L.NERLYR | Inf | 5 | 0.0 |
| L.P.EP.EN | Inf | 2 | 0.0 |
| L.PVTS..L | Inf | 79 | 0.0 |
| L.SGTM.TS | Inf | 40 | 0.0 |
| L.T.TSSVS | Inf | 79 | 0.0 |
| L.V.ETSSV | Inf | 61 | 0.0 |
| L.VTDASS | Inf | 82 | 0.0 |
| L.VTDT.SA | Inf | 82 | 0.0 |
| L.VTG.SS | Inf | 82 | 0.0 |
| L.VTIPS | Inf | 79 | 0.0 |
| L.VTN..SV | Inf | 79 | 0.0 |
| LEG.ST.AS | Inf | 78 | 0.0 |
| LFPYG.G.G | Inf | 46 | 0.0 |
| LH.WSC.P | Inf | 5 | 0.0 |
| LHD.DA.SL | Inf | 61 | 0.0 |
| LHVT..SS | Inf | 82 | 0.0 |
| LHVTS.SSA | Inf | 82 | 0.0 |
| LHVTSPS | Inf | 82 | 0.0 |
| LKM.TPG.T | Inf | 58 | 0.0 |
| LLVTD.SS | Inf | 82 | 0.0 |
| LNQEN.S.V | Inf | 5 | 0.0 |
| LP.TDTS | Inf | 82 | 0.0 |
| LP.TPEM.R | Inf | 5 | 0.0 |
| LP.TY.S.A | Inf | 79 | 0.0 |
| LPLTS.SSV | Inf | 79 | 0.0 |
| LPTGFT | Inf | 39 | 0.0 |
| LPVTD.SS | Inf | 82 | 0.0 |
| LPVTDTSS | Inf | 82 | 0.0 |
| LPVTG.SS | Inf | 82 | 0.0 |
| LPVTN.SS | Inf | 82 | 0.0 |
| LPVTS..SV | Inf | 82 | 0.0 |
| LPVTSFSSA | Inf | 68 | 0.0 |
| LPVTSLSS | Inf | 82 | 0.0 |
| LPVTSPSS | Inf | 82 | 0.0 |
| LPVTSPSSA | Inf | 82 | 0.0 |
| LPVTSRSSA | Inf | 58 | 0.0 |
| LPVTSSSSA | Inf | 61 | 0.0 |
| LPVTSTSSS | Inf | 10 | 0.0 |
| LPVTSTSSV | Inf | 79 | 0.0 |
| LQ..INA.Q | Inf | 5 | 0.0 |
| LQA.PSS.D | Inf | 79 | 0.0 |
| LQWLK..P | Inf | 25 | 0.0 |
| LS..TS.YV | Inf | 19 | 0.0 |
| LS.VLYKE | Inf | 5 | 0.0 |
| LSSATT..T | Inf | 79 | 0.0 |
| LSSPS.S.T | Inf | 79 | 0.0 |
| LSSPSGF.P | Inf | 79 | 0.0 |
| LST.HTT.L | Inf | 21 | 0.0 |
| LSTGHAT | Inf | 79 | 0.0 |
| LSVSG..SA | Inf | 79 | 0.0 |
| LTSPL..GP | Inf | 40 | 0.0 |
| LTSPQTET | Inf | 68 | 0.0 |
| LVGGPHF | Inf | 40 | 0.0 |
| LVT.N.STG | Inf | 79 | 0.0 |
| LVT.TSSAS | Inf | 82 | 0.0 |
| LVTFDPEG | Inf | 68 | 0.0 |
| LW..WLR.G | Inf | 40 | 0.0 |
| LY..AWEK | Inf | 5 | 0.0 |
| LYKENV..G | Inf | 5 | 0.0 |
| LYQ.AW.K | Inf | 5 | 0.0 |
| M..PTGT | Inf | 19 | 0.0 |
| M..TATSPS | Inf | 79 | 0.0 |
| M.PTPT | Inf | 3 | 0.0 |
| M.TPG.T.P | Inf | 58 | 0.0 |
| M.TSRDS | Inf | 68 | 0.0 |
| MAQTQ.TRT | Inf | 79 | 0.0 |
| METAPPD | Inf | 69 | 0.0 |
| MLT.PLV.G | Inf | 40 | 0.0 |
| MM..V.AKK | Inf | 5 | 0.0 |
| MP.FST..V | Inf | 14 | 0.0 |
| MPVTS…V | Inf | 79 | 0.0 |
| MTH.H.A.S | Inf | 79 | 0.0 |
| N…P.DTP | Inf | 5 | 0.0 |
| N..TTPSP | Inf | 2 | 0.0 |
| N.LQIS.K | Inf | 5 | 0.0 |
| N.SSASTGH | Inf | 82 | 0.0 |
| N.SSILYK | Inf | 5 | 0.0 |
| N.T.R.SCT | Inf | 25 | 0.0 |
| NA..IS.PL | Inf | 5 | 0.0 |
| NASSLST | Inf | 82 | 0.0 |
| NE.LYR..P | Inf | 5 | 0.0 |
| NE.TTPS.A | Inf | 2 | 0.0 |
| NLSS.LYK | Inf | 5 | 0.0 |
| NLSSV.YK | Inf | 5 | 0.0 |
| NMG.G.P.P | Inf | 5 | 0.0 |
| NNGGYK | Inf | 25 | 0.0 |
| NP..DS.TT | Inf | 79 | 0.0 |
| NPSTG..GT | Inf | 79 | 0.0 |
| NQ.NLS.V | Inf | 5 | 0.0 |
| NRE.TAN.K | Inf | 2 | 0.0 |
| NTP.DM.S | Inf | 5 | 0.0 |
| NTR.SM..T | Inf | 25 | 0.0 |
| NTSSASTG | Inf | 11 | 0.0 |
| NTTPSP..P | Inf | 2 | 0.0 |
| NVT.TVS | Inf | 79 | 0.0 |
| P..PEVP.V | Inf | 1 | 0.0 |
| P..TQSTT | Inf | 2 | 0.0 |
| P.CNC..G | Inf | 13 | 0.0 |
| P.DTP.ML | Inf | 5 | 0.0 |
| P.KEPE.P | Inf | 11 | 0.0 |
| P.PTGDG | Inf | 79 | 0.0 |
| P.SQGG.K | Inf | 5 | 0.0 |
| P.TDTSS.S | Inf | 82 | 0.0 |
| P.TSRSS.S | Inf | 58 | 0.0 |
| P.YSVT..I | Inf | 79 | 0.0 |
| PAEPTE.G | Inf | 2 | 0.0 |
| PAPVTST.S | Inf | 10 | 0.0 |
| PE..QAK.N | Inf | 5 | 0.0 |
| PE..RVKL | Inf | 5 | 0.0 |
| PE.QRVKH | Inf | 5 | 0.0 |
| PEGQSPVT | Inf | 11 | 0.0 |
| PEGS.EV | Inf | 5 | 0.0 |
| PEI.LA.M | Inf | 5 | 0.0 |
| PETSPY | Inf | 19 | 0.0 |
| PG.SQ.I.T | Inf | 79 | 0.0 |
| PG.VGN.G | Inf | 19 | 0.0 |
| PGKPEGP.S | Inf | 5 | 0.0 |
| PGMTTPSL | Inf | 58 | 0.0 |
| PGSPE.M | Inf | 25 | 0.0 |
| PHF.RNT | Inf | 25 | 0.0 |
| PILPER.E | Inf | 16 | 0.0 |
| PISTTTTVT | Inf | 4 | 0.0 |
| PK..WA..V | Inf | 5 | 0.0 |
| PK..WA.H | Inf | 5 | 0.0 |
| PK.VVL.K | Inf | 1 | 0.0 |
| PL.VTD.SS | Inf | 82 | 0.0 |
| PLHVTS.SS | Inf | 79 | 0.0 |
| PLKMETP | Inf | 58 | 0.0 |
| PLLVT..SS | Inf | 82 | 0.0 |
| PLLVT.T | Inf | 82 | 0.0 |
| PLLVTD.S | Inf | 82 | 0.0 |
| PLP.T.EME | Inf | 5 | 0.0 |
| PLP.TDT.S | Inf | 82 | 0.0 |
| PLPLTS.SS | Inf | 79 | 0.0 |
| PLPTGFT | Inf | 39 | 0.0 |
| PLPVT.LSS | Inf | 82 | 0.0 |
| PLPVT.PSS | Inf | 82 | 0.0 |
| PLPVT.SSS | Inf | 61 | 0.0 |
| PLPVTD.SS | Inf | 82 | 0.0 |
| PLPVTE.SS | Inf | 61 | 0.0 |
| PLPVTN.SS | Inf | 82 | 0.0 |
| PLPVTSFSS | Inf | 79 | 0.0 |
| PLPVTSPS | Inf | 82 | 0.0 |
| PLPVTSPSS | Inf | 82 | 0.0 |
| PLPVTY.SS | Inf | 79 | 0.0 |
| PLTSLS.V | Inf | 79 | 0.0 |
| PNP.PT..T | Inf | 39 | 0.0 |
| PP..MTTSF | Inf | 79 | 0.0 |
| PP.TVPE.P | Inf | 1 | 0.0 |
| PPGSP..M | Inf | 25 | 0.0 |
| PPSITST | Inf | 68 | 0.0 |
| PQTET.T.S | Inf | 68 | 0.0 |
| PR.KPGPP | Inf | 10 | 0.0 |
| PRVPS.VS | Inf | 79 | 0.0 |
| PS..STG.T | Inf | 82 | 0.0 |
| PS.ETTTSS | Inf | 68 | 0.0 |
| PSASPQ.T | Inf | 79 | 0.0 |
| PSKVSA | Inf | 79 | 0.0 |
| PSR.SHTT | Inf | 79 | 0.0 |
| PSS.SSG.T | Inf | 79 | 0.0 |
| PSSAS.GH | Inf | 82 | 0.0 |
| PSSASTGH | Inf | 82 | 0.0 |
| PSTGTA.T | Inf | 68 | 0.0 |
| PT.AL.AA | Inf | 79 | 0.0 |
| PT.IT.T.T | Inf | 4 | 0.0 |
| PTE.G..TP | Inf | 2 | 0.0 |
| PTEH.E.T | Inf | 2 | 0.0 |
| PTG.QD..A | Inf | 19 | 0.0 |
| PTG.TGR | Inf | 39 | 0.0 |
| PTGDG..TQ | Inf | 79 | 0.0 |
| PTGTQ.PTP | Inf | 4 | 0.0 |
| PTLTP.TT | Inf | 2 | 0.0 |
| PTP.HSQ | Inf | 16 | 0.0 |
| PTP.S.TST | Inf | 70 | 0.0 |
| PTPT.ITT | Inf | 4 | 0.0 |
| PTPTGTQAP | Inf | 2 | 0.0 |
| PTPTGTQSP | Inf | 4 | 0.0 |
| PTPTPTSTQ | Inf | 2 | 0.0 |
| PTS.DSTLG | Inf | 68 | 0.0 |
| PTS.P.ST | Inf | 2 | 0.0 |
| PTSPS.SPQ | Inf | 79 | 0.0 |
| PV.MG.S.G | Inf | 25 | 0.0 |
| PV.SFSS.S | Inf | 79 | 0.0 |
| PVDMVS | Inf | 5 | 0.0 |
| PVPVT..SS | Inf | 79 | 0.0 |
| PVT.ASSAS | Inf | 82 | 0.0 |
| PVT.LS.A | Inf | 82 | 0.0 |
| PVT.PSS.S | Inf | 82 | 0.0 |
| PVT.TSSVS | Inf | 82 | 0.0 |
| PVT.VSS.S | Inf | 82 | 0.0 |
| PVTDASS | Inf | 82 | 0.0 |
| PVTDIS..S | Inf | 79 | 0.0 |
| PVTDNS | Inf | 21 | 0.0 |
| PVTDTS.AS | Inf | 82 | 0.0 |
| PVTDTSS | Inf | 82 | 0.0 |
| PVTDTSS.S | Inf | 82 | 0.0 |
| PVTGLSS.S | Inf | 82 | 0.0 |
| PVTIPS..S | Inf | 79 | 0.0 |
| PVTNASS.S | Inf | 82 | 0.0 |
| PVTNTSSAS | Inf | 11 | 0.0 |
| PVTSAA..T | Inf | 79 | 0.0 |
| PVTSLSS.S | Inf | 82 | 0.0 |
| PVTSTSLAS | Inf | 11 | 0.0 |
| PWW.RS.T | Inf | 40 | 0.0 |
| PY.AD..DL | Inf | 17 | 0.0 |
| PYG.GAG | Inf | 46 | 0.0 |
| Q…SRTTS | Inf | 79 | 0.0 |
| Q.E.HTLSP | Inf | 68 | 0.0 |
| Q.FPS.P.G | Inf | 16 | 0.0 |
| Q.GHHMG | Inf | 5 | 0.0 |
| Q.NLSS.LY | Inf | 5 | 0.0 |
| Q.PTSTP.T | Inf | 4 | 0.0 |
| Q.TPLPVT | Inf | 82 | 0.0 |
| Q.TRFS.T | Inf | 16 | 0.0 |
| Q.TRTSR.S | Inf | 79 | 0.0 |
| QA.PLPVT | Inf | 82 | 0.0 |
| QAAPSSHD | Inf | 79 | 0.0 |
| QAESTEAS | Inf | 79 | 0.0 |
| QAGTLWP | Inf | 40 | 0.0 |
| QAK.CQ.LV | Inf | 5 | 0.0 |
| QAP.TT..S | Inf | 79 | 0.0 |
| QAT.L.VTS | Inf | 82 | 0.0 |
| QATPL.VT | Inf | 82 | 0.0 |
| QDDPK..W | Inf | 5 | 0.0 |
| QIS.K.YQ | Inf | 5 | 0.0 |
| QKGHYI | Inf | 5 | 0.0 |
| QKGYD…D | Inf | 5 | 0.0 |
| QP.WM…P | Inf | 25 | 0.0 |
| QQQ.QWQ | Inf | 14 | 0.0 |
| QS.REYK.A | Inf | 5 | 0.0 |
| QSDS.YK | Inf | 5 | 0.0 |
| QSPVT.SRT | Inf | 11 | 0.0 |
| QT..TRTS | Inf | 79 | 0.0 |
| QT.TPTPI | Inf | 4 | 0.0 |
| QT.TSTP.S | Inf | 2 | 0.0 |
| QTTRF…P | Inf | 16 | 0.0 |
| QTTRS..P | Inf | 79 | 0.0 |
| QV.VSD..Y | Inf | 5 | 0.0 |
| QW..AQ.SV | Inf | 14 | 0.0 |
| R…DDPKS | Inf | 5 | 0.0 |
| R…GPM.C | Inf | 14 | 0.0 |
| R..TATS.K | Inf | 14 | 0.0 |
| R.DTSTL | Inf | 58 | 0.0 |
| R.ECL.W | Inf | 25 | 0.0 |
| R.K.NQ.NL | Inf | 5 | 0.0 |
| R.KLNQ.N | Inf | 5 | 0.0 |
| R.PTS..ST | Inf | 68 | 0.0 |
| RAT.I..DN | Inf | 5 | 0.0 |
| RDDVT..S | Inf | 19 | 0.0 |
| REYKKA.E | Inf | 5 | 0.0 |
| RGH.QAP.T | Inf | 79 | 0.0 |
| RGTTFY.S | Inf | 14 | 0.0 |
| RH.LH.W.C | Inf | 5 | 0.0 |
| RH.PGPP | Inf | 10 | 0.0 |
| RKQLG..MG | Inf | 5 | 0.0 |
| RLPVT..SS | Inf | 82 | 0.0 |
| RRSMV…C | Inf | 25 | 0.0 |
| RSGTM.TS | Inf | 40 | 0.0 |
| RSSASTGH | Inf | 58 | 0.0 |
| RSVSP.T.T | Inf | 79 | 0.0 |
| RTRTSR.S | Inf | 79 | 0.0 |
| RTSRGSDT | Inf | 79 | 0.0 |
| RVPSKV.A | Inf | 79 | 0.0 |
| RY.QAWE | Inf | 5 | 0.0 |
| S…QTTRF | Inf | 16 | 0.0 |
| S..PGVSQE | Inf | 79 | 0.0 |
| S..TGHATS | Inf | 82 | 0.0 |
| S.ASR.D.S | Inf | 58 | 0.0 |
| S.GDT.PL | Inf | 82 | 0.0 |
| S.GDTT.LP | Inf | 82 | 0.0 |
| S.GHATS.P | Inf | 82 | 0.0 |
| S.ILYKE | Inf | 5 | 0.0 |
| S.KLYQ..W | Inf | 5 | 0.0 |
| S.MTTPS | Inf | 21 | 0.0 |
| S.P.DMVS | Inf | 5 | 0.0 |
| S.QIS..LY | Inf | 5 | 0.0 |
| S.SGF.PSG | Inf | 79 | 0.0 |
| S.SPMT.T | Inf | 79 | 0.0 |
| S.SSASTGD | Inf | 82 | 0.0 |
| S.SSVSTG | Inf | 82 | 0.0 |
| S.STG.TTP | Inf | 82 | 0.0 |
| S.STGDTM | Inf | 79 | 0.0 |
| S.STGQAT | Inf | 82 | 0.0 |
| S.T.I.DTP | Inf | 5 | 0.0 |
| S.T.TPITT | Inf | 4 | 0.0 |
| S.TPAP.TS | Inf | 10 | 0.0 |
| S.TSPSASP | Inf | 79 | 0.0 |
| S.V.AKKCQ | Inf | 5 | 0.0 |
| S.YKAD.E | Inf | 5 | 0.0 |
| SA.ST.DTT | Inf | 21 | 0.0 |
| SAALV.F.P | Inf | 68 | 0.0 |
| SAAST.H.T | Inf | 82 | 0.0 |
| SAS.GD.ST | Inf | 79 | 0.0 |
| SAS.GDTT | Inf | 82 | 0.0 |
| SAS.GH.TS | Inf | 82 | 0.0 |
| SASPQ.T.A | Inf | 79 | 0.0 |
| SASTD.TT | Inf | 79 | 0.0 |
| SASTGD.T | Inf | 82 | 0.0 |
| SASTGD.TP | Inf | 82 | 0.0 |
| SASTGH.TH | Inf | 82 | 0.0 |
| SASTGH.TL | Inf | 68 | 0.0 |
| SASTGH.TS | Inf | 82 | 0.0 |
| SASTGHVT | Inf | 79 | 0.0 |
| SASTGQ.TP | Inf | 82 | 0.0 |
| SASTGQAT | Inf | 82 | 0.0 |
| SASTR.A | Inf | 58 | 0.0 |
| SATTD.TT | Inf | 79 | 0.0 |
| SAYTG..TS | Inf | 79 | 0.0 |
| SC.PDQN | Inf | 5 | 0.0 |
| SD..YKT.Y | Inf | 5 | 0.0 |
| SDS.YK.D | Inf | 5 | 0.0 |
| SDSVY..D | Inf | 5 | 0.0 |
| SDV.YR.Y | Inf | 5 | 0.0 |
| SDYLYK | Inf | 5 | 0.0 |
| SEP.YR | Inf | 5 | 0.0 |
| SEPLY..A | Inf | 5 | 0.0 |
| SFTS..DT | Inf | 5 | 0.0 |
| SGETTT.S | Inf | 68 | 0.0 |
| SGHTT..P | Inf | 82 | 0.0 |
| SGTISA.T | Inf | 79 | 0.0 |
| SGTMLT.P | Inf | 40 | 0.0 |
| SI..AK.GQ | Inf | 5 | 0.0 |
| SIGHATPL | Inf | 10 | 0.0 |
| SITAG.EG | Inf | 79 | 0.0 |
| SITST.LTS | Inf | 68 | 0.0 |
| SK.TPLPVT | Inf | 5 | 0.0 |
| SKV.A.G.P | Inf | 79 | 0.0 |
| SKV.R.PTS | Inf | 68 | 0.0 |
| SL.PY.AD | Inf | 17 | 0.0 |
| SLFPYG.G | Inf | 46 | 0.0 |
| SLHVTS.SS | Inf | 81 | 0.0 |
| SLPVSDT | Inf | 21 | 0.0 |
| SLPVT..SS | Inf | 82 | 0.0 |
| SLPVTIPS | Inf | 79 | 0.0 |
| SLSTR..TS | Inf | 81 | 0.0 |
| SLSVSGT | Inf | 79 | 0.0 |
| SN.TIP..T | Inf | 5 | 0.0 |
| SNPSRDS | Inf | 79 | 0.0 |
| SP.TG..PM | Inf | 14 | 0.0 |
| SP.VGG.H | Inf | 40 | 0.0 |
| SPGVSQE | Inf | 79 | 0.0 |
| SPLKME.P | Inf | 58 | 0.0 |
| SPMG..PG | Inf | 16 | 0.0 |
| SPQTET..L | Inf | 68 | 0.0 |
| SPSASPQ | Inf | 79 | 0.0 |
| SPSSAS.GD | Inf | 79 | 0.0 |
| SPSSS..GQ | Inf | 21 | 0.0 |
| SPSSSPML | Inf | 79 | 0.0 |
| SPSST.TG | Inf | 61 | 0.0 |
| SPSYSVT | Inf | 79 | 0.0 |
| SPVTFSRT | Inf | 11 | 0.0 |
| SQ.TRS..P | Inf | 79 | 0.0 |
| SQAGT.W | Inf | 40 | 0.0 |
| SQRGH.QA | Inf | 79 | 0.0 |
| SQT.AR.S | Inf | 16 | 0.0 |
| SR..HT.QS | Inf | 79 | 0.0 |
| SRDIASD.L | Inf | 5 | 0.0 |
| SRG.TST.P | Inf | 58 | 0.0 |
| SS..T.DTT | Inf | 82 | 0.0 |
| SS.RDSLYA | Inf | 24 | 0.0 |
| SS.SKG.TT | Inf | 78 | 0.0 |
| SS.SSG.TT | Inf | 82 | 0.0 |
| SS.STG.TT | Inf | 82 | 0.0 |
| SS.STGDT | Inf | 82 | 0.0 |
| SS.STGHT | Inf | 82 | 0.0 |
| SS.STRH.T | Inf | 81 | 0.0 |
| SSA.ST.DT | Inf | 21 | 0.0 |
| SSA.TDD | Inf | 79 | 0.0 |
| SSAS.GDT | Inf | 82 | 0.0 |
| SSASI..AT | Inf | 68 | 0.0 |
| SSASSGH | Inf | 82 | 0.0 |
| SSAST.DT | Inf | 82 | 0.0 |
| SSASTD.T | Inf | 79 | 0.0 |
| SSASTG.AI | Inf | 82 | 0.0 |
| SSASTGD | Inf | 82 | 0.0 |
| SSASTGD.T | Inf | 82 | 0.0 |
| SSASTGH.N | Inf | 68 | 0.0 |
| SSASTGH.P | Inf | 10 | 0.0 |
| SSASTGH.S | Inf | 79 | 0.0 |
| SSASTGHV | Inf | 79 | 0.0 |
| SSASTGQ.T | Inf | 82 | 0.0 |
| SSASTGQA | Inf | 82 | 0.0 |
| SSESTG.VT | Inf | 79 | 0.0 |
| SSFSP.VSQ | Inf | 79 | 0.0 |
| SSG.TT.LP | Inf | 82 | 0.0 |
| SSILYKE | Inf | 5 | 0.0 |
| SSIST.HAT | Inf | 72 | 0.0 |
| SSLSTGHA | Inf | 79 | 0.0 |
| SSNPS.DS | Inf | 79 | 0.0 |
| SSPSG..PS | Inf | 79 | 0.0 |
| SSPSYS.T | Inf | 79 | 0.0 |
| SSPTS..AS | Inf | 79 | 0.0 |
| SSS.GPWT | Inf | 16 | 0.0 |
| SSS.H.T.L | Inf | 79 | 0.0 |
| SSSTG.AT | Inf | 21 | 0.0 |
| SSVST.HAT | Inf | 82 | 0.0 |
| SSVSTG..T | Inf | 82 | 0.0 |
| SSVSTGH | Inf | 82 | 0.0 |
| ST.HAP | Inf | 10 | 0.0 |
| ST.HAT.LH | Inf | 82 | 0.0 |
| ST.HAT.LP | Inf | 82 | 0.0 |
| ST.SS..GQ | Inf | 10 | 0.0 |
| STAASS.TS | Inf | 78 | 0.0 |
| STG.AGT.P | Inf | 79 | 0.0 |
| STG.AIPL | Inf | 82 | 0.0 |
| STG.AT.LP | Inf | 82 | 0.0 |
| STG.STPL | Inf | 79 | 0.0 |
| STG.TTPL | Inf | 82 | 0.0 |
| STG.TTPV | Inf | 79 | 0.0 |
| STGDTM.LP | Inf | 79 | 0.0 |
| STGDTST | Inf | 79 | 0.0 |
| STGDTT.L | Inf | 82 | 0.0 |
| STGHA..LL | Inf | 82 | 0.0 |
| STGHA.PV | Inf | 79 | 0.0 |
| STGHAN.L | Inf | 68 | 0.0 |
| STGHATSL | Inf | 82 | 0.0 |
| STGHG.P.P | Inf | 58 | 0.0 |
| STGHVTP | Inf | 79 | 0.0 |
| STGHVTPL | Inf | 79 | 0.0 |
| STGLTS | Inf | 68 | 0.0 |
| STGQAT.L | Inf | 82 | 0.0 |
| STGQDT.L | Inf | 79 | 0.0 |
| STLPVT.AS | Inf | 79 | 0.0 |
| STP.S.TTT | Inf | 2 | 0.0 |
| STP.TT.TT | Inf | 4 | 0.0 |
| STR.TTS | Inf | 10 | 0.0 |
| STTLT.I | Inf | 2 | 0.0 |
| STTTTV.P | Inf | 4 | 0.0 |
| STV.SSPS | Inf | 79 | 0.0 |
| SV.NV.ETA | Inf | 68 | 0.0 |
| SVEMN | Inf | 5 | 0.0 |
| SVSGTIS | Inf | 79 | 0.0 |
| SVSTA..TP | Inf | 78 | 0.0 |
| SVSTD..TS | Inf | 68 | 0.0 |
| SVSTG..TP | Inf | 82 | 0.0 |
| SVSTG..TR | Inf | 82 | 0.0 |
| SVSTGHAT | Inf | 82 | 0.0 |
| SVTQM..T | Inf | 79 | 0.0 |
| SY.NPL.T | Inf | 39 | 0.0 |
| SYSVT..IK | Inf | 79 | 0.0 |
| T…KTATS | Inf | 79 | 0.0 |
| T..PISTT | Inf | 4 | 0.0 |
| T..SSVSTG | Inf | 82 | 0.0 |
| T.DTT.LP | Inf | 82 | 0.0 |
| T.F.PTPT | Inf | 16 | 0.0 |
| T.G.EGQS | Inf | 79 | 0.0 |
| T.GM.TP | Inf | 79 | 0.0 |
| T.H.TPLPV | Inf | 82 | 0.0 |
| T.HATSL | Inf | 82 | 0.0 |
| T.HE..TP | Inf | 2 | 0.0 |
| T.I.SDN.Y | Inf | 5 | 0.0 |
| T.IT.T.M | Inf | 2 | 0.0 |
| T.L.VTDAS | Inf | 82 | 0.0 |
| T.LPVT.LS | Inf | 82 | 0.0 |
| T.LPVT.PS | Inf | 82 | 0.0 |
| T.LPVTD.S | Inf | 82 | 0.0 |
| T.LPVTDTS | Inf | 82 | 0.0 |
| T.NE.TTP | Inf | 2 | 0.0 |
| T.P.VPS.V | Inf | 79 | 0.0 |
| T.PLV..PH | Inf | 40 | 0.0 |
| T.PSSASSG | Inf | 79 | 0.0 |
| T.SPA..TE | Inf | 2 | 0.0 |
| T.T.TPIST | Inf | 4 | 0.0 |
| T.TLTP.TT | Inf | 2 | 0.0 |
| T.TPTPITT | Inf | 2 | 0.0 |
| T.TPTPTPI | Inf | 4 | 0.0 |
| T.TPTPTST | Inf | 4 | 0.0 |
| T.TSTPITT | Inf | 4 | 0.0 |
| T.TSTQTPT | Inf | 2 | 0.0 |
| T.YDLR.D | Inf | 5 | 0.0 |
| T.YEK.KG | Inf | 5 | 0.0 |
| TA..IP.VP | Inf | 68 | 0.0 |
| TAASS.TS | Inf | 78 | 0.0 |
| TAITTTTTV | Inf | 4 | 0.0 |
| TAL.A.PSS | Inf | 79 | 0.0 |
| TALHDT..S | Inf | 61 | 0.0 |
| TAPPD..TT | Inf | 79 | 0.0 |
| TD..T.LPV | Inf | 79 | 0.0 |
| TD.SA.ST | Inf | 82 | 0.0 |
| TD.SS..TG | Inf | 82 | 0.0 |
| TD.SS.STG | Inf | 82 | 0.0 |
| TD.SSASTG | Inf | 82 | 0.0 |
| TD.SSVST | Inf | 82 | 0.0 |
| TDT.SAST | Inf | 82 | 0.0 |
| TDTSSAS | Inf | 82 | 0.0 |
| TDTSSEST | Inf | 79 | 0.0 |
| TEHGE.T | Inf | 2 | 0.0 |
| TETHTLS.S | Inf | 68 | 0.0 |
| TFA.APT.D | Inf | 79 | 0.0 |
| TFDPEGQ.P | Inf | 68 | 0.0 |
| TFPSGET.T | Inf | 68 | 0.0 |
| TG..T.LPV | Inf | 82 | 0.0 |
| TG..TPLLV | Inf | 82 | 0.0 |
| TG.AGTIP | Inf | 79 | 0.0 |
| TG.AIPL.V | Inf | 82 | 0.0 |
| TG.ATSLLV | Inf | 82 | 0.0 |
| TG.PST.TA | Inf | 68 | 0.0 |
| TG.QSTT | Inf | 2 | 0.0 |
| TG.SSASTG | Inf | 82 | 0.0 |
| TGDGHTT | Inf | 79 | 0.0 |
| TGDTMP | Inf | 79 | 0.0 |
| TGDTST.P | Inf | 79 | 0.0 |
| TGDTTP | Inf | 82 | 0.0 |
| TGH.TP.P | Inf | 82 | 0.0 |
| TGH.TPLHV | Inf | 82 | 0.0 |
| TGHAS.LL | Inf | 79 | 0.0 |
| TGHATSL | Inf | 82 | 0.0 |
| TGLSS.ST | Inf | 82 | 0.0 |
| TGLSSA.T | Inf | 82 | 0.0 |
| TGLTSPQ | Inf | 68 | 0.0 |
| TGS.TPAP | Inf | 10 | 0.0 |
| TGTQTPTST | Inf | 4 | 0.0 |
| TGTVS..TS | Inf | 79 | 0.0 |
| TGYDL..DA | Inf | 5 | 0.0 |
| TH..EST.A | Inf | 79 | 0.0 |
| THT.Q.E.T | Inf | 79 | 0.0 |
| THT.S.SGS | Inf | 79 | 0.0 |
| TI..DTPE | Inf | 5 | 0.0 |
| TI.SAASTG | Inf | 68 | 0.0 |
| TIPSS.SS | Inf | 79 | 0.0 |
| TK.SS..TG | Inf | 14 | 0.0 |
| TKPSSP.T | Inf | 14 | 0.0 |
| TL.PITTTT | Inf | 2 | 0.0 |
| TL.TGS.TP | Inf | 10 | 0.0 |
| TLSPA..TE | Inf | 2 | 0.0 |
| TM.PITTTT | Inf | 2 | 0.0 |
| TMLTSPL | Inf | 40 | 0.0 |
| TMP.PVTSP | Inf | 79 | 0.0 |
| TN.SSASTG | Inf | 82 | 0.0 |
| TNASS.ST | Inf | 82 | 0.0 |
| TP..IQAK | Inf | 5 | 0.0 |
| TP.ANE.T | Inf | 2 | 0.0 |
| TP.ANE.TT | Inf | 2 | 0.0 |
| TP.DMMS | Inf | 5 | 0.0 |
| TP.PITTTT | Inf | 2 | 0.0 |
| TP.PVTDT | Inf | 82 | 0.0 |
| TPAPVTST | Inf | 10 | 0.0 |
| TPE.ML..M | Inf | 5 | 0.0 |
| TPGMTTPS | Inf | 58 | 0.0 |
| TPI.TTTM | Inf | 2 | 0.0 |
| TPISTTTTV | Inf | 4 | 0.0 |
| TPL.DTD.S | Inf | 68 | 0.0 |
| TPL.L.S.S | Inf | 79 | 0.0 |
| TPL.VT.AS | Inf | 82 | 0.0 |
| TPLHVT | Inf | 82 | 0.0 |
| TPLHVT..S | Inf | 82 | 0.0 |
| TPLLVT.TS | Inf | 79 | 0.0 |
| TPLLVTD | Inf | 82 | 0.0 |
| TPLPGT.T | Inf | 82 | 0.0 |
| TPLPVT.L | Inf | 82 | 0.0 |
| TPLPVT.LS | Inf | 82 | 0.0 |
| TPLPVT.P | Inf | 82 | 0.0 |
| TPLPVTD | Inf | 82 | 0.0 |
| TPLPVTD.S | Inf | 82 | 0.0 |
| TPLPVTP | Inf | 5 | 0.0 |
| TPLPVTSFS | Inf | 79 | 0.0 |
| TPLPVTSSS | Inf | 61 | 0.0 |
| TPLPVTY | Inf | 79 | 0.0 |
| TPPSITST | Inf | 68 | 0.0 |
| TPT.ITTTT | Inf | 4 | 0.0 |
| TPT.T.STT | Inf | 4 | 0.0 |
| TPTPI.TT | Inf | 4 | 0.0 |
| TPVPVT.TS | Inf | 79 | 0.0 |
| TPVPVTST | Inf | 79 | 0.0 |
| TQ.PTPT | Inf | 4 | 0.0 |
| TQ.PTSTP | Inf | 4 | 0.0 |
| TQ.TRTS | Inf | 79 | 0.0 |
| TQA.QTT.E | Inf | 79 | 0.0 |
| TQTPTPT | Inf | 4 | 0.0 |
| TR..TSL.V | Inf | 81 | 0.0 |
| TRLPVT | Inf | 82 | 0.0 |
| TRSVSP.T | Inf | 79 | 0.0 |
| TRTSR.S | Inf | 79 | 0.0 |
| TS..ISTT | Inf | 2 | 0.0 |
| TS..ITTTT | Inf | 4 | 0.0 |
| TS..STGHT | Inf | 82 | 0.0 |
| TS.QTETHT | Inf | 68 | 0.0 |
| TS.SSVST | Inf | 82 | 0.0 |
| TSA.LVTF | Inf | 68 | 0.0 |
| TSA.STGHA | Inf | 82 | 0.0 |
| TSFSSASTG | Inf | 68 | 0.0 |
| TSKVFRM.T | Inf | 68 | 0.0 |
| TSL.VT..S | Inf | 82 | 0.0 |
| TSL.VTD | Inf | 82 | 0.0 |
| TSLFSVS | Inf | 21 | 0.0 |
| TSLHVTS | Inf | 81 | 0.0 |
| TSLPVS.T | Inf | 21 | 0.0 |
| TSLPVTS.S | Inf | 79 | 0.0 |
| TSLSSASTG | Inf | 82 | 0.0 |
| TSLSVS.T | Inf | 79 | 0.0 |
| TSPSAS..E | Inf | 79 | 0.0 |
| TSPSS.STG | Inf | 82 | 0.0 |
| TSPSSASTG | Inf | 82 | 0.0 |
| TSPSSSPM | Inf | 79 | 0.0 |
| TSQTM..S | Inf | 16 | 0.0 |
| TSR.STLGN | Inf | 68 | 0.0 |
| TSRSSASTG | Inf | 58 | 0.0 |
| TSS.STGHV | Inf | 79 | 0.0 |
| TSS.STGQA | Inf | 82 | 0.0 |
| TSSASTGQ | Inf | 82 | 0.0 |
| TSSASTRH | Inf | 58 | 0.0 |
| TSSAYTG | Inf | 79 | 0.0 |
| TSSIST.HA | Inf | 69 | 0.0 |
| TSSPSSVS | Inf | 68 | 0.0 |
| TSSSSAS.G | Inf | 61 | 0.0 |
| TSSVST.HA | Inf | 82 | 0.0 |
| TSSVSTG | Inf | 82 | 0.0 |
| TSTGLTS | Inf | 68 | 0.0 |
| TSTLPVT | Inf | 79 | 0.0 |
| TSTPITTT | Inf | 4 | 0.0 |
| TSTVL.SP | Inf | 79 | 0.0 |
| TT..P.EPT | Inf | 2 | 0.0 |
| TT.LPVT | Inf | 82 | 0.0 |
| TT.MTP..T | Inf | 3 | 0.0 |
| TT.TPITTT | Inf | 4 | 0.0 |
| TTALQA..S | Inf | 79 | 0.0 |
| TTP.P.TDT | Inf | 82 | 0.0 |
| TTPLLVTE | Inf | 79 | 0.0 |
| TTPLPVT | Inf | 82 | 0.0 |
| TTPSP..PT | Inf | 2 | 0.0 |
| TTRFS…T | Inf | 16 | 0.0 |
| TTRSVS..T | Inf | 79 | 0.0 |
| TTSSPSSVS | Inf | 68 | 0.0 |
| TTTMVTPT | Inf | 2 | 0.0 |
| TTTPIS | Inf | 2 | 0.0 |
| TTTSSPSS | Inf | 68 | 0.0 |
| TV..KT.PS | Inf | 79 | 0.0 |
| TV.SSPS.F | Inf | 79 | 0.0 |
| V..AKKAYD | Inf | 5 | 0.0 |
| V..PLPI.M | Inf | 19 | 0.0 |
| V..SD.DYR | Inf | 5 | 0.0 |
| V.AKK.QV | Inf | 5 | 0.0 |
| V.AKKCQV | Inf | 5 | 0.0 |
| V.ETAPPD | Inf | 69 | 0.0 |
| V.NVTGT.S | Inf | 79 | 0.0 |
| V.QAK.NA | Inf | 5 | 0.0 |
| VAQRSV | Inf | 14 | 0.0 |
| VFRMPTS | Inf | 68 | 0.0 |
| VGGPH..R | Inf | 40 | 0.0 |
| VH..M.TAP | Inf | 68 | 0.0 |
| VK.NQE.LS | Inf | 5 | 0.0 |
| VKLNQE..S | Inf | 5 | 0.0 |
| VLSSPS..N | Inf | 79 | 0.0 |
| VME.APP | Inf | 69 | 0.0 |
| VP.TST.L | Inf | 11 | 0.0 |
| VPSKVSA | Inf | 79 | 0.0 |
| VPVTDTS | Inf | 79 | 0.0 |
| VPVTST.SA | Inf | 78 | 0.0 |
| VS..FDG.A | Inf | 25 | 0.0 |
| VSDTSS..T | Inf | 21 | 0.0 |
| VSG.IS..T | Inf | 79 | 0.0 |
| VSI.AG..G | Inf | 79 | 0.0 |
| VSLFP.GAD | Inf | 17 | 0.0 |
| VSPMT.T.T | Inf | 79 | 0.0 |
| VSQEI.T | Inf | 79 | 0.0 |
| VSQRG.TQ | Inf | 79 | 0.0 |
| VST.HAT.L | Inf | 82 | 0.0 |
| VSTGH.TPL | Inf | 82 | 0.0 |
| VT.PSSAS | Inf | 82 | 0.0 |
| VT.TNS.ST | Inf | 79 | 0.0 |
| VT.TPS.ST | Inf | 82 | 0.0 |
| VT.TPTPTS | Inf | 2 | 0.0 |
| VT.TSSVST | Inf | 82 | 0.0 |
| VTD.SS..T | Inf | 82 | 0.0 |
| VTD.SS.ST | Inf | 82 | 0.0 |
| VTD.SSAST | Inf | 82 | 0.0 |
| VTD.SSSST | Inf | 79 | 0.0 |
| VTD.SSVST | Inf | 82 | 0.0 |
| VTDASS.S | Inf | 82 | 0.0 |
| VTDASS.ST | Inf | 82 | 0.0 |
| VTDT.SAS | Inf | 82 | 0.0 |
| VTDTSSAS | Inf | 82 | 0.0 |
| VTDTYSA | Inf | 71 | 0.0 |
| VTETSSAST | Inf | 21 | 0.0 |
| VTFDP.G.S | Inf | 68 | 0.0 |
| VTG.SSA.T | Inf | 82 | 0.0 |
| VTG.SSAST | Inf | 82 | 0.0 |
| VTGNPSTG | Inf | 79 | 0.0 |
| VTI.SS.SS | Inf | 79 | 0.0 |
| VTI.SS.ST | Inf | 79 | 0.0 |
| VTNASS.ST | Inf | 82 | 0.0 |
| VTNTSSAST | Inf | 11 | 0.0 |
| VTPLPVTS | Inf | 79 | 0.0 |
| VTQFS…S | Inf | 19 | 0.0 |
| VTQMIK.A | Inf | 79 | 0.0 |
| VTSAA..TF | Inf | 68 | 0.0 |
| VTSLSS.ST | Inf | 82 | 0.0 |
| VTSPSS.ST | Inf | 82 | 0.0 |
| VTSSSSAS | Inf | 61 | 0.0 |
| VTSSSSASS | Inf | 61 | 0.0 |
| VTSTSSSST | Inf | 10 | 0.0 |
| VTSTSSVST | Inf | 79 | 0.0 |
| VVLAK.N.L | Inf | 5 | 0.0 |
| W..SGTM.T | Inf | 40 | 0.0 |
| W.D.K.T.Y | Inf | 5 | 0.0 |
| W.KDKS.I | Inf | 5 | 0.0 |
| W.PEGSVE | Inf | 5 | 0.0 |
| W.VAQRSV | Inf | 14 | 0.0 |
| WEK.KA.V | Inf | 5 | 0.0 |
| WEKEK.N | Inf | 5 | 0.0 |
| WS..G..WT | Inf | 16 | 0.0 |
| WSC.PD | Inf | 5 | 0.0 |
| WW..SGT | Inf | 40 | 0.0 |
| Y..AWEK.K | Inf | 5 | 0.0 |
| Y..EQQHVG | Inf | 1 | 0.0 |
| Y..PLPTG | Inf | 39 | 0.0 |
| Y.LRAD..G | Inf | 5 | 0.0 |
| Y.SSS.GP | Inf | 25 | 0.0 |
| YCKQLGH | Inf | 5 | 0.0 |
| YDIRADA | Inf | 5 | 0.0 |
| YEK.KGI | Inf | 5 | 0.0 |
| YG.VPGVG | Inf | 8 | 0.0 |
| YGADAGDL | Inf | 17 | 0.0 |
| YGAGAGD | Inf | 46 | 0.0 |
| YGPWG…E | Inf | 25 | 0.0 |
| YK..YC.QL | Inf | 5 | 0.0 |
| YKARW | Inf | 25 | 0.0 |
| YPGF…G | Inf | 14 | 0.0 |
| YQ..W.KDK | Inf | 5 | 0.0 |
| YR.D..AP | Inf | 1 | 0.0 |
| YSVTQ…T | Inf | 79 | 0.0 |
| PLPVT..SS | 93183.26 | 82 | 0.0 |
| TPLPVT | 92520.65 | 87 | 0.0 |
| VT..S.AST | 87918.67 | 82 | 0.0 |
| VT..SSAST | 85840.07 | 82 | 0.0 |
| T..SSASTG | 84405.92 | 82 | 0.0 |
| S.S.GH.TP | 59326.50 | 82 | 0.0 |
| STGHA..L | 55840.97 | 82 | 0.0 |
| SS..TGHAT | 55505.13 | 82 | 0.0 |
| TGHAT.L | 54915.13 | 82 | 0.0 |
| SSAS.GH | 49051.47 | 82 | 0.0 |
| LPVT.TS | 47971.32 | 82 | 0.0 |
| T.LPVT.TS | 47971.32 | 82 | 0.0 |
| S.ASTGH | 45965.33 | 82 | 0.0 |
| SSASTGH | 43886.72 | 82 | 0.0 |
| ATPLPVT | 42516.11 | 82 | 0.0 |
| SASTGH.T | 41435.96 | 82 | 0.0 |
| SASTG.A.P | 41272.58 | 82 | 0.0 |
| TPLPVTS.S | 40700.74 | 82 | 0.0 |
| STGHATP | 40010.89 | 82 | 0.0 |
| T.SASTG | 37560.13 | 82 | 0.0 |
| SS.STG.A | 36511.76 | 82 | 0.0 |
| TSSASTG | 35082.15 | 82 | 0.0 |
| AST.HAT | 33303.08 | 82 | 0.0 |
| TSPSS.S.G | 26776.80 | 82 | 0.0 |
| A.PLPVTS | 23155.12 | 82 | 0.0 |
| HATPL…S | 16892.08 | 82 | 0.0 |
| ATPLP.T.T | 16465.46 | 82 | 0.0 |
| VTSTSS.ST | 10547.34 | 82 | 0.0 |
| LPVTSTSS | 9303.80 | 82 | 0.0 |
| PVTSTS.AS | 9113.19 | 82 | 0.0 |
| TSTSSAS.G | 9013.34 | 82 | 0.0 |
| AT.LP.TST | 5845.51 | 82 | 0.0 |
| GTVSQ.T | 1434.15 | 79 | 0.0 |
| VSQ.T.PSG | 1434.15 | 79 | 0.0 |
| A..SRTS.Q | 1325.22 | 78 | 0.0 |
| TA.PVTST | 1243.53 | 79 | 0.0 |
| TTP.PV..T | 1051.62 | 86 | 0.0 |
| TS..STLGN | 1025.69 | 79 | 0.0 |
| T.STV.PT | 753.38 | 81 | 0.0 |
| TTSTV…P | 753.38 | 81 | 0.0 |
| I.T.NT.T | 735.23 | 81 | 0.0 |
| T..MTTPS | 735.23 | 81 | 0.0 |
| S.SPDTA | 726.15 | 80 | 0.0 |
| SP.EIVP | 726.15 | 80 | 0.0 |
| SRTTS..T | 726.15 | 81 | 0.0 |
| A.SSATSA | 717.07 | 79 | 0.0 |
| AAAMTHT | 717.07 | 79 | 0.0 |
| AAISR.A.T | 717.07 | 79 | 0.0 |
| AAPIP..PE | 717.07 | 79 | 0.0 |
| AAT.L..GN | 717.07 | 79 | 0.0 |
| AATTFA..P | 717.07 | 79 | 0.0 |
| AE.TGRP.E | 717.07 | 79 | 0.0 |
| AESTEAS | 717.07 | 79 | 0.0 |
| AFH.Q.S.G | 717.07 | 79 | 0.0 |
| AFSKN..TQ | 717.07 | 79 | 0.0 |
| AG..P.VPS | 717.07 | 79 | 0.0 |
| AGA.T.WTQ | 717.07 | 79 | 0.0 |
| AGRPTG | 717.07 | 79 | 0.0 |
| AGTES.TPV | 717.07 | 79 | 0.0 |
| AIGEPGE | 717.07 | 79 | 0.0 |
| AIPVS.G.A | 717.07 | 79 | 0.0 |
| AIS.MA.T | 717.07 | 79 | 0.0 |
| AITSK..T | 717.07 | 79 | 0.0 |
| AL.SN.TP | 717.07 | 79 | 0.0 |
| ALLSSP..S | 717.07 | 79 | 0.0 |
| ALSPSS.P | 717.07 | 79 | 0.0 |
| ALT.ANS | 717.07 | 79 | 0.0 |
| AM.TR.T.A | 717.07 | 79 | 0.0 |
| ANS.VSTP | 717.07 | 79 | 0.0 |
| AP..N.STI | 717.07 | 79 | 0.0 |
| AP.PI.PE | 717.07 | 79 | 0.0 |
| APQTTQ.S | 717.07 | 79 | 0.0 |
| APS.H..TL | 717.07 | 79 | 0.0 |
| APTISA.TT | 717.07 | 79 | 0.0 |
| AQTQW…T | 717.07 | 79 | 0.0 |
| AS..AIPVS | 717.07 | 79 | 0.0 |
| AS.AG.PTG | 717.07 | 79 | 0.0 |
| AS.QETAA | 717.07 | 79 | 0.0 |
| AS.TTPS.S | 717.07 | 79 | 0.0 |
| ASA.HGA | 717.07 | 79 | 0.0 |
| ASASTSPD | 717.07 | 79 | 0.0 |
| ASG.TQTS | 717.07 | 79 | 0.0 |
| ASGHSPS | 717.07 | 79 | 0.0 |
| ASQAT.T.S | 717.07 | 79 | 0.0 |
| ASS.VPGT | 717.07 | 79 | 0.0 |
| ASSGS.TTS | 717.07 | 79 | 0.0 |
| AST.SS.SP | 717.07 | 79 | 0.0 |
| ASTSP.TA | 717.07 | 79 | 0.0 |
| AT.LVT.N | 717.07 | 79 | 0.0 |
| AT.SSS.AS | 717.07 | 79 | 0.0 |
| ATDTFS | 717.07 | 79 | 0.0 |
| ATL.PS.GT | 717.07 | 79 | 0.0 |
| ATP.AVSS | 717.07 | 79 | 0.0 |
| ATS.P.TTS | 717.07 | 79 | 0.0 |
| ATS.S.VSS | 717.07 | 79 | 0.0 |
| ATSSL..VT | 717.07 | 79 | 0.0 |
| ATTFAP..T | 717.07 | 79 | 0.0 |
| AVSITA | 717.07 | 79 | 0.0 |
| AVSSATS | 717.07 | 79 | 0.0 |
| DA..GP.GG | 717.07 | 79 | 0.0 |
| DA.TISA.T | 717.07 | 79 | 0.0 |
| DEMTTS..S | 717.07 | 79 | 0.0 |
| DGGRRT | 717.07 | 79 | 0.0 |
| DR.TS..IT | 717.07 | 79 | 0.0 |
| DS..T.STT | 717.07 | 79 | 0.0 |
| DSTLGNT | 717.07 | 79 | 0.0 |
| DT.AA.T.T | 717.07 | 79 | 0.0 |
| DT.STALS | 717.07 | 79 | 0.0 |
| DT.TVTT.G | 717.07 | 79 | 0.0 |
| DTISL.S.A | 717.07 | 79 | 0.0 |
| DTTAFS | 717.07 | 79 | 0.0 |
| E..PQ.APT | 717.07 | 79 | 0.0 |
| E.PSTST.A | 717.07 | 79 | 0.0 |
| E.S.QTQT | 717.07 | 79 | 0.0 |
| E.T.TAGT | 717.07 | 79 | 0.0 |
| EA.LSSPS | 717.07 | 79 | 0.0 |
| EASTAGR | 717.07 | 79 | 0.0 |
| EG..TSASA | 717.07 | 79 | 0.0 |
| EG..TSG.T | 717.07 | 79 | 0.0 |
| EGA.TTG.P | 717.07 | 79 | 0.0 |
| EGQSRT | 717.07 | 79 | 0.0 |
| EI.TL..TT | 717.07 | 79 | 0.0 |
| EK.TVT..T | 717.07 | 79 | 0.0 |
| EL.SASAS | 717.07 | 79 | 0.0 |
| EMTTS.P.S | 717.07 | 79 | 0.0 |
| EPASS.S.T | 717.07 | 79 | 0.0 |
| EPGEPT | 717.07 | 79 | 0.0 |
| EPTTY.S | 717.07 | 79 | 0.0 |
| ER.SFSP.V | 717.07 | 79 | 0.0 |
| ESP.VSQ | 717.07 | 79 | 0.0 |
| ESQTST.T | 717.07 | 79 | 0.0 |
| ESSTPVTS | 717.07 | 79 | 0.0 |
| ESTEAS..T | 717.07 | 79 | 0.0 |
| ET.SVP.T | 717.07 | 79 | 0.0 |
| ETAAIS | 717.07 | 79 | 0.0 |
| ETATSS..S | 717.07 | 79 | 0.0 |
| ETFPS..TT | 717.07 | 79 | 0.0 |
| ETGTTGE | 717.07 | 79 | 0.0 |
| ETT.FS.N | 717.07 | 79 | 0.0 |
| ETT.R..ER | 717.07 | 79 | 0.0 |
| ETT.W..SF | 717.07 | 79 | 0.0 |
| ETTRVS.I | 717.07 | 79 | 0.0 |
| F..SGHSPS | 717.07 | 79 | 0.0 |
| F.PSGT.SQ | 717.07 | 79 | 0.0 |
| FH.TLS.AS | 717.07 | 79 | 0.0 |
| FHT.QS.G | 717.07 | 79 | 0.0 |
| FLVTS.V | 717.07 | 79 | 0.0 |
| FPS.VT.TL | 717.07 | 79 | 0.0 |
| FS..H.TQS | 717.07 | 79 | 0.0 |
| FS.TST.DT | 717.07 | 79 | 0.0 |
| FSSKG.TT | 717.07 | 79 | 0.0 |
| FSTVPPT.P | 717.07 | 79 | 0.0 |
| FTL.ETTT | 717.07 | 79 | 0.0 |
| FTT.LIS | 717.07 | 79 | 0.0 |
| G..AQTQ.T | 717.07 | 79 | 0.0 |
| G..GETSS | 717.07 | 79 | 0.0 |
| G..RTATSP | 717.07 | 79 | 0.0 |
| G.A.LSSPS | 717.07 | 79 | 0.0 |
| G.RTATSP | 717.07 | 79 | 0.0 |
| G.S.SGET | 717.07 | 79 | 0.0 |
| G.SRTTS | 717.07 | 79 | 0.0 |
| G.SSP.SPS | 717.07 | 79 | 0.0 |
| G.TQTS.P | 717.07 | 79 | 0.0 |
| G.TTQ.PTT | 717.07 | 79 | 0.0 |
| G.TTWS.T | 717.07 | 79 | 0.0 |
| GA..VSTG | 717.07 | 79 | 0.0 |
| GAATR.V.G | 717.07 | 79 | 0.0 |
| GAE.TG.P | 717.07 | 79 | 0.0 |
| GALTL.NS | 717.07 | 79 | 0.0 |
| GAQ.Q.TQE | 717.07 | 79 | 0.0 |
| GASGTTPS | 717.07 | 79 | 0.0 |
| GE.TTYSS | 717.07 | 79 | 0.0 |
| GEPGE.TT | 717.07 | 79 | 0.0 |
| GETATS | 717.07 | 79 | 0.0 |
| GETTR.S | 717.07 | 79 | 0.0 |
| GF..SGT.S | 717.07 | 79 | 0.0 |
| GGPEG.W.S | 717.07 | 79 | 0.0 |
| GGTSLS..G | 717.07 | 79 | 0.0 |
| GH.PS..VP | 717.07 | 79 | 0.0 |
| GH.TPLAV | 717.07 | 79 | 0.0 |
| GKTFTT.L | 717.07 | 79 | 0.0 |
| GMASS..P | 717.07 | 79 | 0.0 |
| GMTTPS..T | 717.07 | 79 | 0.0 |
| GN..ETS.A | 717.07 | 79 | 0.0 |
| GNT.ETS.S | 717.07 | 79 | 0.0 |
| GPEGQ..SA | 717.07 | 79 | 0.0 |
| GPSGGTS.S | 717.07 | 79 | 0.0 |
| GQWTS…S | 717.07 | 79 | 0.0 |
| GRPHE.SS | 717.07 | 79 | 0.0 |
| GRPTG.SS | 717.07 | 79 | 0.0 |
| GSDTISL.S | 717.07 | 79 | 0.0 |
| GSEGIS.S | 717.07 | 79 | 0.0 |
| GSGKT.TT | 717.07 | 79 | 0.0 |
| GSL.PVTS | 717.07 | 79 | 0.0 |
| GSSFT.SG | 717.07 | 79 | 0.0 |
| GSTLG.PG | 717.07 | 79 | 0.0 |
| GT..PSSSG | 717.07 | 79 | 0.0 |
| GT.SA.TSK | 717.07 | 79 | 0.0 |
| GTF.P.LSE | 717.07 | 79 | 0.0 |
| GTIPR.PS | 717.07 | 79 | 0.0 |
| GTSLS.T.A | 717.07 | 79 | 0.0 |
| GTTGEA | 717.07 | 79 | 0.0 |
| GTTPSGS | 717.07 | 79 | 0.0 |
| GTVSQE.F | 717.07 | 79 | 0.0 |
| H..TL.PSG | 717.07 | 79 | 0.0 |
| H.TTSTPS | 717.07 | 79 | 0.0 |
| HE.SS.SPG | 717.07 | 79 | 0.0 |
| HE.TT.PSS | 717.07 | 79 | 0.0 |
| HGA.PVST | 717.07 | 79 | 0.0 |
| HPT.SE.ST | 717.07 | 79 | 0.0 |
| HST.HST | 717.07 | 79 | 0.0 |
| HSTSTSP | 717.07 | 79 | 0.0 |
| HSTTL..TT | 717.07 | 79 | 0.0 |
| HT..SEG.E | 717.07 | 79 | 0.0 |
| HTRST..P | 717.07 | 79 | 0.0 |
| HTS.Q.TT | 717.07 | 79 | 0.0 |
| HTT.APTT | 717.07 | 79 | 0.0 |
| HTT.S.TEL | 717.07 | 79 | 0.0 |
| HWT.ST.TT | 717.07 | 79 | 0.0 |
| I.STA.HT | 717.07 | 79 | 0.0 |
| I.T.H.TTT | 717.07 | 79 | 0.0 |
| IGEPGEP | 717.07 | 79 | 0.0 |
| IHS.STSP | 717.07 | 79 | 0.0 |
| IISTIPS | 717.07 | 79 | 0.0 |
| IP.LPE.G | 717.07 | 79 | 0.0 |
| IPVST..AS | 717.07 | 79 | 0.0 |
| IQDTSA | 717.07 | 79 | 0.0 |
| IS..TPLPV | 717.07 | 79 | 0.0 |
| IS.AS.AT | 717.07 | 79 | 0.0 |
| IS.ATT..P | 717.07 | 79 | 0.0 |
| ISAITSK | 717.07 | 79 | 0.0 |
| ISRMA..Q | 717.07 | 79 | 0.0 |
| IST.P.TA | 717.07 | 79 | 0.0 |
| ISTAFH | 717.07 | 79 | 0.0 |
| ISTSGET | 717.07 | 79 | 0.0 |
| IT.TTST.S | 717.07 | 79 | 0.0 |
| ITSKVST | 717.07 | 79 | 0.0 |
| ITTAPST | 717.07 | 79 | 0.0 |
| IVPGTF.P | 717.07 | 79 | 0.0 |
| IVPQD.P | 717.07 | 79 | 0.0 |
| IW..DTL.T | 717.07 | 79 | 0.0 |
| K.STAF.T | 717.07 | 79 | 0.0 |
| K.STI.WS | 717.07 | 79 | 0.0 |
| KG.TT..QT | 717.07 | 79 | 0.0 |
| KIT.TTST | 717.07 | 79 | 0.0 |
| KNH.T.S.E | 717.07 | 79 | 0.0 |
| KT.TM.TST | 717.07 | 79 | 0.0 |
| KT.TTP.SS | 717.07 | 79 | 0.0 |
| KTDGGR | 717.07 | 79 | 0.0 |
| KTFTT..IS | 717.07 | 79 | 0.0 |
| KTGALT.A | 717.07 | 79 | 0.0 |
| KTS.SG.TA | 717.07 | 79 | 0.0 |
| KTTGAGA | 717.07 | 79 | 0.0 |
| L.E.TT.PS | 717.07 | 79 | 0.0 |
| L.T.TPVTT | 717.07 | 79 | 0.0 |
| L.TALSP.S | 717.07 | 79 | 0.0 |
| LAN.V..TP | 717.07 | 79 | 0.0 |
| LASQATD | 717.07 | 79 | 0.0 |
| LCSVT.TS | 717.07 | 79 | 0.0 |
| LDRHTS | 717.07 | 79 | 0.0 |
| LG.TE.TS | 717.07 | 79 | 0.0 |
| LGNPGET | 717.07 | 79 | 0.0 |
| LGPSG..SL | 717.07 | 79 | 0.0 |
| LIS.A.PLP | 717.07 | 79 | 0.0 |
| LKTDG.R | 717.07 | 79 | 0.0 |
| LLSAS.SH | 717.07 | 79 | 0.0 |
| LLSSPSYS | 717.07 | 79 | 0.0 |
| LM.TS.TIT | 717.07 | 79 | 0.0 |
| LPKTT..GA | 717.07 | 79 | 0.0 |
| LPP.ISTA | 717.07 | 79 | 0.0 |
| LPSTST..A | 717.07 | 79 | 0.0 |
| LSASAS..A | 717.07 | 79 | 0.0 |
| LSEASTA | 717.07 | 79 | 0.0 |
| LSKTG..T | 717.07 | 79 | 0.0 |
| LSPSGS..T | 717.07 | 79 | 0.0 |
| LSPSS.PP | 717.07 | 79 | 0.0 |
| LTLANS.VS | 717.07 | 79 | 0.0 |
| LVTSK..R | 717.07 | 79 | 0.0 |
| M.T.KTIT | 717.07 | 79 | 0.0 |
| MASSI..GT | 717.07 | 79 | 0.0 |
| MHT.ST..P | 717.07 | 79 | 0.0 |
| MLD.HTS | 717.07 | 79 | 0.0 |
| MMTSE..T | 717.07 | 79 | 0.0 |
| MT..K.TVT | 717.07 | 79 | 0.0 |
| MT..KT.TT | 717.07 | 79 | 0.0 |
| MT.SF..SV | 717.07 | 79 | 0.0 |
| MTS.TIT.T | 717.07 | 79 | 0.0 |
| MTTPSL.T | 717.07 | 79 | 0.0 |
| N.STI.STS | 717.07 | 79 | 0.0 |
| NH.TRST.T | 717.07 | 79 | 0.0 |
| NHQ.Q..ET | 717.07 | 79 | 0.0 |
| NPG.TSS.P | 717.07 | 79 | 0.0 |
| NPSGT.S | 717.07 | 79 | 0.0 |
| NS.VSTP | 717.07 | 79 | 0.0 |
| NT..T.TPV | 717.07 | 79 | 0.0 |
| NT.TP.TT | 717.07 | 79 | 0.0 |
| NTFLVTS | 717.07 | 79 | 0.0 |
| P..QWT.AS | 717.07 | 79 | 0.0 |
| PASSGS..T | 717.07 | 79 | 0.0 |
| PAVSQR..T | 717.07 | 79 | 0.0 |
| PD..TTSFP | 717.07 | 79 | 0.0 |
| PDT.AA.T | 717.07 | 79 | 0.0 |
| PEGQSP.T | 717.07 | 79 | 0.0 |
| PG.PTT.SS | 717.07 | 79 | 0.0 |
| PGETSSV | 717.07 | 79 | 0.0 |
| PGGPEGQ | 717.07 | 79 | 0.0 |
| PGSSFT.S | 717.07 | 79 | 0.0 |
| PGTFH.T.S | 717.07 | 79 | 0.0 |
| PHERSS..P | 717.07 | 79 | 0.0 |
| PILPER | 717.07 | 79 | 0.0 |
| PIP.LP..G | 717.07 | 79 | 0.0 |
| PKIST…T | 717.07 | 79 | 0.0 |
| PKT.GAG | 717.07 | 79 | 0.0 |
| PLA.SS.TS | 717.07 | 79 | 0.0 |
| PML.R.TS | 717.07 | 79 | 0.0 |
| PMT.T.TVT | 717.07 | 79 | 0.0 |
| PPK.STA | 717.07 | 79 | 0.0 |
| PPPTTS.T | 717.07 | 79 | 0.0 |
| PPTPPSI | 717.07 | 79 | 0.0 |
| PPTTSQT | 717.07 | 79 | 0.0 |
| PQ.TA.IS | 717.07 | 79 | 0.0 |
| PQ.TQES.T | 717.07 | 79 | 0.0 |
| PQDAPTI | 717.07 | 79 | 0.0 |
| PQESPAV | 717.07 | 79 | 0.0 |
| PS..PSVH | 717.07 | 79 | 0.0 |
| PS.AM.T.S | 717.07 | 79 | 0.0 |
| PSEIVPQ | 717.07 | 79 | 0.0 |
| PSGETA.SS | 717.07 | 79 | 0.0 |
| PSGFN.S | 717.07 | 79 | 0.0 |
| PSGGTS.S | 717.07 | 79 | 0.0 |
| PSGSEGI | 717.07 | 79 | 0.0 |
| PSGSG.T.T | 717.07 | 79 | 0.0 |
| PSLKTDG | 717.07 | 79 | 0.0 |
| PSS.D.TLG | 717.07 | 79 | 0.0 |
| PSS.S.KG | 717.07 | 79 | 0.0 |
| PSSLPPK.S | 717.07 | 79 | 0.0 |
| PSSSGA.GT | 717.07 | 79 | 0.0 |
| PSSSPML | 717.07 | 79 | 0.0 |
| PSSVSNT | 717.07 | 79 | 0.0 |
| PSSVT.TL | 717.07 | 79 | 0.0 |
| PSTNH.T | 717.07 | 79 | 0.0 |
| PSTST.A.T | 717.07 | 79 | 0.0 |
| PSV.NVT.T | 717.07 | 79 | 0.0 |
| PT..SSHST | 717.07 | 79 | 0.0 |
| PT.SEASTA | 717.07 | 79 | 0.0 |
| PTG.SSP.S | 717.07 | 79 | 0.0 |
| PTISA.TT | 717.07 | 79 | 0.0 |
| PTTSQT..S | 717.07 | 79 | 0.0 |
| PVSTG..SS | 717.07 | 79 | 0.0 |
| PVT.SL.PV | 717.07 | 79 | 0.0 |
| PVTSAVS.T | 717.07 | 79 | 0.0 |
| PVTTSTV.S | 717.07 | 79 | 0.0 |
| Q..ETT.VS | 717.07 | 79 | 0.0 |
| Q..KTATS | 717.07 | 79 | 0.0 |
| Q.APT.SA | 717.07 | 79 | 0.0 |
| Q.S.GA.TT | 717.07 | 79 | 0.0 |
| Q.T.ET.TT | 717.07 | 79 | 0.0 |
| Q.T.PSGET | 717.07 | 79 | 0.0 |
| Q.TDT.ST | 717.07 | 79 | 0.0 |
| Q.TGTT.E | 717.07 | 79 | 0.0 |
| Q.TTE..SA | 717.07 | 79 | 0.0 |
| QAPTTA.Q | 717.07 | 79 | 0.0 |
| QDT.A.S.N | 717.07 | 79 | 0.0 |
| QEI.TL..T | 717.07 | 79 | 0.0 |
| QES.A.SQ | 717.07 | 79 | 0.0 |
| QES.TT.S | 717.07 | 79 | 0.0 |
| QI.TL.TLT | 717.07 | 79 | 0.0 |
| QITTA.ST | 717.07 | 79 | 0.0 |
| QQ.TTA.ST | 717.07 | 79 | 0.0 |
| QSEGAE..G | 717.07 | 79 | 0.0 |
| QSS.TSPS | 717.07 | 79 | 0.0 |
| QTE.PST.T | 717.07 | 79 | 0.0 |
| QTI.ST..S | 717.07 | 79 | 0.0 |
| QTQ.TQ.TG | 717.07 | 79 | 0.0 |
| QTQSV.TT | 717.07 | 79 | 0.0 |
| QTQTS.P.S | 717.07 | 79 | 0.0 |
| QTSEP.SS | 717.07 | 79 | 0.0 |
| QTSTL.H | 717.07 | 79 | 0.0 |
| QTT.ESQT | 717.07 | 79 | 0.0 |
| QWTSASAS | 717.07 | 79 | 0.0 |
| R.TATS.PP | 717.07 | 79 | 0.0 |
| RDS.TT.ST | 717.07 | 79 | 0.0 |
| RES.TST.T | 717.07 | 79 | 0.0 |
| RGSDT.S.A | 717.07 | 79 | 0.0 |
| RHT.Q..TT | 717.07 | 79 | 0.0 |
| RLVTG.P.T | 717.07 | 79 | 0.0 |
| RP..QS.PT | 717.07 | 79 | 0.0 |
| RP..RSSFS | 717.07 | 79 | 0.0 |
| RS..TTR.S | 717.07 | 79 | 0.0 |
| RSSFSPG | 717.07 | 79 | 0.0 |
| RST.APIP | 717.07 | 79 | 0.0 |
| RTATS.PPT | 717.07 | 79 | 0.0 |
| RTS.Q.T.A | 717.07 | 79 | 0.0 |
| RTTSAGTA | 717.07 | 79 | 0.0 |
| RTTSTPS.S | 717.07 | 79 | 0.0 |
| RTTSW..S | 717.07 | 79 | 0.0 |
| RVSQI…N | 717.07 | 79 | 0.0 |
| S..VPQD.P | 717.07 | 79 | 0.0 |
| S.DSTLG.T | 717.07 | 79 | 0.0 |
| S.ETT.VS | 717.07 | 79 | 0.0 |
| S.IGEPGE | 717.07 | 79 | 0.0 |
| S.K.DGGR | 717.07 | 79 | 0.0 |
| S.S.TTSAG | 717.07 | 79 | 0.0 |
| SA.TAT.SS | 717.07 | 79 | 0.0 |
| SAATT..PA | 717.07 | 79 | 0.0 |
| SAI.S.VST | 717.07 | 79 | 0.0 |
| SASASHGA | 717.07 | 79 | 0.0 |
| SASAST.P | 717.07 | 79 | 0.0 |
| SASHGAIP | 717.07 | 79 | 0.0 |
| SASTSP.T | 717.07 | 79 | 0.0 |
| SASTVSS | 717.07 | 79 | 0.0 |
| SATSAST.S | 717.07 | 79 | 0.0 |
| SAVSIT | 717.07 | 79 | 0.0 |
| SDSPLK.E | 717.07 | 79 | 0.0 |
| SDT.S.ASQ | 717.07 | 79 | 0.0 |
| SDTLSTA.S | 717.07 | 79 | 0.0 |
| SE.IT.TTS | 717.07 | 79 | 0.0 |
| SE.ST.G.P | 717.07 | 79 | 0.0 |
| SEG.ETT | 717.07 | 79 | 0.0 |
| SEGISTS | 717.07 | 79 | 0.0 |
| SEP.SSGS | 717.07 | 79 | 0.0 |
| SFPSS.T.T | 717.07 | 79 | 0.0 |
| SFS.K..TT | 717.07 | 79 | 0.0 |
| SFSPS.H.V | 717.07 | 79 | 0.0 |
| SFT.SGHS | 717.07 | 79 | 0.0 |
| SGASGT..S | 717.07 | 79 | 0.0 |
| SGETA.SS | 717.07 | 79 | 0.0 |
| SGETT..SS | 717.07 | 79 | 0.0 |
| SGFNPSG.V | 717.07 | 79 | 0.0 |
| SGGTSL..T | 717.07 | 79 | 0.0 |
| SGH.PS..V | 717.07 | 79 | 0.0 |
| SGKTFTT | 717.07 | 79 | 0.0 |
| SGQTQ.S | 717.07 | 79 | 0.0 |
| SGSEG.STS | 717.07 | 79 | 0.0 |
| SGSGK..TT | 717.07 | 79 | 0.0 |
| SGTTPSG | 717.07 | 79 | 0.0 |
| SGTVS..TF | 717.07 | 79 | 0.0 |
| SH..IPVST | 717.07 | 79 | 0.0 |
| SHDATL.P | 717.07 | 79 | 0.0 |
| SHSTT.P.T | 717.07 | 79 | 0.0 |
| SHT.QSTT | 717.07 | 79 | 0.0 |
| SIVPG.F.P | 717.07 | 79 | 0.0 |
| SKG.TT.S | 717.07 | 79 | 0.0 |
| SKTGALT | 717.07 | 79 | 0.0 |
| SKTIT.TTS | 717.07 | 79 | 0.0 |
| SKVSTI..S | 717.07 | 79 | 0.0 |
| SLASQA..T | 717.07 | 79 | 0.0 |
| SLC.VT.TS | 717.07 | 79 | 0.0 |
| SLPPKI.T | 717.07 | 79 | 0.0 |
| SLS.T.ALT | 717.07 | 79 | 0.0 |
| SM.TS..IT | 717.07 | 79 | 0.0 |
| SNATP.PVT | 717.07 | 79 | 0.0 |
| SNT.LVTS | 717.07 | 79 | 0.0 |
| SP..FSRT | 717.07 | 79 | 0.0 |
| SP.TDT.T | 717.07 | 79 | 0.0 |
| SPAVSQR | 717.07 | 79 | 0.0 |
| SPDTA…T | 717.07 | 79 | 0.0 |
| SPMLD.H | 717.07 | 79 | 0.0 |
| SPPPTTSQ | 717.07 | 79 | 0.0 |
| SPQ.SP.VS | 717.07 | 79 | 0.0 |
| SPQ.T.A.S | 717.07 | 79 | 0.0 |
| SPS.ET.T | 717.07 | 79 | 0.0 |
| SPSEIVP | 717.07 | 79 | 0.0 |
| SPSGSGKT | 717.07 | 79 | 0.0 |
| SPSSLPPK | 717.07 | 79 | 0.0 |
| SPSSVS.T | 717.07 | 79 | 0.0 |
| SQ.ITT.PS | 717.07 | 79 | 0.0 |
| SQ.TDT.ST | 717.07 | 79 | 0.0 |
| SQ.TS..GE | 717.07 | 79 | 0.0 |
| SQE.FTL | 717.07 | 79 | 0.0 |
| SQETFP..E | 717.07 | 79 | 0.0 |
| SQI.TL.T | 717.07 | 79 | 0.0 |
| SQNHW.R | 717.07 | 79 | 0.0 |
| SQT.IST.P | 717.07 | 79 | 0.0 |
| SQTEL.S.S | 717.07 | 79 | 0.0 |
| SQTSTLT | 717.07 | 79 | 0.0 |
| SRGSDT.S | 717.07 | 79 | 0.0 |
| SRM.QT..T | 717.07 | 79 | 0.0 |
| SRT.SW.TS | 717.07 | 79 | 0.0 |
| SRTST..TT | 717.07 | 79 | 0.0 |
| SRTTSA.TA | 717.07 | 79 | 0.0 |
| SS.GHTT.S | 717.07 | 79 | 0.0 |
| SSATSA.T | 717.07 | 79 | 0.0 |
| SSDSPL.M | 717.07 | 79 | 0.0 |
| SSFSS.G.T | 717.07 | 79 | 0.0 |
| SSFTA.G.S | 717.07 | 79 | 0.0 |
| SSGASGTT | 717.07 | 79 | 0.0 |
| SSGSRTT | 717.07 | 79 | 0.0 |
| SSH.AT.GP | 717.07 | 79 | 0.0 |
| SSHSTT.P | 717.07 | 79 | 0.0 |
| SSIVPGT | 717.07 | 79 | 0.0 |
| SSLCS.T.T | 717.07 | 79 | 0.0 |
| SSLPPK.S | 717.07 | 79 | 0.0 |
| SSPMLD..T | 717.07 | 79 | 0.0 |
| SSPSSVS.T | 717.07 | 79 | 0.0 |
| SSSGAS..T | 717.07 | 79 | 0.0 |
| SSSPM.D | 717.07 | 79 | 0.0 |
| SSVPVTG | 717.07 | 79 | 0.0 |
| SSVSNTFL | 717.07 | 79 | 0.0 |
| ST.APIPI | 717.07 | 79 | 0.0 |
| ST.HST..S | 717.07 | 79 | 0.0 |
| ST.MHT.S | 717.07 | 79 | 0.0 |
| STAFHT..S | 717.07 | 79 | 0.0 |
| STAGRPT | 717.07 | 79 | 0.0 |
| STAGT.S.T | 717.07 | 79 | 0.0 |
| STALS..S | 717.07 | 79 | 0.0 |
| STEASG.TQ | 717.07 | 79 | 0.0 |
| STG.ASS.V | 717.07 | 79 | 0.0 |
| STGAAT.L | 717.07 | 79 | 0.0 |
| STGST.G.P | 717.07 | 79 | 0.0 |
| STIPSTA | 717.07 | 79 | 0.0 |
| STIWW…L | 717.07 | 79 | 0.0 |
| STL.N..ET | 717.07 | 79 | 0.0 |
| STLTH..TS | 717.07 | 79 | 0.0 |
| STPGGPEG | 717.07 | 79 | 0.0 |
| STPSFSPS | 717.07 | 79 | 0.0 |
| STPVTSA.S | 717.07 | 79 | 0.0 |
| STQTT.ES | 717.07 | 79 | 0.0 |
| STSGETT | 717.07 | 79 | 0.0 |
| STSPQES | 717.07 | 79 | 0.0 |
| STSTGA.T | 717.07 | 79 | 0.0 |
| STSTSP..S | 717.07 | 79 | 0.0 |
| STTEL.S.S | 717.07 | 79 | 0.0 |
| STTLPKTT | 717.07 | 79 | 0.0 |
| STVPPT.P | 717.07 | 79 | 0.0 |
| STVSSD.P | 717.07 | 79 | 0.0 |
| SVH.VTGT | 717.07 | 79 | 0.0 |
| SVPVTGS | 717.07 | 79 | 0.0 |
| SVS.TF.VT | 717.07 | 79 | 0.0 |
| SVTNT.M | 717.07 | 79 | 0.0 |
| SVTNT.M.T | 717.07 | 79 | 0.0 |
| SVV.TPG.P | 717.07 | 79 | 0.0 |
| SWRTSI..T | 717.07 | 79 | 0.0 |
| T…GTTGE | 717.07 | 79 | 0.0 |
| T..GAQTQ | 717.07 | 79 | 0.0 |
| T..LLSASA | 717.07 | 79 | 0.0 |
| T.AATR.VT | 717.07 | 79 | 0.0 |
| T.GNPGET | 717.07 | 79 | 0.0 |
| T.NTL.P.T | 717.07 | 79 | 0.0 |
| T.S.HSTT | 717.07 | 79 | 0.0 |
| T.S.MPV.S | 717.07 | 79 | 0.0 |
| T.SGHSPS | 717.07 | 79 | 0.0 |
| T.TFS..PP | 717.07 | 79 | 0.0 |
| T.TTR.SQ | 717.07 | 79 | 0.0 |
| T.TTST.ST | 717.07 | 79 | 0.0 |
| TA..IPI.P | 717.07 | 79 | 0.0 |
| TA.IS..AQ | 717.07 | 79 | 0.0 |
| TA.SPSS.P | 717.07 | 79 | 0.0 |
| TA.TE.ST | 717.07 | 79 | 0.0 |
| TAAAMT.T | 717.07 | 79 | 0.0 |
| TAF.T..SE | 717.07 | 79 | 0.0 |
| TAFSK…T | 717.07 | 79 | 0.0 |
| TAGRP…S | 717.07 | 79 | 0.0 |
| TAL.S.ATP | 717.07 | 79 | 0.0 |
| TAM.T.STA | 717.07 | 79 | 0.0 |
| TAPSTN.ST | 717.07 | 79 | 0.0 |
| TAT.PPPTT | 717.07 | 79 | 0.0 |
| TATPSSSG | 717.07 | 79 | 0.0 |
| TATSSL.S | 717.07 | 79 | 0.0 |
| TDGGR…T | 717.07 | 79 | 0.0 |
| TDT.T.TTP | 717.07 | 79 | 0.0 |
| TEASG.T.T | 717.07 | 79 | 0.0 |
| TEE.SLS.S | 717.07 | 79 | 0.0 |
| TEE.STA.T | 717.07 | 79 | 0.0 |
| TEL.S..AS | 717.07 | 79 | 0.0 |
| TELPS.ST | 717.07 | 79 | 0.0 |
| TESSTP..S | 717.07 | 79 | 0.0 |
| TF..TSTQ | 717.07 | 79 | 0.0 |
| TF.PTLSE | 717.07 | 79 | 0.0 |
| TFL.TS.VF | 717.07 | 79 | 0.0 |
| TFS.VP.T | 717.07 | 79 | 0.0 |
| TFTTA..S | 717.07 | 79 | 0.0 |
| TG.SSP.SP | 717.07 | 79 | 0.0 |
| TGA.TL..S | 717.07 | 79 | 0.0 |
| TGE.L.SS | 717.07 | 79 | 0.0 |
| TGH.TP.A | 717.07 | 79 | 0.0 |
| TGMASS..P | 717.07 | 79 | 0.0 |
| TGR.H..SS | 717.07 | 79 | 0.0 |
| TGS.LG.PG | 717.07 | 79 | 0.0 |
| TGTTGE | 717.07 | 79 | 0.0 |
| TH.TTSTP | 717.07 | 79 | 0.0 |
| TI..SDT.S | 717.07 | 79 | 0.0 |
| TI.STST | 717.07 | 79 | 0.0 |
| TIIST..ST | 717.07 | 79 | 0.0 |
| TIPST.M.T | 717.07 | 79 | 0.0 |
| TIS.AS.AT | 717.07 | 79 | 0.0 |
| TISAATT | 717.07 | 79 | 0.0 |
| TISAITS | 717.07 | 79 | 0.0 |
| TIT.TTST | 717.07 | 79 | 0.0 |
| TKT.TTPGS | 717.07 | 79 | 0.0 |
| TL..TS.TI | 717.07 | 79 | 0.0 |
| TL.PS.GTS | 717.07 | 79 | 0.0 |
| TLANS..ST | 717.07 | 79 | 0.0 |
| TLGNT..TS | 717.07 | 79 | 0.0 |
| TLHETTT | 717.07 | 79 | 0.0 |
| TLPKTT | 717.07 | 79 | 0.0 |
| TLS.ASTA | 717.07 | 79 | 0.0 |
| TLSPSGSG | 717.07 | 79 | 0.0 |
| TLSTALS | 717.07 | 79 | 0.0 |
| TLT.R.TST | 717.07 | 79 | 0.0 |
| TN.STI.ST | 717.07 | 79 | 0.0 |
| TPG.PEGQ | 717.07 | 79 | 0.0 |
| TPGSS..AS | 717.07 | 79 | 0.0 |
| TPL.VSS.T | 717.07 | 79 | 0.0 |
| TPSFSPSV | 717.07 | 79 | 0.0 |
| TPSGS.G.S | 717.07 | 79 | 0.0 |
| TPSLKT..G | 717.07 | 79 | 0.0 |
| TPSSS..SG | 717.07 | 79 | 0.0 |
| TPVTSA.S | 717.07 | 79 | 0.0 |
| TPVTTSTV | 717.07 | 79 | 0.0 |
| TQ.TQ.T.T | 717.07 | 79 | 0.0 |
| TQ.TTA.S | 717.07 | 79 | 0.0 |
| TQAPTT | 717.07 | 79 | 0.0 |
| TQESQT..S | 717.07 | 79 | 0.0 |
| TQQ.E.AE | 717.07 | 79 | 0.0 |
| TQS.ETT | 717.07 | 79 | 0.0 |
| TQSTT…S | 717.07 | 79 | 0.0 |
| TQTSE..SS | 717.07 | 79 | 0.0 |
| TRE.QT.T | 717.07 | 79 | 0.0 |
| TRFSS.PS | 717.07 | 79 | 0.0 |
| TRLVT..PS | 717.07 | 79 | 0.0 |
| TRSTA…P | 717.07 | 79 | 0.0 |
| TRSTQTT | 717.07 | 79 | 0.0 |
| TRV.Q..TL | 717.07 | 79 | 0.0 |
| TS.GAATR | 717.07 | 79 | 0.0 |
| TSA.TAT.S | 717.07 | 79 | 0.0 |
| TSASAST | 717.07 | 79 | 0.0 |
| TSASSQN | 717.07 | 79 | 0.0 |
| TSASTVSS | 717.07 | 79 | 0.0 |
| TSAVS.TA | 717.07 | 79 | 0.0 |
| TSE.ASS.S | 717.07 | 79 | 0.0 |
| TSE.IT.TT | 717.07 | 79 | 0.0 |
| TSF.SSVT | 717.07 | 79 | 0.0 |
| TSGETT..S | 717.07 | 79 | 0.0 |
| TSK.IT.TT | 717.07 | 79 | 0.0 |
| TSKVST | 717.07 | 79 | 0.0 |
| TSLSKTG | 717.07 | 79 | 0.0 |
| TSMMTSE | 717.07 | 79 | 0.0 |
| TSPDTA | 717.07 | 79 | 0.0 |
| TSPPPTTS | 717.07 | 79 | 0.0 |
| TSPQESP.V | 717.07 | 79 | 0.0 |
| TSPSGET | 717.07 | 79 | 0.0 |
| TSQ.ITTA | 717.07 | 79 | 0.0 |
| TSQTI.ST | 717.07 | 79 | 0.0 |
| TSRGS.T.S | 717.07 | 79 | 0.0 |
| TSSLCSV | 717.07 | 79 | 0.0 |
| TSSVP.T.S | 717.07 | 79 | 0.0 |
| TST.THRTT | 717.07 | 79 | 0.0 |
| TSTA.TES | 717.07 | 79 | 0.0 |
| TSTAG..SS | 717.07 | 79 | 0.0 |
| TSTPSFS.S | 717.07 | 79 | 0.0 |
| TSTSPQ.S | 717.07 | 79 | 0.0 |
| TT..KTTG | 717.07 | 79 | 0.0 |
| TT..PSSFS | 717.07 | 79 | 0.0 |
| TTAFS…Q | 717.07 | 79 | 0.0 |
| TTALIS..T | 717.07 | 79 | 0.0 |
| TTAPSTN.S | 717.07 | 79 | 0.0 |
| TTF.PAPT | 717.07 | 79 | 0.0 |
| TTG.A.LSS | 717.07 | 79 | 0.0 |
| TTGAG..T | 717.07 | 79 | 0.0 |
| TTGRP.E.S | 717.07 | 79 | 0.0 |
| TTPGSS.T | 717.07 | 79 | 0.0 |
| TTPSGSE | 717.07 | 79 | 0.0 |
| TTPSLKT | 717.07 | 79 | 0.0 |
| TTQAPTT | 717.07 | 79 | 0.0 |
| TTQES.TT | 717.07 | 79 | 0.0 |
| TTQSTT.L | 717.07 | 79 | 0.0 |
| TTR.S.TST | 717.07 | 79 | 0.0 |
| TTR.SS.PS | 717.07 | 79 | 0.0 |
| TTRVS…T | 717.07 | 79 | 0.0 |
| TTS.GTA.P | 717.07 | 79 | 0.0 |
| TTSFP.S.T | 717.07 | 79 | 0.0 |
| TTSQTI.ST | 717.07 | 79 | 0.0 |
| TTST.STL | 717.07 | 79 | 0.0 |
| TTSTDS.L | 717.07 | 79 | 0.0 |
| TTSTPSF | 717.07 | 79 | 0.0 |
| TTSWRTS | 717.07 | 79 | 0.0 |
| TTWPSS..S | 717.07 | 79 | 0.0 |
| TTWSQ.E.P | 717.07 | 79 | 0.0 |
| TTYSS.STT | 717.07 | 79 | 0.0 |
| TVPPTP.S | 717.07 | 79 | 0.0 |
| TVS.DSP | 717.07 | 79 | 0.0 |
| TVSQET..S | 717.07 | 79 | 0.0 |
| TVTTPGS | 717.07 | 79 | 0.0 |
| TWPSS..S | 717.07 | 79 | 0.0 |
| TWSQT.L.S | 717.07 | 79 | 0.0 |
| V.STPG.PE | 717.07 | 79 | 0.0 |
| V.TSTV.SS | 717.07 | 79 | 0.0 |
| VETTRVS | 717.07 | 79 | 0.0 |
| VPGTF..T | 717.07 | 79 | 0.0 |
| VPPTPPSI | 717.07 | 79 | 0.0 |
| VPQ.APT.S | 717.07 | 79 | 0.0 |
| VPV.GSL.P | 717.07 | 79 | 0.0 |
| VS.PG.PEG | 717.07 | 79 | 0.0 |
| VSAIG.PG | 717.07 | 79 | 0.0 |
| VSNTF…S | 717.07 | 79 | 0.0 |
| VSQINTL.T | 717.07 | 79 | 0.0 |
| VSS.TS.ST | 717.07 | 79 | 0.0 |
| VSSDSPLK | 717.07 | 79 | 0.0 |
| VST.MA.S | 717.07 | 79 | 0.0 |
| VSTIW.S | 717.07 | 79 | 0.0 |
| VT..VSITA | 717.07 | 79 | 0.0 |
| VT.TSM.TS | 717.07 | 79 | 0.0 |
| VTGSL.P.T | 717.07 | 79 | 0.0 |
| VTNTLM.TS | 717.07 | 79 | 0.0 |
| VTSKV…P | 717.07 | 79 | 0.0 |
| VTTPGS..T | 717.07 | 79 | 0.0 |
| W.TSI.DT | 717.07 | 79 | 0.0 |
| WPSSFS.K | 717.07 | 79 | 0.0 |
| WSDTLS.A | 717.07 | 79 | 0.0 |
| WSQ.E.PS | 717.07 | 79 | 0.0 |
| WT..T.TT | 717.07 | 79 | 0.0 |
| WTS.SAST | 717.07 | 79 | 0.0 |
| WWS.T.STA | 717.07 | 79 | 0.0 |
| YS.HSTT.P | 717.07 | 79 | 0.0 |
| T.T.MMTS | 714.05 | 79 | 0.0 |
| ASS.NH.T | 712.54 | 79 | 0.0 |
| D.SASSQ.H | 712.54 | 79 | 0.0 |
| H.T.S.ETT | 712.54 | 79 | 0.0 |
| Q.TAA.SR | 712.54 | 79 | 0.0 |
| QNH.T.ST | 712.54 | 79 | 0.0 |
| RTS.Q..SA | 712.54 | 79 | 0.0 |
| S.NHQT.S | 712.54 | 79 | 0.0 |
| S.RTS.QD | 712.54 | 79 | 0.0 |
| SASSQNH.T | 712.54 | 79 | 0.0 |
| SPSVH.V | 712.54 | 79 | 0.0 |
| SPSVHN | 712.54 | 79 | 0.0 |
| SSQ.H.T.S | 712.54 | 79 | 0.0 |
| TSTGST | 712.54 | 79 | 0.0 |
| APVTST.ST | 708.00 | 78 | 0.0 |
| AQT.T.TQ | 708.00 | 78 | 0.0 |
| ATL.G.STA | 708.00 | 78 | 0.0 |
| DT.TQM.TS | 708.00 | 78 | 0.0 |
| DTL.T.S.T | 708.00 | 78 | 0.0 |
| EDTLIT | 708.00 | 78 | 0.0 |
| ETTSKA.T | 708.00 | 78 | 0.0 |
| FSSPS…V | 708.00 | 78 | 0.0 |
| GSTTAT..G | 708.00 | 78 | 0.0 |
| ISASSQ..Q | 708.00 | 78 | 0.0 |
| ITGSK…P | 708.00 | 78 | 0.0 |
| KAQTDT.T | 708.00 | 78 | 0.0 |
| KSTETTS | 708.00 | 78 | 0.0 |
| KT.AP.TST | 708.00 | 78 | 0.0 |
| LFSSPSV | 708.00 | 78 | 0.0 |
| MMTST..SS | 708.00 | 78 | 0.0 |
| MTSTLF..P | 708.00 | 78 | 0.0 |
| N…SASSQ | 708.00 | 78 | 0.0 |
| NH.TKST.T | 708.00 | 78 | 0.0 |
| PSV.NV.ET | 708.00 | 78 | 0.0 |
| Q.ISA.S.N | 708.00 | 78 | 0.0 |
| Q.MTST..S | 708.00 | 78 | 0.0 |
| QT.ST.TTS | 708.00 | 78 | 0.0 |
| QTDTLT | 708.00 | 78 | 0.0 |
| SKAQT.TLT | 708.00 | 78 | 0.0 |
| SKT.AP.TS | 708.00 | 78 | 0.0 |
| SN..ISASS | 708.00 | 78 | 0.0 |
| SRT.NQ..S | 708.00 | 78 | 0.0 |
| SSRTSNQ | 708.00 | 78 | 0.0 |
| STETTS | 708.00 | 78 | 0.0 |
| STGSTT.T | 708.00 | 78 | 0.0 |
| STLFSSP | 708.00 | 78 | 0.0 |
| STTATLE | 708.00 | 78 | 0.0 |
| T.EGQS.A | 708.00 | 78 | 0.0 |
| TATLEG.S | 708.00 | 78 | 0.0 |
| TDTLTQ..T | 708.00 | 78 | 0.0 |
| TEDTL..GS | 708.00 | 78 | 0.0 |
| TETTS…T | 708.00 | 78 | 0.0 |
| TGSTT.T | 708.00 | 78 | 0.0 |
| TKSTETT | 708.00 | 78 | 0.0 |
| TL.TGS.T | 708.00 | 78 | 0.0 |
| TLFSSPS | 708.00 | 78 | 0.0 |
| TQM.TSTL | 708.00 | 78 | 0.0 |
| TSK.QT.T | 708.00 | 78 | 0.0 |
| TSNQDI.A | 708.00 | 78 | 0.0 |
| TST.F.S.S | 708.00 | 78 | 0.0 |
| TSTGSTT.T | 708.00 | 78 | 0.0 |
| TTA.LE.QS | 708.00 | 78 | 0.0 |
| TTE.TL.TG | 708.00 | 78 | 0.0 |
| TTSKA.T.T | 708.00 | 78 | 0.0 |
| AAP.TST.S | 617.23 | 68 | 0.0 |
| DPE.QS.A | 617.23 | 68 | 0.0 |
| EG.SPAT.S | 617.23 | 68 | 0.0 |
| GQS.ATFS | 617.23 | 68 | 0.0 |
| GS.TA.PVT | 617.23 | 68 | 0.0 |
| LIT.S.TAA | 617.23 | 68 | 0.0 |
| PAT.S.TST | 617.23 | 68 | 0.0 |
| QSPAT…T | 617.23 | 68 | 0.0 |
| TGSKTAA | 617.23 | 68 | 0.0 |
| ATGFP..SS | 571.84 | 63 | 0.0 |
| DLE.V.RT | 571.84 | 63 | 0.0 |
| EFVRRTV | 571.84 | 63 | 0.0 |
| ERGVS…Y | 571.84 | 63 | 0.0 |
| FKP.TG.PL | 571.84 | 63 | 0.0 |
| FPLGSS.R | 571.84 | 63 | 0.0 |
| FTSPL..PA | 571.84 | 63 | 0.0 |
| G.SLFP.GA | 571.84 | 63 | 0.0 |
| GDL.FVR.T | 571.84 | 63 | 0.0 |
| GF..GSS.R | 571.84 | 63 | 0.0 |
| ILPERG..L | 571.84 | 63 | 0.0 |
| K.ATGFP | 571.84 | 63 | 0.0 |
| LE.VR.TV | 571.84 | 63 | 0.0 |
| LFKPA..FP | 571.84 | 63 | 0.0 |
| LGSSL..SL | 571.84 | 63 | 0.0 |
| LPERGVS | 571.84 | 63 | 0.0 |
| PATGFP.G | 571.84 | 63 | 0.0 |
| PERGVS..P | 571.84 | 63 | 0.0 |
| PL..PATG | 571.84 | 63 | 0.0 |
| PLGSSL..S | 571.84 | 63 | 0.0 |
| R..VDF.SP | 571.84 | 63 | 0.0 |
| RGVSLF..G | 571.84 | 63 | 0.0 |
| RTV.FTS | 571.84 | 63 | 0.0 |
| SPLFK..T | 571.84 | 63 | 0.0 |
| SS.RDSLY | 571.84 | 63 | 0.0 |
| TGF.L.SSL | 571.84 | 63 | 0.0 |
| TSP.FK..T | 571.84 | 63 | 0.0 |
| TVDFTS | 571.84 | 63 | 0.0 |
| VD.TSP..K | 571.84 | 63 | 0.0 |
| VRRTV..TS | 571.84 | 63 | 0.0 |
| T.MMTS | 535.54 | 79 | 0.0 |
| G..SS.PVT | 478.05 | 79 | 0.0 |
| LT..TTST | 478.05 | 79 | 0.0 |
| NTEETS | 478.05 | 79 | 0.0 |
| S..PVTSA | 478.05 | 79 | 0.0 |
| S.QDT.A.S | 478.05 | 79 | 0.0 |
| N.E.AN.TL | 453.84 | 25 | 0.0 |
| PISTTT | 435.69 | 4 | 0.0 |
| P.TST.STT | 363.08 | 80 | 0.0 |
| TS.QDT.A | 358.54 | 79 | 0.0 |
| ADFST…T | 354.00 | 39 | 0.0 |
| AL.LY.SG | 354.00 | 39 | 0.0 |
| ALVAP.W | 354.00 | 39 | 0.0 |
| APFWD | 354.00 | 39 | 0.0 |
| DDA.F..GR | 354.00 | 39 | 0.0 |
| DFSTG..TT | 354.00 | 39 | 0.0 |
| DPV.LV.P | 354.00 | 39 | 0.0 |
| DSL.FTD | 354.00 | 39 | 0.0 |
| DYQ.F…N | 354.00 | 39 | 0.0 |
| ESD.Q..SY | 354.00 | 39 | 0.0 |
| F..QSGG.Q | 354.00 | 39 | 0.0 |
| F.DDA.FS | 354.00 | 39 | 0.0 |
| FP.SD.Q.F | 354.00 | 39 | 0.0 |
| FSTGR.T | 354.00 | 39 | 0.0 |
| FT.NG…F | 354.00 | 39 | 0.0 |
| FTGRDPV | 354.00 | 39 | 0.0 |
| GQIIFP | 354.00 | 39 | 0.0 |
| I.STDGSR | 354.00 | 39 | 0.0 |
| L.QSGG.Q | 354.00 | 39 | 0.0 |
| LF.YQSG | 354.00 | 39 | 0.0 |
| LRD.LY.TD | 354.00 | 39 | 0.0 |
| LSTDGSR | 354.00 | 39 | 0.0 |
| LV.PF.D.A | 354.00 | 39 | 0.0 |
| LYF.D.G.I | 354.00 | 39 | 0.0 |
| MQ.DV.Q | 354.00 | 39 | 0.0 |
| NG..IFP.S | 354.00 | 39 | 0.0 |
| NTY.A.LST | 354.00 | 39 | 0.0 |
| PFW.DA | 354.00 | 39 | 0.0 |
| PVALV.P | 354.00 | 39 | 0.0 |
| QIFSYP | 354.00 | 39 | 0.0 |
| QSGGM | 354.00 | 39 | 0.0 |
| RDS.Y.TD | 354.00 | 39 | 0.0 |
| SNT.QA..S | 354.00 | 39 | 0.0 |
| STG.GTTF | 354.00 | 39 | 0.0 |
| SY..FLYQ | 354.00 | 39 | 0.0 |
| TDGSRS | 354.00 | 39 | 0.0 |
| TDNGQ…P | 354.00 | 39 | 0.0 |
| TGFTG..P | 354.00 | 39 | 0.0 |
| TGR.TT.Y | 354.00 | 39 | 0.0 |
| TYQAI..T | 354.00 | 39 | 0.0 |
| V.PFW.D.D | 354.00 | 39 | 0.0 |
| VAL.AP..D | 354.00 | 39 | 0.0 |
| W..ADFST | 354.00 | 39 | 0.0 |
| YFT.NG..I | 354.00 | 39 | 0.0 |
| YQ.FS.P.P | 354.00 | 39 | 0.0 |
| YQAILS | 354.00 | 39 | 0.0 |
| T…DTPEV | 335.84 | 7 | 0.0 |
| D.DYRN | 326.77 | 7 | 0.0 |
| DPH.TT | 326.77 | 28 | 0.0 |
| TTP.P…T | 323.38 | 106 | 0.0 |
| TP.P.T | 293.10 | 131 | 0.0 |
| FV..T..FT | 285.92 | 63 | 0.0 |
| D..RV..DN | 281.38 | 31 | 0.0 |
| Y..ECL.W | 281.38 | 31 | 0.0 |
| EK.KGH..G | 263.23 | 7 | 0.0 |
| VPGVG..G | 263.23 | 15 | 0.0 |
| G..GVPGVG | 254.15 | 15 | 0.0 |
| PT.NRE.T | 254.15 | 2 | 0.0 |
| T.NRE.TA | 254.15 | 2 | 0.0 |
| E..ER.ANE | 236.00 | 2 | 0.0 |
| Q..PH.G.A | 236.00 | 26 | 0.0 |
| A.SWI.K.T | 226.92 | 25 | 0.0 |
| AT..QYPP | 226.92 | 25 | 0.0 |
| ATNFI | 226.92 | 25 | 0.0 |
| AY..QWT | 226.92 | 25 | 0.0 |
| C..SWQ..R | 226.92 | 25 | 0.0 |
| C.T.RPPQ | 226.92 | 25 | 0.0 |
| C.W.DK.Y | 226.92 | 25 | 0.0 |
| CA..Q.RR | 226.92 | 25 | 0.0 |
| D.RMP..ST | 226.92 | 25 | 0.0 |
| DFRMP | 226.92 | 25 | 0.0 |
| DG.GQ.TF | 226.92 | 25 | 0.0 |
| DGD.SC.Y | 226.92 | 25 | 0.0 |
| DGV.YT..G | 226.92 | 25 | 0.0 |
| DN.TVT..P | 226.92 | 25 | 0.0 |
| DT..L.NA | 226.92 | 25 | 0.0 |
| E..PLMS.P | 226.92 | 25 | 0.0 |
| E.M.FH.G | 226.92 | 25 | 0.0 |
| EAYKG | 226.92 | 25 | 0.0 |
| ED..GQ.TF | 226.92 | 25 | 0.0 |
| EY.TF..EH | 226.92 | 25 | 0.0 |
| F.P.S.G.W | 226.92 | 25 | 0.0 |
| FG.PH.T | 226.92 | 25 | 0.0 |
| FH..MT.Q | 226.92 | 25 | 0.0 |
| FNG.GD | 226.92 | 25 | 0.0 |
| FTP.FY | 226.92 | 25 | 0.0 |
| G…FLLQG | 226.92 | 25 | 0.0 |
| G..IPPG.P | 226.92 | 25 | 0.0 |
| G..QLCS.T | 226.92 | 25 | 0.0 |
| G..SRQL.S | 226.92 | 25 | 0.0 |
| G.G.LGK.N | 226.92 | 25 | 0.0 |
| G.TTLI.Y | 226.92 | 25 | 0.0 |
| G.WGLGS | 226.92 | 25 | 0.0 |
| GCATY.P | 226.92 | 25 | 0.0 |
| GDSSCI | 226.92 | 25 | 0.0 |
| GFSSGDG | 226.92 | 25 | 0.0 |
| GG..C.Y.P | 226.92 | 25 | 0.0 |
| GLG.F.L.G | 226.92 | 25 | 0.0 |
| GLLGVW | 226.92 | 25 | 0.0 |
| GP.TVQW | 226.92 | 25 | 0.0 |
| GRT.QT.S | 226.92 | 25 | 0.0 |
| GS.TYQ..L | 226.92 | 25 | 0.0 |
| GSA.ATN | 226.92 | 25 | 0.0 |
| GTT.YQE | 226.92 | 25 | 0.0 |
| GVS.TF..L | 226.92 | 25 | 0.0 |
| H..AS.PPE | 226.92 | 25 | 0.0 |
| H..ER.N.R | 226.92 | 25 | 0.0 |
| HITTL…S | 226.92 | 25 | 0.0 |
| HTREVS | 226.92 | 25 | 0.0 |
| IEA.K..TT | 226.92 | 25 | 0.0 |
| IS.CDGD | 226.92 | 25 | 0.0 |
| L.Q.LEPQ | 226.92 | 25 | 0.0 |
| L.TREVS | 226.92 | 25 | 0.0 |
| LD.VSYT | 226.92 | 25 | 0.0 |
| LQ.RT.QT | 226.92 | 25 | 0.0 |
| LS..LH.SA | 226.92 | 25 | 0.0 |
| LVQQA..W | 226.92 | 25 | 0.0 |
| MGFS..DG | 226.92 | 25 | 0.0 |
| MPN.ST..P | 226.92 | 25 | 0.0 |
| MS.PV…Y | 226.92 | 25 | 0.0 |
| MTWQ..G | 226.92 | 25 | 0.0 |
| N..SC.C.W | 226.92 | 25 | 0.0 |
| NATGV…R | 226.92 | 25 | 0.0 |
| NCDGD | 226.92 | 25 | 0.0 |
| ND.PY..A | 226.92 | 25 | 0.0 |
| NFT.VF.S | 226.92 | 25 | 0.0 |
| NRT.GL.G | 226.92 | 25 | 0.0 |
| P.DDF.MP | 226.92 | 25 | 0.0 |
| P.G.F.E.W | 226.92 | 25 | 0.0 |
| P.N.TP.F | 226.92 | 25 | 0.0 |
| P.W.W..VS | 226.92 | 25 | 0.0 |
| PH.TT..GV | 226.92 | 25 | 0.0 |
| PLMS.PV | 226.92 | 25 | 0.0 |
| PNY.LEC | 226.92 | 25 | 0.0 |
| PS.NGG..I | 226.92 | 25 | 0.0 |
| PY.CA…Q | 226.92 | 25 | 0.0 |
| Q.CSFTS | 226.92 | 25 | 0.0 |
| Q.LEPQS | 226.92 | 25 | 0.0 |
| Q.T.I.YT | 226.92 | 25 | 0.0 |
| Q.TNFI | 226.92 | 25 | 0.0 |
| Q.W.C..ND | 226.92 | 25 | 0.0 |
| QK.SSW | 226.92 | 25 | 0.0 |
| QLAQE.E.Q | 226.92 | 25 | 0.0 |
| QR.PH..C | 226.92 | 25 | 0.0 |
| QRPWQ | 226.92 | 25 | 0.0 |
| QTGSA..T | 226.92 | 25 | 0.0 |
| QYPPS…G | 226.92 | 25 | 0.0 |
| REVSK..E | 226.92 | 25 | 0.0 |
| RGGVC..Y | 226.92 | 25 | 0.0 |
| RGTTF…Y | 226.92 | 25 | 0.0 |
| RMP.GST | 226.92 | 25 | 0.0 |
| RN..EVS.S | 226.92 | 25 | 0.0 |
| RPDRFL | 226.92 | 25 | 0.0 |
| RPNY…C | 226.92 | 25 | 0.0 |
| RQ.C.FT | 226.92 | 25 | 0.0 |
| RSS.L.P.T | 226.92 | 25 | 0.0 |
| S.G.NQ..C | 226.92 | 25 | 0.0 |
| S.IPP..PE | 226.92 | 25 | 0.0 |
| SF..W..VS | 226.92 | 25 | 0.0 |
| SIG.WG | 226.92 | 25 | 0.0 |
| SIGR.G.G | 226.92 | 25 | 0.0 |
| SIN.G.V.E | 226.92 | 25 | 0.0 |
| SN.TP.F.S | 226.92 | 25 | 0.0 |
| SNCDG…C | 226.92 | 25 | 0.0 |
| SNI.H.S.S | 226.92 | 25 | 0.0 |
| SSCIY.T | 226.92 | 25 | 0.0 |
| SSGDGY | 226.92 | 25 | 0.0 |
| SW.GG..C | 226.92 | 25 | 0.0 |
| SWAEH..S | 226.92 | 25 | 0.0 |
| SWCCR | 226.92 | 25 | 0.0 |
| T.D.VS.TF | 226.92 | 25 | 0.0 |
| T.W.GG.C | 226.92 | 25 | 0.0 |
| TF.GLG.F | 226.92 | 25 | 0.0 |
| TFYGE.S | 226.92 | 25 | 0.0 |
| TREVSK | 226.92 | 25 | 0.0 |
| TTL.QY.S | 226.92 | 25 | 0.0 |
| VSCPCS | 226.92 | 25 | 0.0 |
| VT.I.DC | 226.92 | 25 | 0.0 |
| VW..YR.D | 226.92 | 25 | 0.0 |
| W..GVCC | 226.92 | 25 | 0.0 |
| W..VS.PCS | 226.92 | 25 | 0.0 |
| WA.KVT..N | 226.92 | 25 | 0.0 |
| WE..RPD | 226.92 | 25 | 0.0 |
| WG..Q.SC | 226.92 | 25 | 0.0 |
| WGEFR.G | 226.92 | 25 | 0.0 |
| WTL.SNT | 226.92 | 25 | 0.0 |
| Y.P.INGG | 226.92 | 25 | 0.0 |
| YE..Y.EH | 226.92 | 25 | 0.0 |
| YF..S..MS | 226.92 | 25 | 0.0 |
| YK.QTT | 226.92 | 25 | 0.0 |
| YP..WT..S | 226.92 | 25 | 0.0 |
| YRP.RFL | 226.92 | 25 | 0.0 |
| YTFNGL | 226.92 | 25 | 0.0 |
| CLYN.T | 217.85 | 24 | 0.0 |
| W..VS.T.A | 217.85 | 6 | 0.0 |
| FE.GT..W | 208.77 | 23 | 0.0 |
| G.CY..QT | 208.77 | 23 | 0.0 |
| GVPG.GV | 208.77 | 16 | 0.0 |
| N…LW.PK | 208.77 | 23 | 0.0 |
| NQT.R.G.S | 208.77 | 23 | 0.0 |
| PKSLEP | 208.77 | 23 | 0.0 |
| Q.RT..CH | 208.77 | 23 | 0.0 |
| S..PFTL.I | 208.77 | 23 | 0.0 |
| SQCLY | 208.77 | 23 | 0.0 |
| TLLWTP | 208.77 | 23 | 0.0 |
| TSRVGN | 208.77 | 23 | 0.0 |
| WTPKS..P | 208.77 | 23 | 0.0 |
| VT.TS | 207.41 | 115 | 0.0 |
| APGVGVAPG | 204.23 | 15 | 0.0 |
| G.GIPG..V | 204.23 | 15 | 0.0 |
| GLGVG.GV | 204.23 | 15 | 0.0 |
| PGLGVG.G | 204.23 | 15 | 0.0 |
| VG.GVPG.G | 204.23 | 15 | 0.0 |
| DTPEV..A | 199.69 | 7 | 0.0 |
| LKFT..VD | 199.69 | 7 | 0.0 |
| PGTTE.T | 199.69 | 22 | 0.0 |
| RV..A.KGQ | 199.69 | 44 | 0.0 |
| S.VDTP..V | 199.69 | 7 | 0.0 |
| S.VDTPE.I | 199.69 | 7 | 0.0 |
| VI.AKKA | 199.69 | 7 | 0.0 |
| WIR.MT | 199.69 | 22 | 0.0 |
| K.GPAG..G | 195.15 | 24 | 0.0 |
| D.P.K.ETS | 190.61 | 21 | 0.0 |
| G..GA.GPH | 190.61 | 21 | 0.0 |
| G..GRPG.T | 190.61 | 21 | 0.0 |
| GK.CGR | 190.61 | 21 | 0.0 |
| GVPGVGG | 190.61 | 15 | 0.0 |
| HTT.LP.S | 190.61 | 21 | 0.0 |
| K.G.DG.RG | 190.61 | 21 | 0.0 |
| TQF.Y.D | 190.61 | 21 | 0.0 |
| G..TTFY | 186.08 | 41 | 0.0 |
| P.T.TS | 183.08 | 120 | 0.0 |
| ARW.RV | 181.54 | 20 | 0.0 |
| C.LPH..P | 181.54 | 20 | 0.0 |
| G.PG.GVG | 181.54 | 15 | 0.0 |
| GA.VPGV | 181.54 | 15 | 0.0 |
| GPIGL.G.P | 181.54 | 36 | 0.0 |
| M…RW.RV | 181.54 | 20 | 0.0 |
| P.RTCQ | 181.54 | 20 | 0.0 |
| V.WVS..C | 181.54 | 20 | 0.0 |
| VS.SC.C | 181.54 | 20 | 0.0 |
| W.W.L.S.T | 181.54 | 40 | 0.0 |
| QW..AQ.S | 177.00 | 39 | 0.0 |
| WD.AQ.S | 177.00 | 39 | 0.0 |
| T.TS…T | 176.72 | 111 | 0.0 |
| R..LH.W.C | 175.49 | 7 | 0.0 |
| A.G.SG.KG | 172.46 | 19 | 0.0 |
| AP.VDVL | 172.46 | 19 | 0.0 |
| C.P.LPDG | 172.46 | 19 | 0.0 |
| CQ.QQG | 172.46 | 19 | 0.0 |
| DCKK.VT | 172.46 | 19 | 0.0 |
| DL.APPR | 172.46 | 19 | 0.0 |
| DQ.GRP.P | 172.46 | 19 | 0.0 |
| E..TGRP.P | 172.46 | 19 | 0.0 |
| EL.D..YW | 172.46 | 19 | 0.0 |
| EPA.LE..M | 172.46 | 19 | 0.0 |
| F.DGC..Q | 172.46 | 19 | 0.0 |
| FG..GG.KG | 172.46 | 19 | 0.0 |
| FKG.IG..G | 172.46 | 19 | 0.0 |
| G…AD.KW | 172.46 | 19 | 0.0 |
| G..GMPGD | 172.46 | 19 | 0.0 |
| G..GPHGE | 172.46 | 19 | 0.0 |
| G.AGS.G.I | 172.46 | 19 | 0.0 |
| G.AGVG.P | 172.46 | 19 | 0.0 |
| G.GGPIG | 172.46 | 19 | 0.0 |
| G.QGG.G.K | 172.46 | 19 | 0.0 |
| G.RGFNG | 172.46 | 19 | 0.0 |
| G.T.LEV.T | 172.46 | 19 | 0.0 |
| GAKGT.G | 172.46 | 19 | 0.0 |
| GG.KGP.V | 172.46 | 19 | 0.0 |
| GPKGSLG | 172.46 | 19 | 0.0 |
| GT.GG.G.H | 172.46 | 19 | 0.0 |
| GTKGN.G | 172.46 | 19 | 0.0 |
| GVDG.RG | 172.46 | 19 | 0.0 |
| H.DVS..C | 172.46 | 19 | 0.0 |
| HGPRG..G | 172.46 | 19 | 0.0 |
| ICP.D..Y | 172.46 | 19 | 0.0 |
| IG.GGP.G | 172.46 | 19 | 0.0 |
| IVPGVQ | 172.46 | 19 | 0.0 |
| K.CHP..P | 172.46 | 19 | 0.0 |
| KDFSL.T | 172.46 | 19 | 0.0 |
| KTR.SV..S | 172.46 | 19 | 0.0 |
| KW.RV..A | 172.46 | 19 | 0.0 |
| L.IVPG..A | 172.46 | 19 | 0.0 |
| LAL.G.PGP | 172.46 | 19 | 0.0 |
| LMQE.EA | 172.46 | 19 | 0.0 |
| N.GPSGP.G | 172.46 | 19 | 0.0 |
| N.QPHR | 172.46 | 19 | 0.0 |
| P.LPDGV | 172.46 | 19 | 0.0 |
| P.RFGS.G | 172.46 | 19 | 0.0 |
| PG.MGY.G | 172.46 | 19 | 0.0 |
| PG.SGPIG | 172.46 | 19 | 0.0 |
| PGVQA.Y | 172.46 | 19 | 0.0 |
| PTGTQ..P | 172.46 | 19 | 0.0 |
| PVCFM | 172.46 | 19 | 0.0 |
| QGA.G.TG | 172.46 | 19 | 0.0 |
| RAQRS..Q | 172.46 | 19 | 0.0 |
| RG.SGK.G | 172.46 | 19 | 0.0 |
| RPQN..P | 172.46 | 19 | 0.0 |
| RT..LEQ.P | 172.46 | 19 | 0.0 |
| S.SPI..W | 172.46 | 19 | 0.0 |
| SVSAH..V | 172.46 | 19 | 0.0 |
| T.G.NG.PG | 172.46 | 19 | 0.0 |
| T.I.DC..R | 172.46 | 19 | 0.0 |
| TCQ.L…H | 172.46 | 19 | 0.0 |
| TG.NG.PG | 172.46 | 19 | 0.0 |
| TGR.QP.S | 172.46 | 19 | 0.0 |
| TR..P.SAR | 172.46 | 19 | 0.0 |
| TR.SVD.S | 172.46 | 19 | 0.0 |
| TSLML…F | 172.46 | 19 | 0.0 |
| VDG.RG..G | 172.46 | 19 | 0.0 |
| VL..RTPV | 172.46 | 19 | 0.0 |
| T..TTP | 169.24 | 115 | 0.0 |
| GG..IE.Y | 167.92 | 31 | 0.0 |
| DQ.DVI..R | 166.41 | 7 | 0.0 |
| PGW.G..PV | 163.38 | 18 | 0.0 |
| SD.LYKS | 163.38 | 7 | 0.0 |
| W…P.VDV | 163.38 | 18 | 0.0 |
| YEK.KGH | 163.38 | 7 | 0.0 |
| ST..TT | 160.63 | 126 | 0.0 |
| P..DYQ..S | 160.36 | 53 | 0.0 |
| S…T..TT | 158.72 | 127 | 0.0 |
| V.D.DY.NY | 156.58 | 7 | 0.0 |
| AG.DG.KG | 154.31 | 34 | 0.0 |
| PIKKP..P | 154.31 | 15 | 0.0 |
| PP.REGP | 154.31 | 17 | 0.0 |
| QNDVI…K | 154.31 | 7 | 0.0 |
| R.G.G.GDK | 154.31 | 34 | 0.0 |
| SD..YR.Y | 154.31 | 7 | 0.0 |
| TS.VDT.E | 154.31 | 7 | 0.0 |
| DL..LKG.G | 151.28 | 7 | 0.0 |
| CD..KGY | 145.23 | 16 | 0.0 |
| CD.GT.G | 145.23 | 16 | 0.0 |
| CDHG..C | 145.23 | 16 | 0.0 |
| D.G.FG..C | 145.23 | 16 | 0.0 |
| F.C.G.KG | 145.23 | 16 | 0.0 |
| F.C.N..CP | 145.23 | 16 | 0.0 |
| FG.YCE | 145.23 | 16 | 0.0 |
| FP..HC..G | 145.23 | 16 | 0.0 |
| GC.CD..T | 145.23 | 16 | 0.0 |
| GK.CE..P | 145.23 | 16 | 0.0 |
| GTRGF.G | 145.23 | 32 | 0.0 |
| K.C.A.P.N | 145.23 | 16 | 0.0 |
| K.YF..DG | 145.23 | 16 | 0.0 |
| PCF..VH | 145.23 | 16 | 0.0 |
| Q.G.TC..P | 145.23 | 16 | 0.0 |
| QSG.TC | 145.23 | 16 | 0.0 |
| QSGFT | 145.23 | 16 | 0.0 |
| R.D.YKG | 145.23 | 16 | 0.0 |
| RC.CVS | 145.23 | 16 | 0.0 |
| S…GCQ.M | 145.23 | 16 | 0.0 |
| SEF.YR | 145.23 | 16 | 0.0 |
| WM..SE..Y | 145.23 | 16 | 0.0 |
| YDL.Y.P | 145.23 | 16 | 0.0 |
| YTAWG | 145.23 | 16 | 0.0 |
| S..VAKIQ | 142.20 | 21 | 0.0 |
| DDPK..W | 140.69 | 7 | 0.0 |
| PVT..WL | 140.69 | 31 | 0.0 |
| S.G.VT..W | 140.69 | 31 | 0.0 |
| Y.EYE..Y | 140.69 | 31 | 0.0 |
| P.TPEM..V | 139.18 | 7 | 0.0 |
| GV.PGVG | 138.42 | 15 | 0.0 |
| PDQ.D.I.A | 137.67 | 7 | 0.0 |
| A.PGVG..G | 136.15 | 15 | 0.0 |
| AAAAAAKAA | 136.15 | 15 | 0.0 |
| AGLGA.I | 136.15 | 15 | 0.0 |
| AKYGA..G | 136.15 | 15 | 0.0 |
| DPKLV.A | 136.15 | 5 | 0.0 |
| G..GGVF.P | 136.15 | 15 | 0.0 |
| G.PGFG..V | 136.15 | 15 | 0.0 |
| GKAGY | 136.15 | 15 | 0.0 |
| GV.PGVG.A | 136.15 | 15 | 0.0 |
| GV.PGVGV | 136.15 | 15 | 0.0 |
| GVG.APG.G | 136.15 | 15 | 0.0 |
| GVPG.I.G | 136.15 | 15 | 0.0 |
| H…PGGVP | 136.15 | 15 | 0.0 |
| LYK.AY | 136.15 | 5 | 0.0 |
| P.ENR..TA | 136.15 | 2 | 0.0 |
| PG.GVA.GV | 136.15 | 15 | 0.0 |
| RA..IE.KH | 136.15 | 5 | 0.0 |
| TPEML.A | 136.15 | 5 | 0.0 |
| V.PGVG.AP | 136.15 | 15 | 0.0 |
| VGG.G..PP | 136.15 | 15 | 0.0 |
| VK.NQE..S | 136.15 | 7 | 0.0 |
| VPG.IPG | 136.15 | 15 | 0.0 |
| Y..QSDS.Y | 136.15 | 5 | 0.0 |
| PG.GV..GV | 133.56 | 15 | 0.0 |
| PGVGV.P | 132.52 | 15 | 0.0 |
| E..TL.WTP | 131.61 | 29 | 0.0 |
| IV.CK…T | 131.61 | 29 | 0.0 |
| N..IQA.KA | 131.61 | 7 | 0.0 |
| N.TL.WT | 131.61 | 29 | 0.0 |
| G..GK.GPA | 130.10 | 24 | 0.0 |
| G.VPGVG | 129.35 | 15 | 0.0 |
| A…I.KPW | 127.08 | 14 | 0.0 |
| C.PDQ.D | 127.08 | 7 | 0.0 |
| E..KTK.H | 127.08 | 7 | 0.0 |
| EQP.PPG | 127.08 | 14 | 0.0 |
| F…RGG.C | 127.08 | 28 | 0.0 |
| F.P.R..MP | 127.08 | 14 | 0.0 |
| FPAVT.P | 127.08 | 14 | 0.0 |
| G.CG.YP | 127.08 | 14 | 0.0 |
| GEM.D..G | 127.08 | 14 | 0.0 |
| GF.PSG.P | 127.08 | 14 | 0.0 |
| GTYGG.G | 127.08 | 14 | 0.0 |
| GVG.GGI | 127.08 | 15 | 0.0 |
| K..SER.Y | 127.08 | 7 | 0.0 |
| K..WP.PK | 127.08 | 14 | 0.0 |
| NG.T.PPG | 127.08 | 28 | 0.0 |
| P..CP.WS | 127.08 | 14 | 0.0 |
| P..LPWPP | 127.08 | 14 | 0.0 |
| P.YLP.PP | 127.08 | 14 | 0.0 |
| PKRPP..P | 127.08 | 14 | 0.0 |
| PMPP.N | 127.08 | 14 | 0.0 |
| PPQVC | 127.08 | 14 | 0.0 |
| PRW.M..P | 127.08 | 14 | 0.0 |
| Q..VEM.I | 127.08 | 14 | 0.0 |
| QPK.PP..P | 127.08 | 14 | 0.0 |
| RP..M.PM | 127.08 | 14 | 0.0 |
| RS.SG.NS | 127.08 | 14 | 0.0 |
| T.TSYH | 127.08 | 7 | 0.0 |
| TAW.ET | 127.08 | 14 | 0.0 |
| VGVGG.P | 127.08 | 15 | 0.0 |
| Y.PQGP | 127.08 | 14 | 0.0 |
| TC.PDQ.D | 125.26 | 7 | 0.0 |
| E..SSV.YK | 124.81 | 7 | 0.0 |
| ER.K.NQE | 124.81 | 7 | 0.0 |
| H..TCLPD | 124.81 | 7 | 0.0 |
| WTCLPD | 124.81 | 7 | 0.0 |
| AAAKAA..G | 124.48 | 15 | 0.0 |
| AAAAKAA | 124.05 | 15 | 0.0 |
| GAGVKP | 122.54 | 14 | 0.0 |
| Q.RSSSL | 122.54 | 27 | 0.0 |
| AAAAAKAA | 121.63 | 15 | 0.0 |
| PK.MW..H | 121.03 | 7 | 0.0 |
| SM.VAK | 121.03 | 7 | 0.0 |
| H.W.C.PD | 119.13 | 7 | 0.0 |
| LH.W.C.P | 119.13 | 7 | 0.0 |
| DLEWL.G.G | 118.00 | 7 | 0.0 |
| G.PGFG.V | 118.00 | 13 | 0.0 |
| GIPGVG | 118.00 | 13 | 0.0 |
| I.A.KAY.L | 118.00 | 7 | 0.0 |
| K.DLEW..G | 118.00 | 7 | 0.0 |
| P.GPTG.D | 118.00 | 20 | 0.0 |
| PDK.KFT | 118.00 | 7 | 0.0 |
| PQSW.C | 118.00 | 26 | 0.0 |
| YA..LYQ | 118.00 | 39 | 0.0 |
| YK.D.EW | 118.00 | 7 | 0.0 |
| YK.DLEW | 118.00 | 7 | 0.0 |
| AGTAT.S.S | 115.73 | 87 | 0.0 |
| RPQNQ | 114.97 | 19 | 0.0 |
| A.W.LK.T | 113.46 | 25 | 0.0 |
| CPCS…G | 113.46 | 25 | 0.0 |
| D..W.KGI | 113.46 | 7 | 0.0 |
| G..GN.GPV | 113.46 | 31 | 0.0 |
| GDG.F.N | 113.46 | 25 | 0.0 |
| GMPGD.G | 113.46 | 25 | 0.0 |
| GQ.TF…G | 113.46 | 25 | 0.0 |
| HV.CAT | 113.46 | 25 | 0.0 |
| IGR.G.GS | 113.46 | 25 | 0.0 |
| KG.EG.SG | 113.46 | 25 | 0.0 |
| LRDSCT | 113.46 | 25 | 0.0 |
| LRF.PVS | 113.46 | 25 | 0.0 |
| ML..FG..W | 113.46 | 25 | 0.0 |
| MTW.I..T | 113.46 | 25 | 0.0 |
| NCD.D..C | 113.46 | 25 | 0.0 |
| NS..MS.P | 113.46 | 25 | 0.0 |
| PCSWQ | 113.46 | 25 | 0.0 |
| PG.VI..LP | 113.46 | 25 | 0.0 |
| PHDA.R | 113.46 | 25 | 0.0 |
| PPGEV..P | 113.46 | 25 | 0.0 |
| QGKTG.PG | 113.46 | 25 | 0.0 |
| QP.WE…P | 113.46 | 25 | 0.0 |
| RYR.D…N | 113.46 | 25 | 0.0 |
| T.IQYT | 113.46 | 25 | 0.0 |
| T.PPG.PE | 113.46 | 25 | 0.0 |
| T.QPD..D | 113.46 | 25 | 0.0 |
| TG..LS.NG | 113.46 | 25 | 0.0 |
| TT.DG.S.T | 113.46 | 25 | 0.0 |
| VTVQW | 113.46 | 25 | 0.0 |
| W…QP.WP | 113.46 | 25 | 0.0 |
| W..VS.PC | 113.46 | 25 | 0.0 |
| WATVS | 113.46 | 25 | 0.0 |
| WQ..QE.E | 113.46 | 25 | 0.0 |
| YGEHS | 113.46 | 25 | 0.0 |
| YTS.A.D | 113.46 | 25 | 0.0 |
| NQEN.S.V | 112.55 | 7 | 0.0 |
| A.KVTW | 111.95 | 31 | 0.0 |
| AK.AAKAA | 111.95 | 15 | 0.0 |
| EKGETG..G | 111.95 | 37 | 0.0 |
| YK.DL.WL | 111.19 | 7 | 0.0 |
| AYDLQ.D | 108.92 | 7 | 0.0 |
| AYDLQS | 108.92 | 7 | 0.0 |
| G..GLGVG | 108.92 | 15 | 0.0 |
| GD.GP.GN | 108.92 | 24 | 0.0 |
| GDPH.T | 108.92 | 28 | 0.0 |
| LY.EAW.K | 108.92 | 7 | 0.0 |
| NPGPVG | 108.92 | 29 | 0.0 |
| NY..Q.TCL | 108.92 | 7 | 0.0 |
| QA..SD..Y | 108.92 | 7 | 0.0 |
| QKGHY | 108.92 | 7 | 0.0 |
| W.KD..NI | 108.92 | 11 | 0.0 |
| GYDL..DA | 107.63 | 7 | 0.0 |
| RGDDG..G | 105.90 | 35 | 0.0 |
| GA.G.RGM | 104.38 | 23 | 0.0 |
| GCQ..CT | 104.38 | 23 | 0.0 |
| GGQC.H | 104.38 | 23 | 0.0 |
| P..TGPVG | 104.38 | 23 | 0.0 |
| P.M.V.GPP | 104.38 | 23 | 0.0 |
| PGGV.GA | 104.38 | 15 | 0.0 |
| PNG.DG..G | 104.38 | 23 | 0.0 |
| Q.N.SSVLY | 104.38 | 7 | 0.0 |
| QLFPGG | 104.38 | 23 | 0.0 |
| T.VCHC | 104.38 | 23 | 0.0 |
| V..IV.C.K | 104.38 | 23 | 0.0 |
| VGPMG..G | 104.38 | 23 | 0.0 |
| WT..PD..D | 104.38 | 7 | 0.0 |
| A…YDLQS | 103.25 | 7 | 0.0 |
| RP.KPE.PP | 102.87 | 5 | 0.0 |
| RPGKPE.P | 102.87 | 5 | 0.0 |
| GVG.GV.G | 102.11 | 15 | 0.0 |
| TTS.P.S.T | 100.67 | 81 | 0.0 |
| AAKAAQ | 99.85 | 15 | 0.0 |
| DGRHC | 99.85 | 22 | 0.0 |
| K.KGHY | 99.85 | 8 | 0.0 |
| L.S.N.YKS | 99.85 | 7 | 0.0 |
| VA..AQL.A | 99.85 | 33 | 0.0 |
| VP..SEE.R | 99.85 | 22 | 0.0 |
| Q..VSD..Y | 98.55 | 7 | 0.0 |
| G.TGD.GP | 98.33 | 27 | 0.0 |
| AA.AAK.GA | 98.03 | 15 | 0.0 |
| AKAAK.GA | 98.03 | 15 | 0.0 |
| EI.SE.KY | 97.58 | 7 | 0.0 |
| KE..RKQ.G | 97.58 | 7 | 0.0 |
| R.A.K.TW | 97.58 | 31 | 0.0 |
| AAA.KAA | 96.82 | 15 | 0.0 |
| KRNDQ | 96.82 | 32 | 0.0 |
| SS.LYKE | 95.96 | 7 | 0.0 |
| EG.PVG.V | 95.31 | 21 | 0.0 |
| FCN.T.G | 95.31 | 21 | 0.0 |
| G.EGTKG | 95.31 | 21 | 0.0 |
| GA.GL.GK | 95.31 | 21 | 0.0 |
| GP.CFM | 95.31 | 21 | 0.0 |
| K..YN.W.K | 95.31 | 7 | 0.0 |
| KT.Y.TP | 95.31 | 7 | 0.0 |
| M.TS..TT | 95.31 | 21 | 0.0 |
| N.K.T..VM | 95.31 | 7 | 0.0 |
| SDR.YR | 95.31 | 7 | 0.0 |
| SPG.EG..G | 95.31 | 21 | 0.0 |
| YE.TK…H | 95.31 | 7 | 0.0 |
| YV.DTP | 95.31 | 7 | 0.0 |
| AAAKAAA | 94.40 | 15 | 0.0 |
| AAKAA.K | 94.40 | 15 | 0.0 |
| AKIQSD..Y | 93.79 | 7 | 0.0 |
| AKIQSDR | 93.79 | 7 | 0.0 |
| CQ.LVSD | 93.79 | 7 | 0.0 |
| I.AR.AYD | 93.79 | 7 | 0.0 |
| IQSDREY.K | 93.79 | 7 | 0.0 |
| KIQSD..YK | 93.79 | 7 | 0.0 |
| NQ.NIS..L | 93.79 | 7 | 0.0 |
| S…GPVTV | 93.79 | 31 | 0.0 |
| S.VLYKE | 93.79 | 7 | 0.0 |
| SR.IAS.Y | 93.79 | 7 | 0.0 |
| SV.YKE..G | 93.79 | 7 | 0.0 |
| T.STT.T.I | 93.79 | 9 | 0.0 |
| A…FRV.C | 90.77 | 20 | 0.0 |
| DPN.GCA | 90.77 | 20 | 0.0 |
| DREYKK | 90.77 | 7 | 0.0 |
| G.HGEKG | 90.77 | 20 | 0.0 |
| GVPGAI | 90.77 | 15 | 0.0 |
| K..LH.WT | 90.77 | 7 | 0.0 |
| LEW.RG.G | 90.77 | 7 | 0.0 |
| PG..GY.GR | 90.77 | 41 | 0.0 |
| PTPK..W | 90.77 | 20 | 0.0 |
| RFPS.P | 90.77 | 20 | 0.0 |
| VLYKE…K | 90.77 | 7 | 0.0 |
| VSATV..T | 90.77 | 6 | 0.0 |
| YRKQLG | 90.77 | 7 | 0.0 |
| SD..YK..Y | 90.01 | 7 | 0.0 |
| AKKCQ.L | 89.47 | 7 | 0.0 |
| DL.W.RGI | 89.47 | 7 | 0.0 |
| KASRD..S | 89.47 | 7 | 0.0 |
| KQ.GHH | 89.47 | 7 | 0.0 |
| P…KFTS | 89.47 | 7 | 0.0 |
| Q.GHH.G | 89.47 | 7 | 0.0 |
| PGA.VPG | 88.95 | 20 | 0.0 |
| MG.TG.PG | 88.50 | 39 | 0.0 |
| T..QEYE | 88.50 | 32 | 0.0 |
| A..SR.IAS | 88.29 | 7 | 0.0 |
| REYKK..E | 88.05 | 7 | 0.0 |
| CC.W.D | 87.74 | 29 | 0.0 |
| TQ..IM..T | 87.74 | 10 | 0.0 |
| VVPE.K.P | 87.74 | 3 | 0.0 |
| T.EM.R.K | 86.88 | 7 | 0.0 |
| WL.GIG | 86.64 | 7 | 0.0 |
| AK.SRDIAS | 86.23 | 7 | 0.0 |
| ASRDIAS.Y | 86.23 | 7 | 0.0 |
| D..YRV..P | 86.23 | 19 | 0.0 |
| D.ASD..YK | 86.23 | 7 | 0.0 |
| D.IS..KY | 86.23 | 7 | 0.0 |
| DVSYP | 86.23 | 19 | 0.0 |
| H.G.CQ..P | 86.23 | 19 | 0.0 |
| H.VPGK | 86.23 | 19 | 0.0 |
| I…DV.KC | 86.23 | 7 | 0.0 |
| L.GP.CF | 86.23 | 19 | 0.0 |
| P.G.AGL.G | 86.23 | 41 | 0.0 |
| PQN.Q…A | 86.23 | 19 | 0.0 |
| QP.PI.M | 86.23 | 19 | 0.0 |
| R.GGVL..P | 86.23 | 19 | 0.0 |
| RP..P.QPV | 86.23 | 19 | 0.0 |
| SRDIASD | 86.23 | 7 | 0.0 |
| W.KG.GW | 86.23 | 7 | 0.0 |
| Y..Y.HQW | 86.23 | 7 | 0.0 |
| QSD..YK | 86.05 | 7 | 0.0 |
| QS.REYK | 85.82 | 7 | 0.0 |
| E.G.PGT.G | 85.58 | 42 | 0.0 |
| DDPKM..S | 85.32 | 7 | 0.0 |
| GA..I.DD | 85.32 | 7 | 0.0 |
| VA.IQSD | 85.32 | 7 | 0.0 |
| IPI.AAK | 85.10 | 7 | 0.0 |
| M.VAK.QS | 85.10 | 7 | 0.0 |
| ER..R.QEN | 84.72 | 7 | 0.0 |
| GIGW.P | 84.72 | 7 | 0.0 |
| GM.GS.G.P | 84.72 | 28 | 0.0 |
| KG.GC…D | 84.72 | 7 | 0.0 |
| QS.REY.K | 83.96 | 7 | 0.0 |
| T.TPVTT.T | 83.34 | 81 | 0.0 |
| AAKA.R.I | 81.69 | 7 | 0.0 |
| LC..YQ.R | 81.69 | 27 | 0.0 |
| NM..RLY.E | 81.69 | 7 | 0.0 |
| R.ERP.Y | 81.69 | 27 | 0.0 |
| A.KAY.LQ | 81.69 | 7 | 0.0 |
| KGN.GP.G | 81.69 | 28 | 0.0 |
| W..AP.VD | 81.69 | 18 | 0.0 |
| T.T.PPPTT | 81.04 | 87 | 0.0 |
| GIGW…G | 80.87 | 7 | 0.0 |
| PGVG..PG | 80.78 | 18 | 0.0 |
| N.SSV.YK | 80.40 | 7 | 0.0 |
| N.EDD..M | 79.88 | 32 | 0.0 |
| PGGVA.A | 79.88 | 15 | 0.0 |
| SD.LYK | 79.60 | 7 | 0.0 |
| DG.EG.KG | 78.67 | 26 | 0.0 |
| HEWTC | 78.67 | 7 | 0.0 |
| KGKLG.PG | 78.67 | 26 | 0.0 |
| RPGPPV | 78.67 | 20 | 0.0 |
| Y…W.KDK | 78.29 | 7 | 0.0 |
| AAAKAA | 78.20 | 15 | 0.0 |
| W..PD.DG | 77.80 | 6 | 0.0 |
| CQ..SCP | 77.15 | 17 | 0.0 |
| CYNQ..C | 77.15 | 17 | 0.0 |
| I.AAKA.R | 77.15 | 7 | 0.0 |
| PPV.F…D | 77.15 | 17 | 0.0 |
| P..GVG.GG | 76.25 | 29 | 0.0 |
| A..GQ.T.I | 75.64 | 25 | 0.0 |
| AD.KW.R | 75.64 | 25 | 0.0 |
| CSW.Q..R | 75.64 | 25 | 0.0 |
| D.P.LCA | 75.64 | 25 | 0.0 |
| DC..RVT | 75.64 | 25 | 0.0 |
| FDG.AT | 75.64 | 25 | 0.0 |
| G..GLIGPP | 75.64 | 29 | 0.0 |
| G.PGHPG.E | 75.64 | 25 | 0.0 |
| GLPGH.GQ | 75.64 | 25 | 0.0 |
| GP.GEQGE | 75.64 | 25 | 0.0 |
| GPP.E.I.P | 75.64 | 25 | 0.0 |
| H.GKEG..G | 75.64 | 25 | 0.0 |
| I..YI.EK | 75.64 | 7 | 0.0 |
| K.TSI.D.P | 75.64 | 7 | 0.0 |
| MG.SSG..Y | 75.64 | 25 | 0.0 |
| PGPPGEV | 75.64 | 25 | 0.0 |
| Q.QAI.QQ | 75.64 | 25 | 0.0 |
| QAQ.ILQ | 75.64 | 25 | 0.0 |
| SD..YK.D | 75.64 | 7 | 0.0 |
| T.R.SCT | 75.64 | 25 | 0.0 |
| V.E.Y..QT | 75.64 | 25 | 0.0 |
| H..PDTP.I | 75.64 | 7 | 0.0 |
| CQTLVSD | 74.88 | 7 | 0.0 |
| DFEKW.T | 74.88 | 7 | 0.0 |
| DV.YKN.L | 74.88 | 7 | 0.0 |
| E.VLAKN | 74.88 | 7 | 0.0 |
| GI..VPI.S | 74.88 | 7 | 0.0 |
| KDF.KW.T | 74.88 | 7 | 0.0 |
| KRA.EI.S | 74.88 | 7 | 0.0 |
| SD..YKN.L | 74.88 | 7 | 0.0 |
| SDV.YKN | 74.88 | 7 | 0.0 |
| T..LSD..Y | 74.88 | 7 | 0.0 |
| VLA.KC.T | 74.88 | 7 | 0.0 |
| YK.D.EKW | 74.88 | 7 | 0.0 |
| YK.DLQ | 74.88 | 7 | 0.0 |
| R.K.NQ.N | 74.63 | 7 | 0.0 |
| VAK.QSD | 74.43 | 7 | 0.0 |
| Y..QSD..Y | 74.13 | 7 | 0.0 |
| YEKSK | 73.91 | 7 | 0.0 |
| T.I.DTP | 73.75 | 7 | 0.0 |
| CE.CP.N | 72.62 | 16 | 0.0 |
| CSGA.F | 72.62 | 16 | 0.0 |
| CVP.K.C | 72.62 | 16 | 0.0 |
| DP..MW | 72.62 | 7 | 0.0 |
| FE.WKTK | 72.62 | 7 | 0.0 |
| GFTCV | 72.62 | 16 | 0.0 |
| GP..FP.Y | 72.62 | 16 | 0.0 |
| GP.GA.G.T | 72.62 | 21 | 0.0 |
| I.SD..YR | 72.62 | 7 | 0.0 |
| NFTPV | 72.62 | 32 | 0.0 |
| PDTPEI | 72.62 | 7 | 0.0 |
| QRG.VG..G | 72.62 | 24 | 0.0 |
| S..CQN..C | 72.62 | 16 | 0.0 |
| S.CS..YC | 72.62 | 16 | 0.0 |
| S.PVDM..V | 72.62 | 7 | 0.0 |
| T..G.TCV | 72.62 | 24 | 0.0 |
| TI.WE.P | 72.62 | 6 | 0.0 |
| VAGCK | 72.62 | 16 | 0.0 |
| YK..YE..K | 72.62 | 7 | 0.0 |
| AK..SD..Y | 71.40 | 7 | 0.0 |
| H..PDTPE | 71.10 | 7 | 0.0 |
| HHIGA | 71.10 | 7 | 0.0 |
| K.SSPVDM | 71.10 | 7 | 0.0 |
| KWKTK.S | 71.10 | 7 | 0.0 |
| LG.VL.K.C | 71.10 | 7 | 0.0 |
| ML..VLAK | 71.10 | 7 | 0.0 |
| PG..PGGV | 71.10 | 15 | 0.0 |
| PGHPG..GE | 71.10 | 27 | 0.0 |
| SD..YRQ | 71.10 | 7 | 0.0 |
| TKFSSPV | 71.10 | 7 | 0.0 |
| WKTKFS | 71.10 | 7 | 0.0 |
| P.RPGKP.G | 70.80 | 5 | 0.0 |
| PP.RP.KPE | 70.80 | 5 | 0.0 |
| TTS.P.T | 70.80 | 89 | 0.0 |
| KGYDL | 70.60 | 23 | 0.0 |
| T..ATS | 70.51 | 117 | 0.0 |
| CS.TS.R | 70.35 | 31 | 0.0 |
| QG.FG..G | 70.35 | 31 | 0.0 |
| T…GYK.R | 70.35 | 31 | 0.0 |
| YK..Y..QL | 70.35 | 7 | 0.0 |
| DI.SD.KY | 70.02 | 7 | 0.0 |
| DL..DAIP | 70.02 | 7 | 0.0 |
| DM..VV.AK | 70.02 | 7 | 0.0 |
| I.EKRE..R | 70.02 | 6 | 0.0 |
| K.LYT..W | 70.02 | 7 | 0.0 |
| KW..K..SP | 70.02 | 7 | 0.0 |
| LAKKCQ | 70.02 | 7 | 0.0 |
| LYTE.W | 70.02 | 7 | 0.0 |
| PVDML..V | 70.02 | 7 | 0.0 |
| T.Y.VE..D | 70.02 | 6 | 0.0 |
| VV.AKK.Q | 70.02 | 7 | 0.0 |
| E.GHPG..G | 69.89 | 50 | 0.0 |
| A…QSDN | 69.59 | 7 | 0.0 |
| G.NGPPGP | 69.59 | 27 | 0.0 |
| GQRG..GA | 69.59 | 23 | 0.0 |
| GVLGG.G | 69.59 | 15 | 0.0 |
| NGPPGP | 69.59 | 27 | 0.0 |
| NGPPGP.G | 69.59 | 27 | 0.0 |
| D.R.DAI | 69.37 | 9 | 0.0 |
| RQ.PD..K | 69.21 | 7 | 0.0 |
| SPVDML | 69.21 | 7 | 0.0 |
| VG..P..GV | 69.21 | 16 | 0.0 |
| YKK.F.K.K | 69.21 | 7 | 0.0 |
| GEKGH.G | 68.72 | 32 | 0.0 |
| EK.TL.W | 68.08 | 6 | 0.0 |
| G..GD.GAQ | 68.08 | 30 | 0.0 |
| G.GAGVPG | 68.08 | 15 | 0.0 |
| G.GPF…Q | 68.08 | 15 | 0.0 |
| GAGIPG | 68.08 | 15 | 0.0 |
| GVPGA..G | 68.08 | 15 | 0.0 |
| KLY.EA..K | 68.08 | 5 | 0.0 |
| PGVGGA | 68.08 | 18 | 0.0 |
| PI..GG.C | 68.08 | 15 | 0.0 |
| PMVD..L | 68.08 | 15 | 0.0 |
| TTP.V…E | 68.08 | 15 | 0.0 |
| VPG.G.PG | 68.08 | 16 | 0.0 |
| VT..KC.L | 68.08 | 6 | 0.0 |
| S.YKYK | 67.73 | 7 | 0.0 |
| DN.YK.D | 67.43 | 7 | 0.0 |
| A.G.TGP.G | 66.56 | 26 | 0.0 |
| TGPGG..G | 66.56 | 22 | 0.0 |
| YK.AYE | 65.56 | 7 | 0.0 |
| FV.V.VLD | 65.35 | 6 | 0.0 |
| YIV.K.D | 65.35 | 6 | 0.0 |
| TP.DM.S | 65.05 | 7 | 0.0 |
| S.I.GY..E | 65.00 | 6 | 0.0 |
| E.AN.TL | 64.83 | 25 | 0.0 |
| EY.FR..AE | 64.83 | 6 | 0.0 |
| K..YE..KG | 64.67 | 7 | 0.0 |
| GVPGV.G | 64.55 | 22 | 0.0 |
| W..PL.DGG | 64.36 | 6 | 0.0 |
| Y…YEK.K | 64.33 | 7 | 0.0 |
| G..IT.Y.V | 63.93 | 6 | 0.0 |
| AAK.SR..A | 63.54 | 7 | 0.0 |
| CC..N…Y | 63.54 | 28 | 0.0 |
| CEE.C.P | 63.54 | 22 | 0.0 |
| D.DYKH | 63.54 | 7 | 0.0 |
| D.P.I..AK | 63.54 | 7 | 0.0 |
| DG.QGP.G | 63.54 | 33 | 0.0 |
| E.GSM.E | 63.54 | 1 | 0.0 |
| E.KYRQ..D | 63.54 | 7 | 0.0 |
| EK.KAKK | 63.54 | 7 | 0.0 |
| G.PGG.GN | 63.54 | 21 | 0.0 |
| GE.GHPG | 63.54 | 50 | 0.0 |
| IL.AK..Y | 63.54 | 7 | 0.0 |
| KGKM.G | 63.54 | 7 | 0.0 |
| KT.AK…Y | 63.54 | 7 | 0.0 |
| L..KD.T.W | 63.54 | 14 | 0.0 |
| LGRPPP | 63.54 | 14 | 0.0 |
| LYK.AW | 63.54 | 7 | 0.0 |
| MLP.RF | 63.54 | 21 | 0.0 |
| N.YKAD | 63.54 | 7 | 0.0 |
| NY.VE.R | 63.54 | 6 | 0.0 |
| NYE.TK | 63.54 | 7 | 0.0 |
| P.G.Y.DR | 63.54 | 14 | 0.0 |
| PD..QF..A | 63.54 | 7 | 0.0 |
| PDTPD.L | 63.54 | 7 | 0.0 |
| PGV.G.GV | 63.54 | 21 | 0.0 |
| Q.SD..YK | 63.54 | 7 | 0.0 |
| RK.LG..IG | 63.54 | 7 | 0.0 |
| SE..YRQ | 63.54 | 7 | 0.0 |
| T.PG..CP | 63.54 | 18 | 0.0 |
| T.VH.MP | 63.54 | 7 | 0.0 |
| VG.TSC | 63.54 | 14 | 0.0 |
| W.KGI.C | 63.54 | 7 | 0.0 |
| WE.SK.K | 63.54 | 7 | 0.0 |
| Y..NYE.T | 63.54 | 7 | 0.0 |
| Y.K.YEK | 63.54 | 13 | 0.0 |
| YK.KGE | 63.54 | 7 | 0.0 |
| D…I.AAK | 62.93 | 7 | 0.0 |
| G.DGP.G.K | 62.78 | 44 | 0.0 |
| YIVEK | 62.63 | 6 | 0.0 |
| E..RVK..Q | 62.53 | 13 | 0.0 |
| W.P.GS..V | 62.53 | 7 | 0.0 |
| SDV.YK | 62.33 | 7 | 0.0 |
| G..GE.GES | 62.24 | 29 | 0.0 |
| GY.GR.GP | 62.24 | 29 | 0.0 |
| S.R.D.G.Y | 62.24 | 6 | 0.0 |
| K.CQTLV | 62.03 | 8 | 0.0 |
| DA.PI.A | 61.89 | 7 | 0.0 |
| GG.EI..Y | 61.89 | 6 | 0.0 |
| A.NAAGV | 61.27 | 6 | 0.0 |
| G.NGA.GP | 61.27 | 27 | 0.0 |
| I.SDN.Y | 61.27 | 7 | 0.0 |
| SSPVDM | 61.27 | 7 | 0.0 |
| VEKRE..R | 61.27 | 6 | 0.0 |
| W.KDKT | 61.27 | 7 | 0.0 |
| YG.GEP.E | 61.27 | 6 | 0.0 |
| TPEM.R | 61.06 | 7 | 0.0 |
| D.T.IH..P | 60.94 | 7 | 0.0 |
| GG..I..YI | 60.94 | 6 | 0.0 |
| GW.PI.S | 60.94 | 7 | 0.0 |
| KK.FE..KT | 60.94 | 7 | 0.0 |
| M.K.LYT | 60.94 | 7 | 0.0 |
| PGTAG..G | 60.94 | 28 | 0.0 |
| WD..KT..H | 60.94 | 7 | 0.0 |
| C..GG.C.H | 60.51 | 30 | 0.0 |
| CKK.VT | 60.51 | 20 | 0.0 |
| DGGSK…Y | 60.51 | 6 | 0.0 |
| EAWRQ | 60.51 | 20 | 0.0 |
| GT.GG.G.K | 60.51 | 20 | 0.0 |
| HI.AR…D | 60.51 | 7 | 0.0 |
| KEPAPT.T | 60.51 | 5 | 0.0 |
| MPDTPE | 60.51 | 7 | 0.0 |
| YEK.KG | 60.51 | 7 | 0.0 |
| Y..RV.AEN | 60.35 | 6 | 0.0 |
| D.LYK…E | 60.30 | 7 | 0.0 |
| FPG.SG..G | 59.91 | 27 | 0.0 |
| FT.VTDS | 59.91 | 7 | 0.0 |
| LAL.GPP | 59.91 | 33 | 0.0 |
| P.DDF..PN | 59.91 | 28 | 0.0 |
| T.YIV…E | 59.91 | 6 | 0.0 |
| Y.S.L.W.R | 59.91 | 7 | 0.0 |
| G.EY.FRV | 59.85 | 7 | 0.0 |
| P..DGG..I | 59.71 | 6 | 0.0 |
| GP.GR.G.Q | 59.65 | 27 | 0.0 |
| TG.VGP.G | 59.65 | 27 | 0.0 |
| N.YG.GEP | 59.41 | 6 | 0.0 |
| R.D.GKY | 59.41 | 6 | 0.0 |
| Y..EKRE | 59.41 | 6 | 0.0 |
| G.TGDPG | 59.00 | 33 | 0.0 |
| PGKPEGP | 59.00 | 5 | 0.0 |
| PPPRPG.P | 59.00 | 5 | 0.0 |
| QG.T..TG | 59.00 | 39 | 0.0 |
| QGPPPR.G | 59.00 | 5 | 0.0 |
| C.PM..C | 59.00 | 26 | 0.0 |
| D.KWK.H | 59.00 | 13 | 0.0 |
| EDG.PG.KG | 59.00 | 26 | 0.0 |
| H..QA.D.Q | 59.00 | 7 | 0.0 |
| LKAT.ST | 59.00 | 18 | 0.0 |
| NYSE..Y | 59.00 | 7 | 0.0 |
| S.LC.CL | 59.00 | 26 | 0.0 |
| EY.F.I.A | 58.65 | 6 | 0.0 |
| G.HGPPG | 58.59 | 48 | 0.0 |
| PGESG..G | 58.50 | 33 | 0.0 |
| I..YV.E.R | 58.35 | 6 | 0.0 |
| KPE.PP..V | 58.35 | 4 | 0.0 |
| G..GE.GHP | 58.24 | 50 | 0.0 |
| AA..SCE | 57.49 | 19 | 0.0 |
| AKG.C.A | 57.49 | 19 | 0.0 |
| CEGG..E | 57.49 | 19 | 0.0 |
| CV.PC..G | 57.49 | 19 | 0.0 |
| P…LKFT | 57.49 | 7 | 0.0 |
| P…PKDF | 57.49 | 19 | 0.0 |
| PK.DG..P | 57.49 | 20 | 0.0 |
| W.PPL..GG | 57.49 | 6 | 0.0 |
| FRV.AEN | 57.41 | 6 | 0.0 |
| G.EY.FR | 57.08 | 7 | 0.0 |
| G.E.IFR | 57.05 | 8 | 0.0 |
| GE.GFQG | 57.05 | 44 | 0.0 |
| Y.F.V..EN | 57.05 | 6 | 0.0 |
| D.AYRV | 56.73 | 25 | 0.0 |
| DAIPI | 56.73 | 7 | 0.0 |
| G..GPPGHP | 56.73 | 31 | 0.0 |
| H.MPD.P | 56.73 | 7 | 0.0 |
| P…QAK.N | 56.73 | 7 | 0.0 |
| PA..EP.M | 56.73 | 25 | 0.0 |
| PTTTK…T | 56.73 | 5 | 0.0 |
| PVWER | 56.73 | 25 | 0.0 |
| QAILQQ | 56.73 | 25 | 0.0 |
| RK.TN..G | 56.73 | 25 | 0.0 |
| T..KSAPT | 56.73 | 5 | 0.0 |
| YQEYE | 56.73 | 25 | 0.0 |
| A.N.SD..Y | 56.48 | 7 | 0.0 |
| W..PE..GG | 56.48 | 6 | 0.0 |
| EV..P.PI | 56.28 | 31 | 0.0 |
| R.D.G.Y.V | 56.28 | 7 | 0.0 |
| TP..TA.TT | 56.28 | 11 | 0.0 |
| GGS.I..Y | 56.19 | 6 | 0.0 |
| KT..H..PD | 56.11 | 7 | 0.0 |
| G.DG.QGP | 55.97 | 30 | 0.0 |
| A.VPGV | 55.67 | 34 | 0.0 |
| H…VAK.Q | 55.60 | 7 | 0.0 |
| AK.NA..M | 55.37 | 7 | 0.0 |
| DGGS.I | 55.37 | 6 | 0.0 |
| A.Y.F..PG | 54.46 | 6 | 0.0 |
| AENE.G.G | 54.46 | 6 | 0.0 |
| C.R.D.G.Y | 54.46 | 6 | 0.0 |
| D.G.Y.LT | 54.46 | 6 | 0.0 |
| D.PGPP.GP | 54.46 | 6 | 0.0 |
| DGRW.K | 54.46 | 6 | 0.0 |
| DGV.GP.G | 54.46 | 24 | 0.0 |
| EN.SG.KS | 54.46 | 6 | 0.0 |
| ESC.L.W | 54.46 | 6 | 0.0 |
| EYQFR | 54.46 | 6 | 0.0 |
| FVN.R…T | 54.46 | 6 | 0.0 |
| GG.K.T.Y | 54.46 | 6 | 0.0 |
| GKYT.T | 54.46 | 6 | 0.0 |
| GP..FDE | 54.46 | 6 | 0.0 |
| GPP.GP..I | 54.46 | 6 | 0.0 |
| GPQG.NG | 54.46 | 24 | 0.0 |
| GS.IT.Y | 54.46 | 6 | 0.0 |
| GYIIE | 54.46 | 6 | 0.0 |
| I..YVIE | 54.46 | 6 | 0.0 |
| I.A.N.YG | 54.46 | 6 | 0.0 |
| I.D.TK.S | 54.46 | 6 | 0.0 |
| I.G.VPG | 54.46 | 18 | 0.0 |
| I.GY.LE | 54.46 | 6 | 0.0 |
| I.W.KP..D | 54.46 | 6 | 0.0 |
| ILW.K.N | 54.46 | 6 | 0.0 |
| ITNY..E | 54.46 | 6 | 0.0 |
| K.TSI..T | 54.46 | 7 | 0.0 |
| KVTKL..G | 54.46 | 6 | 0.0 |
| L.EGC.Y | 54.46 | 6 | 0.0 |
| L.W.KP..D | 54.46 | 6 | 0.0 |
| L.W.PP..D | 54.46 | 6 | 0.0 |
| N..GVGEP | 54.46 | 6 | 0.0 |
| N.S.KLY | 54.46 | 7 | 0.0 |
| P..RW.KA | 54.46 | 6 | 0.0 |
| P.DPP..PE | 54.46 | 6 | 0.0 |
| P.SDGGS | 54.46 | 6 | 0.0 |
| PCDPP..P | 54.46 | 6 | 0.0 |
| PTT.K.TA | 54.46 | 5 | 0.0 |
| QGPP.RPG | 54.46 | 8 | 0.0 |
| R.T.I..AG | 54.46 | 6 | 0.0 |
| RE.SR..W | 54.46 | 6 | 0.0 |
| RPGPP.GP | 54.46 | 6 | 0.0 |
| RW.KA..T | 54.46 | 6 | 0.0 |
| RW.KA.F | 54.46 | 6 | 0.0 |
| SG.K..FV | 54.46 | 6 | 0.0 |
| SILW.K | 54.46 | 6 | 0.0 |
| T…K.NEY | 54.46 | 6 | 0.0 |
| T..WD.PL | 54.46 | 6 | 0.0 |
| T.I.KD.M | 54.46 | 6 | 0.0 |
| T.R.N.E.T | 54.46 | 6 | 0.0 |
| THY.VE | 54.46 | 6 | 0.0 |
| V..IGRP | 54.46 | 6 | 0.0 |
| V.DKPGP | 54.46 | 6 | 0.0 |
| V.DVPGP | 54.46 | 6 | 0.0 |
| VLAK.N | 54.46 | 7 | 0.0 |
| VLD.PGP | 54.46 | 6 | 0.0 |
| VLDTP.P | 54.46 | 6 | 0.0 |
| VP..GRP.P | 54.46 | 6 | 0.0 |
| W..PI.DG | 54.46 | 6 | 0.0 |
| WVK.N.T | 54.46 | 6 | 0.0 |
| Y.VEM..K | 54.46 | 6 | 0.0 |
| DAIP..A | 53.86 | 7 | 0.0 |
| PDTP.I | 53.81 | 8 | 0.0 |
| N.SD..YK | 53.76 | 7 | 0.0 |
| D.KYK..Y | 53.16 | 7 | 0.0 |
| PDTPD | 52.95 | 7 | 0.0 |
| PT.PKK..P | 52.95 | 5 | 0.0 |
| D.E.FEG | 52.65 | 29 | 0.0 |
| GEIGP.G | 52.65 | 29 | 0.0 |
| GVGG.PG | 52.65 | 16 | 0.0 |
| L.D.KW.R | 52.65 | 29 | 0.0 |
| E…Y.FRV | 52.52 | 6 | 0.0 |
| V.G.VG.PG | 52.44 | 46 | 0.0 |
| DTPEI | 52.37 | 7 | 0.0 |
| DTPE…A | 52.30 | 8 | 0.0 |
| EYWV.P | 52.19 | 23 | 0.0 |
| V.D.PGPP | 52.19 | 6 | 0.0 |
| V.EP.M.V | 52.19 | 23 | 0.0 |
| K.PAPTT | 52.11 | 5 | 0.0 |
| S..DDPK | 51.99 | 7 | 0.0 |
| E…KGY.L | 51.87 | 7 | 0.0 |
| K.KFT…D | 51.87 | 7 | 0.0 |
| M..RLY.E | 51.87 | 7 | 0.0 |
| TE.WD..K | 51.87 | 7 | 0.0 |
| A..PI.PPG | 51.74 | 9 | 0.0 |
| TS.P.T | 51.53 | 123 | 0.0 |
| DDPK…S | 51.44 | 7 | 0.0 |
| G.FGR.C | 51.44 | 17 | 0.0 |
| NYK.K.E | 51.44 | 10 | 0.0 |
| P..AAKA | 51.44 | 29 | 0.0 |
| P.G.WG..C | 51.44 | 16 | 0.0 |
| VG..SC..R | 51.44 | 17 | 0.0 |
| PA.TTPK | 51.30 | 5 | 0.0 |
| P..DGGS | 51.18 | 6 | 0.0 |
| W.PP..DG | 51.13 | 6 | 0.0 |
| AKAN..N | 50.83 | 7 | 0.0 |
| G..LSD..Y | 50.83 | 7 | 0.0 |
| H.P.DM.S | 50.83 | 7 | 0.0 |
| KQ.WE..K | 50.83 | 7 | 0.0 |
| KT..HT..D | 50.83 | 7 | 0.0 |
| KY..HP.T | 50.83 | 7 | 0.0 |
| LKG.GC | 50.83 | 7 | 0.0 |
| ST.KAY | 50.83 | 22 | 0.0 |
| T…PA.TC | 50.83 | 28 | 0.0 |
| T…TP.DM | 50.83 | 7 | 0.0 |
| VEK.KKA | 50.83 | 7 | 0.0 |
| VPE.P..VV | 50.83 | 3 | 0.0 |
| W…K.DGG | 50.83 | 6 | 0.0 |
| Y…PDAM | 50.83 | 7 | 0.0 |
| PAPTT.KE | 50.72 | 5 | 0.0 |
| GKPEGPP | 50.57 | 5 | 0.0 |
| TGL.E…Y | 50.43 | 6 | 0.0 |
| EG..Y.FR | 50.35 | 6 | 0.0 |
| DGGS.V.G | 50.34 | 7 | 0.0 |
| A.DPI..P | 50.27 | 6 | 0.0 |
| RETS…W | 50.27 | 6 | 0.0 |
| T.K.P.PTT | 50.16 | 5 | 0.0 |
| TTP.E..PT | 50.16 | 5 | 0.0 |
| T..P.T | 50.16 | 187 | 0.0 |
| EY.FRV.A | 50.11 | 7 | 0.0 |
| APTTTK | 49.92 | 5 | 0.0 |
| DGG.EI | 49.92 | 6 | 0.0 |
| G..CEG..D | 49.92 | 22 | 0.0 |
| GY.LE..E | 49.92 | 6 | 0.0 |
| IPIV..K | 49.92 | 7 | 0.0 |
| TT.KEP..T | 49.92 | 5 | 0.0 |
| W..PV..GG | 49.92 | 6 | 0.0 |
| YKEN.G | 49.92 | 7 | 0.0 |
| EY.FR..A | 49.57 | 7 | 0.0 |
| FRI.A.N | 49.51 | 6 | 0.0 |
| HPGPPG | 49.51 | 37 | 0.0 |
| P.HDGG | 49.51 | 6 | 0.0 |
| PK..APTTP | 49.51 | 5 | 0.0 |
| SD.KYK | 49.51 | 10 | 0.0 |
| G.VGDPG | 49.42 | 30 | 0.0 |
| GPPGHPG | 49.42 | 30 | 0.0 |
| I.LAK.N | 49.42 | 7 | 0.0 |
| Y..HPD..K | 49.42 | 7 | 0.0 |
| G..GFPGF | 49.36 | 62 | 0.0 |
| T.YIVE | 49.27 | 6 | 0.0 |
| I.NYI.E | 49.21 | 7 | 0.0 |
| K..APTT | 49.17 | 5 | 0.0 |
| EE.K..GY | 49.02 | 7 | 0.0 |
| G.KGD.G.D | 49.02 | 34 | 0.0 |
| P..A.DPI | 49.02 | 6 | 0.0 |
| P.PTTPK | 49.02 | 5 | 0.0 |
| T…VSL.W | 49.02 | 6 | 0.0 |
| Y..ERRE | 49.02 | 6 | 0.0 |
| W..P..DG | 48.93 | 6 | 0.0 |
| ASD.KY | 48.88 | 7 | 0.0 |
| PG.IGL.G | 48.88 | 47 | 0.0 |
| DG.AKI | 48.79 | 7 | 0.0 |
| GE.GETG | 48.79 | 41 | 0.0 |
| KG..IG..S | 48.79 | 7 | 0.0 |
| APTTPK.PA | 48.63 | 5 | 0.0 |
| PTTPKEPAP | 48.63 | 5 | 0.0 |
| TTPKEPAPT | 48.63 | 5 | 0.0 |
| FST.LVT | 48.41 | 4 | 0.0 |
| GVPG.G.P | 48.41 | 16 | 0.0 |
| V..RPGPP | 48.41 | 6 | 0.0 |
| GP.GKAG | 47.98 | 37 | 0.0 |
| Q..GPPPRP | 47.98 | 5 | 0.0 |
| VK.F.DG | 47.98 | 25 | 0.0 |
| I.DTPE | 47.86 | 7 | 0.0 |
| R..AEN..G | 47.77 | 6 | 0.0 |
| D.NYK.K | 47.65 | 7 | 0.0 |
| GYH.E.K | 47.65 | 6 | 0.0 |
| GYIVE | 47.65 | 6 | 0.0 |
| I.GY.VE | 47.65 | 6 | 0.0 |
| K..YE.TK | 47.65 | 7 | 0.0 |
| K.P.APP.K | 47.65 | 3 | 0.0 |
| K.YKA..E | 47.65 | 7 | 0.0 |
| P.GSL..EK | 47.65 | 7 | 0.0 |
| DGP.GP.G | 47.56 | 51 | 0.0 |
| G..GH.G.K | 47.52 | 51 | 0.0 |
| YS..LYK | 47.40 | 7 | 0.0 |
| PTTP.EP | 47.36 | 5 | 0.0 |
| C.CDGG | 47.20 | 26 | 0.0 |
| G..GL.GHK | 47.20 | 26 | 0.0 |
| PK.VVP.K | 47.20 | 3 | 0.0 |
| PK.VVPE | 47.20 | 3 | 0.0 |
| V.S.G..TA | 47.11 | 91 | 0.0 |
| V.D.PGP | 47.10 | 6 | 0.0 |
| N..GVG.P | 47.03 | 6 | 0.0 |
| PE..RVK | 46.99 | 7 | 0.0 |
| EYW.D…G | 46.90 | 31 | 0.0 |
| G.CPA…Y | 46.90 | 26 | 0.0 |
| G.QG.FG | 46.90 | 31 | 0.0 |
| PTT.KEP | 46.90 | 5 | 0.0 |
| TG..Q.TN | 46.90 | 31 | 0.0 |
| T..S…T | 46.86 | 204 | 0.0 |
| LD.PGPP | 46.84 | 9 | 0.0 |
| V…D.PGP | 46.80 | 9 | 0.0 |
| T…EG.EY | 46.75 | 7 | 0.0 |
| AAG.S.PS | 46.68 | 6 | 0.0 |
| EN.SG.K.A | 46.68 | 6 | 0.0 |
| P.GPI.F | 46.68 | 6 | 0.0 |
| PG.PS.P.V | 46.68 | 6 | 0.0 |
| PG.PTGP | 46.68 | 6 | 0.0 |
| RVCA.N | 46.68 | 6 | 0.0 |
| S.V.GY..E | 46.68 | 6 | 0.0 |
| EG.EY..R | 46.56 | 6 | 0.0 |
| G.DGP.GP | 46.47 | 48 | 0.0 |
| YRQ.PD | 46.14 | 7 | 0.0 |
| FR.KA.N | 46.08 | 6 | 0.0 |
| DGGS..T | 45.99 | 6 | 0.0 |
| LAK.N..N | 45.99 | 7 | 0.0 |
| V.A.N.YG | 45.75 | 6 | 0.0 |
| P.YDGG | 45.68 | 6 | 0.0 |
| A.AAAAAK | 45.38 | 15 | 0.0 |
| A.F.CE.S | 45.38 | 6 | 0.0 |
| A.N.K..SD | 45.38 | 6 | 0.0 |
| A.N.YG.G | 45.38 | 6 | 0.0 |
| A.TTTK.P | 45.38 | 5 | 0.0 |
| APTTPK | 45.38 | 5 | 0.0 |
| C.LSW..P | 45.38 | 6 | 0.0 |
| D..KFT..T | 45.38 | 7 | 0.0 |
| D.KLVH | 45.38 | 7 | 0.0 |
| DGGA.I | 45.38 | 6 | 0.0 |
| E.R.EG..Y | 45.38 | 20 | 0.0 |
| E.VGP..C | 45.38 | 20 | 0.0 |
| EG.EYE | 45.38 | 20 | 0.0 |
| EPGML | 45.38 | 25 | 0.0 |
| G.GYGGD | 45.38 | 4 | 0.0 |
| GCY..DT | 45.38 | 11 | 0.0 |
| GPI.F..V | 45.38 | 6 | 0.0 |
| I.S.K.YR | 45.38 | 10 | 0.0 |
| KE.VPEK | 45.38 | 3 | 0.0 |
| KYKE.Y | 45.38 | 9 | 0.0 |
| MM.PP.V | 45.38 | 15 | 0.0 |
| P..PPQPV | 45.38 | 18 | 0.0 |
| P.FDGG | 45.38 | 6 | 0.0 |
| PGEGG..G | 45.38 | 25 | 0.0 |
| Q.SDI.Y | 45.38 | 9 | 0.0 |
| R.D.G.Y.I | 45.38 | 6 | 0.0 |
| TPKEPAPTT | 45.38 | 5 | 0.0 |
| VC..W.PP | 45.38 | 15 | 0.0 |
| YG.GEP | 45.38 | 9 | 0.0 |
| N..GIG.P | 44.99 | 6 | 0.0 |
| EN..G.G.P | 44.82 | 8 | 0.0 |
| P.KPE.PP | 44.78 | 8 | 0.0 |
| V.AEN..G | 44.74 | 6 | 0.0 |
| GEP.ES | 44.63 | 29 | 0.0 |
| CS..DG.C | 44.48 | 17 | 0.0 |
| LE.EK.K.A | 44.48 | 7 | 0.0 |
| YK..WE..K | 44.48 | 7 | 0.0 |
| A.FECE | 44.25 | 6 | 0.0 |
| K.NAL.M | 44.09 | 7 | 0.0 |
| TPV.TST | 44.04 | 81 | 0.0 |
| SD..YK | 44.03 | 10 | 0.0 |
| G..YEFR | 44.02 | 7 | 0.0 |
| GG.P..GY | 43.99 | 6 | 0.0 |
| T…DTP.I | 43.87 | 8 | 0.0 |
| Y.G.QGP.G | 43.87 | 29 | 0.0 |
| GPLG.PG | 43.64 | 69 | 0.0 |
| ARDPC | 43.57 | 6 | 0.0 |
| I.KDSM | 43.57 | 6 | 0.0 |
| IVEKR | 43.57 | 6 | 0.0 |
| K.C.R.D.G | 43.57 | 6 | 0.0 |
| KPS.P.EP | 43.57 | 6 | 0.0 |
| L.DG.W.K | 43.57 | 6 | 0.0 |
| P.NDGG | 43.57 | 6 | 0.0 |
| R..VSL.W | 43.57 | 6 | 0.0 |
| RS.PL..PP | 43.57 | 19 | 0.0 |
| S…TW.PP | 43.57 | 6 | 0.0 |
| T..RTT.K | 43.57 | 6 | 0.0 |
| W.K.SFT | 43.57 | 6 | 0.0 |
| Y.IEK.E | 43.57 | 6 | 0.0 |
| E..EYEF | 43.25 | 9 | 0.0 |
| S..KYKE | 43.18 | 7 | 0.0 |
| GPRGQ.G | 43.12 | 32 | 0.0 |
| P..TC.DL | 43.12 | 38 | 0.0 |
| Y.E.W…K | 42.91 | 7 | 0.0 |
| C.H.TG.C | 42.79 | 13 | 0.0 |
| GWAGA | 42.79 | 33 | 0.0 |
| W..PNQ.C | 42.79 | 33 | 0.0 |
| I…GRP.P | 42.59 | 31 | 0.0 |
| P.GLRG..G | 42.59 | 34 | 0.0 |
| GG.PI..Y | 42.55 | 6 | 0.0 |
| VLD.PG | 42.55 | 6 | 0.0 |
| GG.R..GY | 42.36 | 10 | 0.0 |
| H.YT..PD | 42.36 | 7 | 0.0 |
| KAK.F…V | 42.36 | 7 | 0.0 |
| L.EG..YY | 42.36 | 6 | 0.0 |
| M..PFM.P | 42.36 | 14 | 0.0 |
| NF..G..TC | 42.36 | 28 | 0.0 |
| P.P.TT.SA | 42.36 | 7 | 0.0 |
| P.Y.KS..P | 42.36 | 14 | 0.0 |
| PG..GPPGV | 42.36 | 49 | 0.0 |
| PP.R.G.I | 42.36 | 14 | 0.0 |
| PTP.T…E | 42.36 | 24 | 0.0 |
| QWQQ..Q | 42.36 | 14 | 0.0 |
| RE..PT..A | 42.36 | 14 | 0.0 |
| SDI.YK | 42.36 | 7 | 0.0 |
| ST.WR.G | 42.36 | 14 | 0.0 |
| TP..DPK | 42.36 | 14 | 0.0 |
| V.LKW..P | 42.36 | 6 | 0.0 |
| VEKRD | 42.36 | 6 | 0.0 |
| Y.P.F..SP | 42.36 | 14 | 0.0 |
| YRK.YE | 42.36 | 7 | 0.0 |
| V.L.W.KP | 42.08 | 6 | 0.0 |
| PGPPGPPGE | 41.98 | 35 | 0.0 |
| K..M.V.W | 41.89 | 6 | 0.0 |
| APTT.K | 41.86 | 6 | 0.0 |
| E.T.KA..T | 41.75 | 17 | 0.0 |
| G..GY.GR | 41.75 | 46 | 0.0 |
| Y..RVSA | 41.75 | 7 | 0.0 |
| DPKLV | 41.49 | 7 | 0.0 |
| F…VVG.P | 41.49 | 21 | 0.0 |
| G.PGASG | 41.49 | 45 | 0.0 |
| HP.PPG.P | 41.49 | 35 | 0.0 |
| P.RTC.D | 41.49 | 32 | 0.0 |
| Y.PGPP..P | 41.49 | 8 | 0.0 |
| GFPGF.G | 41.41 | 47 | 0.0 |
| PG.KGN.G | 41.41 | 48 | 0.0 |
| GGYGGD | 41.15 | 4 | 0.0 |
| YEFRV | 41.10 | 7 | 0.0 |
| A.C.PI.G | 40.85 | 12 | 0.0 |
| A.G.HCE | 40.85 | 18 | 0.0 |
| D.G.Y.I.A | 40.85 | 6 | 0.0 |
| F.VIAK | 40.85 | 6 | 0.0 |
| FGVG.P | 40.85 | 6 | 0.0 |
| G.Y.L..EN | 40.85 | 6 | 0.0 |
| I..PS.P.D | 40.85 | 6 | 0.0 |
| I.DKP..P | 40.85 | 6 | 0.0 |
| K.PGPP..P | 40.85 | 6 | 0.0 |
| L.E.Q.Y.F | 40.85 | 6 | 0.0 |
| P.EST..I | 40.85 | 6 | 0.0 |
| P.K.PGPP | 40.85 | 6 | 0.0 |
| PEKKVP | 40.85 | 3 | 0.0 |
| Q..C.CPP | 40.85 | 27 | 0.0 |
| S..DG.C.C | 40.85 | 13 | 0.0 |
| T…MT..W | 40.85 | 6 | 0.0 |
| TSSI.T | 40.85 | 73 | 0.0 |
| V.D.R.K.T | 40.85 | 6 | 0.0 |
| VIAKD | 40.85 | 6 | 0.0 |
| Y..RV.A.N | 40.77 | 7 | 0.0 |
| YK.D.EK | 40.65 | 7 | 0.0 |
| GY.VE..E | 40.54 | 7 | 0.0 |
| GYVVE | 40.54 | 7 | 0.0 |
| KP.GPPPQG | 40.34 | 11 | 0.0 |
| G..GSKG.P | 40.28 | 33 | 0.0 |
| SE.KYK | 40.28 | 7 | 0.0 |
| AT..GSS.T | 40.20 | 10 | 0.0 |
| F.VT.L.E | 39.94 | 6 | 0.0 |
| G..GD.GAK | 39.94 | 21 | 0.0 |
| KGP.V..Q | 39.94 | 22 | 0.0 |
| PP.PEGP | 39.94 | 22 | 0.0 |
| TSSG..EA | 39.94 | 16 | 0.0 |
| GK.GLPG | 39.84 | 45 | 0.0 |
| P.GS.EV | 39.80 | 21 | 0.0 |
| D..SE.KY | 39.71 | 7 | 0.0 |
| Y..V.DTP | 39.71 | 7 | 0.0 |
| KYKE…K | 39.45 | 7 | 0.0 |
| DPPK.P | 39.33 | 7 | 0.0 |
| P…G.SPI | 39.33 | 9 | 0.0 |
| P.GEYW | 39.33 | 26 | 0.0 |
| PG.AG.DG | 39.33 | 32 | 0.0 |
| PHVVP | 39.33 | 26 | 0.0 |
| SG.TCV | 39.33 | 20 | 0.0 |
| V.DVT…V | 39.33 | 22 | 0.0 |
| YEK.K.K | 39.33 | 7 | 0.0 |
| QGPPPQGGN | 39.10 | 6 | 0.0 |
| EK.SL.W | 38.90 | 6 | 0.0 |
| GPPPQGGN | 38.90 | 11 | 0.0 |
| NRYG.S | 38.90 | 6 | 0.0 |
| P.GPPPQG | 38.90 | 11 | 0.0 |
| V.CN.T.G | 38.90 | 30 | 0.0 |
| FRV.A.N | 38.78 | 7 | 0.0 |
| G.C.CAP | 38.78 | 22 | 0.0 |
| P.DDGG | 38.65 | 6 | 0.0 |
| D..F.EPG | 38.58 | 17 | 0.0 |
| G.HG.KG | 38.58 | 37 | 0.0 |
| GP.GL.GFP | 38.58 | 32 | 0.0 |
| TGPI..K | 38.58 | 6 | 0.0 |
| TT..P..ST | 38.49 | 96 | 0.0 |
| P..GVG.G | 38.47 | 29 | 0.0 |
| GG.EIT | 38.44 | 6 | 0.0 |
| A.NK.G.G | 38.32 | 6 | 0.0 |
| RVSA.N | 38.32 | 6 | 0.0 |
| LPG.SG..G | 38.22 | 53 | 0.0 |
| D.G.YT.T | 38.12 | 6 | 0.0 |
| DV.YK.D | 38.12 | 7 | 0.0 |
| I..YVVE | 38.12 | 6 | 0.0 |
| L.EG.EY | 38.12 | 6 | 0.0 |
| N..GVS.P | 38.12 | 6 | 0.0 |
| P..DGGA | 38.12 | 12 | 0.0 |
| YRE..DK | 38.12 | 7 | 0.0 |
| Y.FRV.A | 38.03 | 7 | 0.0 |
| D..PGPPG | 38.01 | 56 | 0.0 |
| PI..PGPP | 37.96 | 10 | 0.0 |
| Q.GPPG.PG | 37.96 | 29 | 0.0 |
| DT..FTS | 37.82 | 11 | 0.0 |
| EPGM…G | 37.82 | 25 | 0.0 |
| TP..DVT | 37.82 | 25 | 0.0 |
| GL.EG..Y | 37.70 | 6 | 0.0 |
| LT.EN..G | 37.70 | 6 | 0.0 |
| KPQGPPPQ | 37.55 | 11 | 0.0 |
| E.GPRG..G | 37.52 | 68 | 0.0 |
| GETC..P | 37.44 | 33 | 0.0 |
| G.KGH.G | 37.40 | 48 | 0.0 |
| GASTAT.S | 37.40 | 8 | 0.0 |
| GIG.P.E | 37.26 | 6 | 0.0 |
| P..PP.KPE | 37.22 | 6 | 0.0 |
| G.TT | 37.20 | 170 | 0.0 |
| AEN.IG | 37.13 | 6 | 0.0 |
| SGASTATNS | 37.06 | 8 | 0.0 |
| SSGASTATN | 37.06 | 8 | 0.0 |
| V.AKN..G | 37.06 | 7 | 0.0 |
| EN..G.S.P | 37.03 | 6 | 0.0 |
| GRP.P…W | 36.76 | 6 | 0.0 |
| PGKPQGP.P | 36.72 | 11 | 0.0 |
| AG..FSP | 36.31 | 16 | 0.0 |
| CLCLC | 36.31 | 20 | 0.0 |
| D.TK.SV | 36.31 | 6 | 0.0 |
| G.YGGDRG | 36.31 | 4 | 0.0 |
| GG.RG.GY | 36.31 | 4 | 0.0 |
| GGY.G.RG | 36.31 | 4 | 0.0 |
| GYGGD.G | 36.31 | 4 | 0.0 |
| H…GPRC | 36.31 | 24 | 0.0 |
| HGP.G.K | 36.31 | 40 | 0.0 |
| IN..G.G.P | 36.31 | 6 | 0.0 |
| IQ..V.A.N | 36.31 | 7 | 0.0 |
| K..ER..PP | 36.31 | 20 | 0.0 |
| K..MT..W | 36.31 | 6 | 0.0 |
| K.KYT..P | 36.31 | 7 | 0.0 |
| KP.P..IT | 36.31 | 20 | 0.0 |
| KYT.T..N | 36.31 | 6 | 0.0 |
| MT..TS.T | 36.31 | 4 | 0.0 |
| N.AG.KT | 36.31 | 6 | 0.0 |
| NR.DSG | 36.31 | 6 | 0.0 |
| P…R.DTG | 36.31 | 8 | 0.0 |
| P…T.SRC | 36.31 | 24 | 0.0 |
| P..NGG.E | 36.31 | 6 | 0.0 |
| P.K.T..DV | 36.31 | 6 | 0.0 |
| P.KIT..K | 36.31 | 25 | 0.0 |
| P.PTTT.S | 36.31 | 11 | 0.0 |
| P.TTPSPPT | 36.31 | 4 | 0.0 |
| P.V.D.TK | 36.31 | 6 | 0.0 |
| P.V.DVT | 36.31 | 6 | 0.0 |
| PIKG.P | 36.31 | 6 | 0.0 |
| PPQPVL | 36.31 | 4 | 0.0 |
| R.DVT..S | 36.31 | 23 | 0.0 |
| RGDK…P | 36.31 | 14 | 0.0 |
| T.F.VT.L | 36.31 | 6 | 0.0 |
| TCPP.F | 36.31 | 20 | 0.0 |
| TH..M.TS | 36.31 | 4 | 0.0 |
| TT.FP.S | 36.31 | 88 | 0.0 |
| TTY.TP | 36.31 | 4 | 0.0 |
| V…V.RTT | 36.31 | 6 | 0.0 |
| YT.TV.N | 36.31 | 6 | 0.0 |
| G.RGF.G.P | 35.91 | 62 | 0.0 |
| DTT | 35.91 | 146 | 0.0 |
| GS.II.Y | 35.52 | 6 | 0.0 |
| TL.W.PP | 35.52 | 6 | 0.0 |
| GE.GPRG | 35.46 | 69 | 0.0 |
| GE.W.DP | 35.40 | 39 | 0.0 |
| V.A.NA.G | 35.40 | 6 | 0.0 |
| KVPE.PK | 35.30 | 3 | 0.0 |
| EYT.VV | 35.01 | 6 | 0.0 |
| G..YYFR | 35.01 | 6 | 0.0 |
| K.S.TL.W | 35.01 | 6 | 0.0 |
| TGL.EG | 35.01 | 6 | 0.0 |
| P..HP.PPG | 34.91 | 26 | 0.0 |
| D.SYP..G | 34.79 | 21 | 0.0 |
| E..TPKT | 34.79 | 23 | 0.0 |
| PPGKPQGPP | 34.79 | 6 | 0.0 |
| PPTTTPSP | 34.79 | 4 | 0.0 |
| V.VLD.P | 34.79 | 6 | 0.0 |
| AEN.FG | 34.66 | 6 | 0.0 |
| P..VPE.PK | 34.58 | 3 | 0.0 |
| G.PGP.G.N | 34.49 | 31 | 0.0 |
| GDR.G.G.G | 34.49 | 7 | 0.0 |
| T.PIK..E | 34.49 | 7 | 0.0 |
| T.T.TP..H | 34.49 | 11 | 0.0 |
| TTPSPPTT | 34.49 | 4 | 0.0 |
| GP.GE.GE | 34.41 | 54 | 0.0 |
| E.PPAKV | 34.29 | 3 | 0.0 |
| TT.PS.PTT | 34.29 | 4 | 0.0 |
| CP..SHY | 34.04 | 3 | 0.0 |
| GDRG.YG | 34.04 | 6 | 0.0 |
| GP..N..VT | 34.04 | 6 | 0.0 |
| I..TR..VT | 34.04 | 6 | 0.0 |
| KRETS | 34.04 | 6 | 0.0 |
| M..SW.PP | 34.04 | 6 | 0.0 |
| TTPPP..TP | 34.04 | 4 | 0.0 |
| VP.KG.P | 34.04 | 6 | 0.0 |
| NE.G.S.P | 33.89 | 7 | 0.0 |
| S.VKY.E | 33.89 | 7 | 0.0 |
| T.TPVT | 33.87 | 94 | 0.0 |
| PPGPPGPRG | 33.83 | 39 | 0.0 |
| CD.GG.C | 33.71 | 26 | 0.0 |
| GPPPQ.G | 33.71 | 11 | 0.0 |
| PGATG..G | 33.71 | 26 | 0.0 |
| T…ITL.W | 33.51 | 6 | 0.0 |
| T..AT | 33.50 | 209 | 0.0 |
| PP.KVPE | 33.44 | 3 | 0.0 |
| IG.VPI | 33.28 | 7 | 0.0 |
| PNQGC | 33.28 | 33 | 0.0 |
| T..FTSI | 33.28 | 8 | 0.0 |
| YE.RV.A | 33.09 | 7 | 0.0 |
| ASTATNSES | 33.01 | 8 | 0.0 |
| KGP.V.A | 32.90 | 27 | 0.0 |
| T..E.RTP | 32.90 | 23 | 0.0 |
| TSS.A.TAT | 32.86 | 8 | 0.0 |
| C…S.C.Y | 32.68 | 31 | 0.0 |
| NAAGV | 32.68 | 6 | 0.0 |
| NCSS.C | 32.68 | 14 | 0.0 |
| PCPL…G | 32.68 | 18 | 0.0 |
| T…LEC.V | 32.68 | 6 | 0.0 |
| TS.L.WT | 32.68 | 6 | 0.0 |
| V.P.G.PQ | 32.68 | 18 | 0.0 |
| W.KCN | 32.68 | 6 | 0.0 |
| RV..VN..G | 32.64 | 7 | 0.0 |
| T…S.S | 32.61 | 245 | 0.0 |
| DG.RR…I | 32.42 | 25 | 0.0 |
| PEI.LA | 32.42 | 10 | 0.0 |
| Y.FR..A | 32.35 | 7 | 0.0 |
| PE.PK..VP | 32.34 | 3 | 0.0 |
| R.DSG.Y | 32.31 | 6 | 0.0 |
| W.PP..D | 32.29 | 11 | 0.0 |
| EPP.ID | 32.27 | 8 | 0.0 |
| G.YTIT | 32.18 | 6 | 0.0 |
| GP.CS..C | 32.18 | 15 | 0.0 |
| QGG.KP.GP | 32.18 | 11 | 0.0 |
| P.DPPG | 32.04 | 6 | 0.0 |
| Y.LEKR | 32.04 | 6 | 0.0 |
| TPL | 31.97 | 190 | 0.0 |
| GP.GL.GP | 31.96 | 83 | 0.0 |
| DNVY..D | 31.77 | 7 | 0.0 |
| EK.KG.M | 31.77 | 7 | 0.0 |
| F.Q.K.NA | 31.77 | 7 | 0.0 |
| KE.TA..T | 31.77 | 14 | 0.0 |
| KN.AG..S | 31.77 | 6 | 0.0 |
| PGPPGPRGP | 31.77 | 31 | 0.0 |
| PGPPGT | 31.77 | 52 | 0.0 |
| R.P.R.GRS | 31.77 | 3 | 0.0 |
| STP.F.S.I | 31.77 | 3 | 0.0 |
| T..VKV.D | 31.77 | 6 | 0.0 |
| TPTT..SA | 31.77 | 7 | 0.0 |
| S…TATNS | 31.68 | 8 | 0.0 |
| D…I..AK | 31.65 | 11 | 0.0 |
| I.G.P.PT | 31.42 | 9 | 0.0 |
| P…APTT | 31.42 | 7 | 0.0 |
| P.G.TG.T | 31.42 | 45 | 0.0 |
| PPPQGGN | 31.42 | 11 | 0.0 |
| T..S..S | 31.37 | 238 | 0.0 |
| PA.TTP | 31.34 | 8 | 0.0 |
| G.DGP.G | 31.32 | 79 | 0.0 |
| PPQGG..PQ | 31.26 | 8 | 0.0 |
| V.E.KVP | 31.26 | 6 | 0.0 |
| C..SSC..C | 31.12 | 21 | 0.0 |
| GY.LE.K | 31.12 | 6 | 0.0 |
| KGSD.W | 31.12 | 6 | 0.0 |
| PPG.PM.H | 31.12 | 3 | 0.0 |
| S..ANTAT | 31.12 | 8 | 0.0 |
| TGDG..C | 31.12 | 23 | 0.0 |
| TSF.V.N | 31.12 | 6 | 0.0 |
| TATNSE | 31.05 | 8 | 0.0 |
| TATNS.S | 31.04 | 8 | 0.0 |
| GG..PQGPP | 30.94 | 17 | 0.0 |
| C.C.NG..C | 30.92 | 16 | 0.0 |
| HTTS | 30.91 | 95 | 0.0 |
| ATNS..ST | 30.82 | 8 | 0.0 |
| D.PG.PE | 30.78 | 6 | 0.0 |
| I.GRP.P | 30.69 | 11 | 0.0 |
| GPRC.C | 30.63 | 27 | 0.0 |
| M.HPP…T | 30.63 | 3 | 0.0 |
| TATNSESST | 30.43 | 8 | 0.0 |
| STATNS..S | 30.42 | 8 | 0.0 |
| A…VKV.D | 30.26 | 6 | 0.0 |
| A.ISG.P | 30.26 | 12 | 0.0 |
| FRVT..N | 30.26 | 6 | 0.0 |
| G..GN.GP | 30.26 | 73 | 0.0 |
| G.D.C.A.L | 30.26 | 6 | 0.0 |
| K.VG.PG | 30.26 | 20 | 0.0 |
| L.WEPP | 30.26 | 6 | 0.0 |
| N..GSK.A | 30.26 | 6 | 0.0 |
| PDG.R..K | 30.26 | 20 | 0.0 |
| TTTPP.TT | 30.26 | 4 | 0.0 |
| T…V..T | 30.17 | 195 | 0.0 |
| G..PQGPPP | 30.12 | 11 | 0.0 |
| SS..ST.TN | 30.12 | 8 | 0.0 |
| P.PPG.P.V | 29.99 | 28 | 0.0 |
| PV.TST | 29.97 | 81 | 0.0 |
| KPQ.PP.PG | 29.95 | 11 | 0.0 |
| DGG..I | 29.92 | 9 | 0.0 |
| KPGPP..P | 29.90 | 19 | 0.0 |
| TP..L.AK | 29.90 | 7 | 0.0 |
| AT.SE.ST | 29.89 | 8 | 0.0 |
| G.C.CA.G | 29.75 | 23 | 0.0 |
| T.TNS.SS | 29.75 | 8 | 0.0 |
| TS…T | 29.71 | 205 | 0.0 |
| H..PG.TE | 29.71 | 22 | 0.0 |
| M.I.W..P | 29.71 | 6 | 0.0 |
| EKKYR | 29.65 | 9 | 0.0 |
| PPGKPQ | 29.65 | 12 | 0.0 |
| E.STP.T | 29.55 | 93 | 0.0 |
| T.STHT | 29.50 | 7 | 0.0 |
| T.TP..HT | 29.50 | 16 | 0.0 |
| VT.L.EG | 29.44 | 7 | 0.0 |
| TTST…S | 29.35 | 90 | 0.0 |
| GGNQS.GP | 29.33 | 5 | 0.0 |
| K.A.R.D.G | 29.33 | 6 | 0.0 |
| D..R.DSG | 29.25 | 6 | 0.0 |
| G.PGS.G.Q | 29.25 | 37 | 0.0 |
| P..PEV..V | 29.25 | 13 | 0.0 |
| S.S.TT..G | 29.17 | 95 | 0.0 |
| P.G.DG..G | 29.16 | 81 | 0.0 |
| PGLPGPPGP | 29.14 | 33 | 0.0 |
| G..GP.GER | 29.05 | 70 | 0.0 |
| KG..G.DG | 29.02 | 72 | 0.0 |
| D.G.Y.V.A | 28.93 | 6 | 0.0 |
| QGGNQ..GP | 28.88 | 11 | 0.0 |
| ATNSESSTT | 28.81 | 8 | 0.0 |
| E.V.DVT | 28.74 | 19 | 0.0 |
| PK.PT.T | 28.74 | 5 | 0.0 |
| T…D.GEY | 28.74 | 6 | 0.0 |
| T..Y.TPS | 28.74 | 7 | 0.0 |
| TAK.KD | 28.74 | 19 | 0.0 |
| EY.F.V | 28.68 | 7 | 0.0 |
| SVSL.W | 28.66 | 6 | 0.0 |
| T…VTL.W | 28.66 | 6 | 0.0 |
| DGSC.C | 28.63 | 19 | 0.0 |
| TSS.TT.ET | 28.63 | 3 | 0.0 |
| G…QGPPP | 28.56 | 25 | 0.0 |
| G.TCV.P | 28.53 | 44 | 0.0 |
| W.K.N…I | 28.53 | 6 | 0.0 |
| I.GKP.P | 28.44 | 11 | 0.0 |
| W.RP..D | 28.41 | 6 | 0.0 |
| G.GEP.E | 28.40 | 7 | 0.0 |
| VTGL.E | 28.40 | 7 | 0.0 |
| VP..PKKP | 28.37 | 3 | 0.0 |
| G..GHPG | 28.33 | 69 | 0.0 |
| L.GPPG..G | 28.25 | 90 | 0.0 |
| A.IQ.PP | 28.24 | 28 | 0.0 |
| D.P.RF.R | 28.24 | 14 | 0.0 |
| E.PTPK | 28.24 | 14 | 0.0 |
| VT..TK.S | 28.24 | 8 | 0.0 |
| V.G.C.C.A | 28.14 | 17 | 0.0 |
| V.N.AGS | 28.14 | 7 | 0.0 |
| G.IGP.G | 28.07 | 83 | 0.0 |
| T..PTST | 28.02 | 24 | 0.0 |
| TATNS | 27.96 | 14 | 0.0 |
| C..PCP.G | 27.93 | 16 | 0.0 |
| GRP.P.V | 27.88 | 20 | 0.0 |
| G.PGFG | 27.71 | 24 | 0.0 |
| P.P.T | 27.68 | 242 | 0.0 |
| PGVG..G | 27.66 | 35 | 0.0 |
| A.R.D.G.Y | 27.64 | 7 | 0.0 |
| GVGEP | 27.63 | 10 | 0.0 |
| TPSPP.T | 27.61 | 7 | 0.0 |
| A.DP..PP | 27.48 | 7 | 0.0 |
| PQGGN..Q | 27.48 | 11 | 0.0 |
| GL.GPPG | 27.46 | 88 | 0.0 |
| A.N.AG.S | 27.23 | 6 | 0.0 |
| A.NR.G.S | 27.23 | 6 | 0.0 |
| AK..F..PG | 27.23 | 6 | 0.0 |
| CKP.C…G | 27.23 | 25 | 0.0 |
| D..PLK.T | 27.23 | 6 | 0.0 |
| D.GKYT | 27.23 | 6 | 0.0 |
| D.PGPP | 27.23 | 9 | 0.0 |
| D.TR.SV | 27.23 | 6 | 0.0 |
| EC.VA..P | 27.23 | 6 | 0.0 |
| EK.KT.Y | 27.23 | 7 | 0.0 |
| EPP.F..K | 27.23 | 3 | 0.0 |
| G…LGYV | 27.23 | 6 | 0.0 |
| G…YGVG | 27.23 | 21 | 0.0 |
| G.PPQG | 27.23 | 11 | 0.0 |
| GDPHY | 27.23 | 12 | 0.0 |
| GKP.P…W | 27.23 | 6 | 0.0 |
| GTP.F…W | 27.23 | 3 | 0.0 |
| HYVVE | 27.23 | 6 | 0.0 |
| KV..L.EG | 27.23 | 6 | 0.0 |
| L.ED..YE | 27.23 | 6 | 0.0 |
| M.V.W..P | 27.23 | 6 | 0.0 |
| PDI.L…N | 27.23 | 7 | 0.0 |
| PG..M.HP | 27.23 | 3 | 0.0 |
| PPP.GT.M | 27.23 | 3 | 0.0 |
| PPPPGKPQG | 27.23 | 6 | 0.0 |
| RR.EEP | 27.23 | 30 | 0.0 |
| RSRTPA | 27.23 | 3 | 0.0 |
| S..LTW.P | 27.23 | 6 | 0.0 |
| TK.S.TL | 27.23 | 6 | 0.0 |
| TT.ETTS | 27.23 | 3 | 0.0 |
| TTPSPP | 27.23 | 6 | 0.0 |
| V…IEG.E | 27.23 | 6 | 0.0 |
| PGP.G..GL | 27.16 | 93 | 0.0 |
| P.PV | 27.04 | 277 | 1.0 |
| G.DG.RG | 27.03 | 56 | 0.0 |
| P.G.PGPPG | 27.02 | 94 | 0.0 |
| G..GQ.GE | 26.97 | 88 | 0.0 |
| A.I.G.P.P | 26.91 | 23 | 0.0 |
| GE.GD.GE | 26.84 | 41 | 0.0 |
| GP.GKP | 26.84 | 90 | 0.0 |
| E..P.KVP | 26.80 | 3 | 0.0 |
| G.CGSGCGG | 26.73 | 20 | 0.0 |
| S.ITTTET | 26.66 | 3 | 0.0 |
| T..KP.PT | 26.58 | 11 | 0.0 |
| GA.CD…G | 26.53 | 15 | 0.0 |
| P.K.E..PP | 26.53 | 22 | 0.0 |
| PQGPPPP | 26.53 | 11 | 0.0 |
| F.GTC.Y | 26.47 | 7 | 0.0 |
| PG.GLPG | 26.47 | 31 | 0.0 |
| G..GK.GP | 26.46 | 83 | 0.0 |
| D..GTC.Y | 26.41 | 7 | 0.0 |
| P.PPGKPQ | 26.41 | 6 | 0.0 |
| PV.AKD | 26.41 | 8 | 0.0 |
| G.C.P.DG | 26.32 | 15 | 0.0 |
| V…DT.TT | 26.32 | 11 | 0.0 |
| DG.RG..G | 26.29 | 58 | 0.0 |
| ATN.D…T | 26.22 | 8 | 0.0 |
| CGS.CG.CG | 26.22 | 23 | 0.0 |
| GCGSGCGGC | 26.16 | 20 | 0.0 |
| KKPE.P | 26.10 | 3 | 0.0 |
| PGGV…A | 26.05 | 20 | 0.0 |
| GDRGG..G | 26.02 | 7 | 0.0 |
| G..PGGV | 25.99 | 19 | 0.0 |
| T..TT | 25.96 | 173 | 0.0 |
| GERGA.G | 25.93 | 49 | 0.0 |
| P.ES.PV | 25.93 | 10 | 0.0 |
| RG..G.QG | 25.90 | 83 | 0.0 |
| PS.TS..TT | 25.83 | 5 | 0.0 |
| G..GK.G.P | 25.81 | 100 | 0.0 |
| G.PGQ.G | 25.80 | 92 | 0.0 |
| R..AIN..G | 25.80 | 6 | 0.0 |
| G.CGSGC | 25.72 | 24 | 0.0 |
| GGS.VT | 25.72 | 14 | 0.0 |
| GLPGP.G | 25.72 | 96 | 0.0 |
| STT.PG.S | 25.72 | 5 | 0.0 |
| G..GLPGP | 25.68 | 93 | 0.0 |
| C.QSSCC.P | 25.67 | 24 | 0.0 |
| TTP | 25.65 | 206 | 0.0 |
| GCGGCGS.C | 25.63 | 20 | 0.0 |
| GSGCGGCGS | 25.63 | 20 | 0.0 |
| I..PGPP | 25.58 | 40 | 0.0 |
| G..GV.GD | 25.49 | 46 | 0.0 |
| D.GK.T.T | 25.42 | 6 | 0.0 |
| P..PPGKP | 25.42 | 21 | 0.0 |
| P.V.PKG | 25.42 | 14 | 0.0 |
| T.TS..SY | 25.42 | 14 | 0.0 |
| GPTG..G | 25.36 | 79 | 0.0 |
| TP..TS.IT | 25.32 | 3 | 0.0 |
| C..SGC.SS | 25.29 | 21 | 0.0 |
| FT..ITTT | 25.29 | 3 | 0.0 |
| G.CCS.G | 25.21 | 25 | 0.0 |
| LQ.PL…Y | 25.21 | 21 | 0.0 |
| G.GLP..T | 25.14 | 18 | 0.0 |
| GS..I.V.W | 25.14 | 3 | 0.0 |
| K.S.SL.W | 25.14 | 6 | 0.0 |
| M…GPPGP | 25.14 | 43 | 0.0 |
| P..YGP.G | 25.14 | 36 | 0.0 |
| G…LKGE | 25.04 | 56 | 0.0 |
| T.SE.ST | 24.98 | 102 | 0.0 |
| AQGV.Q | 24.96 | 22 | 0.0 |
| C.K.CC.SS | 24.96 | 24 | 0.0 |
| CG.CGSGCG | 24.96 | 18 | 0.0 |
| HSTPS..S | 24.96 | 3 | 0.0 |
| KGGCG.CGG | 24.96 | 20 | 0.0 |
| SF..S.TTT | 24.96 | 5 | 0.0 |
| TCQD…C | 24.96 | 22 | 0.0 |
| C.C.N…C | 24.88 | 20 | 0.0 |
| G.IG.PG | 24.84 | 88 | 0.0 |
| T..HSS.GS | 24.81 | 2 | 0.0 |
| H.PPP..PM | 24.76 | 3 | 0.0 |
| IT..W..P | 24.76 | 11 | 0.0 |
| L…DITK | 24.76 | 6 | 0.0 |
| P.LDGG | 24.76 | 12 | 0.0 |
| Q.P.PPGKP | 24.76 | 6 | 0.0 |
| QGP.PPP.K | 24.76 | 6 | 0.0 |
| WG.PC..C | 24.76 | 8 | 0.0 |
| Y..ACQ.A | 24.76 | 3 | 0.0 |
| TTS..TS | 24.74 | 99 | 0.0 |
| PG.CCP | 24.71 | 12 | 0.0 |
| I…GKP.P | 24.64 | 8 | 0.0 |
| PP..S.GF | 24.64 | 19 | 0.0 |
| S.H.DVS | 24.64 | 19 | 0.0 |
| SC.NG..C | 24.64 | 22 | 0.0 |
| GGSKGGCGS | 24.60 | 24 | 0.0 |
| AIN.AG | 24.51 | 6 | 0.0 |
| P.STTT.G | 24.51 | 2 | 0.0 |
| TTT.GLS | 24.51 | 5 | 0.0 |
| WV..NK | 24.51 | 6 | 0.0 |
| K..KAG..L | 24.44 | 21 | 0.0 |
| RG.Y..RG | 24.44 | 21 | 0.0 |
| SSTTSSGAS | 24.44 | 8 | 0.0 |
| STTSSGAST | 24.44 | 8 | 0.0 |
| TTSSGAST | 24.44 | 8 | 0.0 |
| V.P.K.P.V | 24.44 | 17 | 0.0 |
| VTKL..G | 24.39 | 7 | 0.0 |
| G.KGT.G | 24.30 | 45 | 0.0 |
| C…L.VKE | 24.21 | 3 | 0.0 |
| C.N.G.C.P | 24.21 | 22 | 0.0 |
| DGG.KG | 24.21 | 16 | 0.0 |
| K..D.IVV | 24.21 | 6 | 0.0 |
| M…V.AK | 24.21 | 13 | 0.0 |
| P.GPV..D | 24.21 | 6 | 0.0 |
| P.STP.T.F | 24.21 | 2 | 0.0 |
| PARTC | 24.21 | 32 | 0.0 |
| R..VP.KG | 24.21 | 6 | 0.0 |
| R.EQQ..RE | 24.21 | 2 | 0.0 |
| SGCGGCGSG | 24.21 | 20 | 0.0 |
| SSTTSSGA | 24.21 | 8 | 0.0 |
| T..KVT.L | 24.21 | 6 | 0.0 |
| W.PPA..G | 24.21 | 6 | 0.0 |
| WY..D..I | 24.21 | 6 | 0.0 |
| GP.G.PGP | 24.10 | 95 | 0.0 |
| GGCGS.C | 24.07 | 26 | 0.0 |
| C.L.W..P | 24.03 | 6 | 0.0 |
| SST.SSGA | 24.03 | 8 | 0.0 |
| ERRRGR..G | 24.01 | 5 | 0.0 |
| W.KDG.E | 24.01 | 7 | 0.0 |
| G.SG.KG | 24.00 | 50 | 0.0 |
| A..PF.VP | 23.99 | 7 | 0.0 |
| T…T.STH | 23.99 | 5 | 0.0 |
| DP..PP.PP | 23.96 | 6 | 0.0 |
| P.PPGKP | 23.89 | 24 | 0.0 |
| TTPSPV.T | 23.89 | 2 | 0.0 |
| A.V.GKP | 23.83 | 6 | 0.0 |
| CVPAC..S | 23.83 | 21 | 0.0 |
| GCGSSC..S | 23.83 | 21 | 0.0 |
| GV..K.P.V | 23.83 | 16 | 0.0 |
| PG.PE…I | 23.83 | 6 | 0.0 |
| TS.RGR | 23.83 | 21 | 0.0 |
| W.RCN | 23.83 | 6 | 0.0 |
| PPQGG..S | 23.77 | 11 | 0.0 |
| GPAG.PG | 23.72 | 65 | 0.0 |
| SE.STTSSG | 23.72 | 8 | 0.0 |
| G..GL.G.P | 23.70 | 112 | 0.0 |
| G..ILGY | 23.68 | 6 | 0.0 |
| GGCGSCGGS | 23.68 | 20 | 0.0 |
| CGGSKGGCG | 23.60 | 24 | 0.0 |
| F..PGPP | 23.60 | 9 | 0.0 |
| NSD.STTS | 23.60 | 8 | 0.0 |
| P.KVPE | 23.60 | 6 | 0.0 |
| P.Y.P.PP | 23.60 | 21 | 0.0 |
| TT.TTSH | 23.60 | 3 | 0.0 |
| A.NAAG | 23.56 | 6 | 0.0 |
| T..TT.TP | 23.52 | 93 | 0.0 |
| S.CCKPC | 23.52 | 24 | 0.0 |
| G..GL.G.K | 23.51 | 94 | 0.0 |
| SE.ST.S.G | 23.46 | 8 | 0.0 |
| PP..P.V.D | 23.45 | 8 | 0.0 |
| GFQG..G | 23.38 | 70 | 0.0 |
| SCGGSKGGC | 23.38 | 24 | 0.0 |
| ET..LP.ST | 23.34 | 2 | 0.0 |
| GSKGGCGSC | 23.34 | 24 | 0.0 |
| S.P.V..DP | 23.34 | 6 | 0.0 |
| SE.STT.Y | 23.34 | 2 | 0.0 |
| T…K.APT | 23.34 | 6 | 0.0 |
| TPMAH | 23.34 | 3 | 0.0 |
| G..GEKG | 23.31 | 92 | 0.0 |
| A.NE.G.S | 23.28 | 7 | 0.0 |
| E.G.PG..G | 23.26 | 95 | 0.0 |
| TGAC.C | 23.26 | 16 | 0.0 |
| PG..GE.G | 23.26 | 95 | 0.0 |
| G..GL.GP | 23.22 | 95 | 0.0 |
| T.T.SHST | 23.20 | 3 | 0.0 |
| VPDTP | 23.20 | 9 | 0.0 |
| SKGGCGSC | 23.16 | 24 | 0.0 |
| T.SSG..TA | 23.16 | 8 | 0.0 |
| GLPG..G | 23.12 | 97 | 0.0 |
| SS.ASTA | 23.12 | 22 | 0.0 |
| KG.RG..G | 23.12 | 99 | 0.0 |
| G.PGLPG | 23.11 | 95 | 0.0 |
| DI..AKK | 23.10 | 7 | 0.0 |
| GSCGGSKGG | 23.10 | 24 | 0.0 |
| HR.SQ..S | 23.10 | 20 | 0.0 |
| PTT.F..S | 23.10 | 6 | 0.0 |
| TPSPV.T.S | 23.10 | 2 | 0.0 |
| VP.K.VP | 23.10 | 3 | 0.0 |
| GCG.CGGS | 23.07 | 21 | 0.0 |
| G.VG.PG | 23.06 | 101 | 0.0 |
| E.LYK…E | 23.04 | 7 | 0.0 |
| W..PHY | 23.04 | 14 | 0.0 |
| P.G.KG..G | 23.04 | 94 | 0.0 |
| G..GPPG.P | 23.01 | 96 | 0.0 |
| NSESSTTSS | 22.99 | 8 | 0.0 |
| TNSESS.TS | 22.99 | 8 | 0.0 |
| G..GE.G.P | 22.99 | 95 | 0.0 |
| CGSCGGSKG | 22.96 | 24 | 0.0 |
| S.C.QSSCC | 22.96 | 23 | 0.0 |
| SGC.SSCC | 22.96 | 21 | 0.0 |
| A.V.G.P.P | 22.93 | 19 | 0.0 |
| V.TTSTTS | 22.93 | 2 | 0.0 |
| P.G.PG..G | 22.91 | 99 | 0.0 |
| KG.EG..G | 22.91 | 73 | 0.0 |
| S..STTS.G | 22.89 | 10 | 0.0 |
| TLSPA..T | 22.89 | 4 | 0.0 |
| PP.KPE | 22.87 | 27 | 0.0 |
| E..TL.W | 22.83 | 29 | 0.0 |
| G.DG.PG | 22.82 | 93 | 0.0 |
| P.TTPSP | 22.81 | 8 | 0.0 |
| TNS.S..TS | 22.78 | 8 | 0.0 |
| G..GE.GP | 22.71 | 95 | 0.0 |
| CE..E..CE | 22.69 | 35 | 0.0 |
| DA.EYT | 22.69 | 6 | 0.0 |
| FDF.G.C | 22.69 | 3 | 0.0 |
| G..FD..GT | 22.69 | 3 | 0.0 |
| G.YTI.A | 22.69 | 6 | 0.0 |
| GC.CD..F | 22.69 | 3 | 0.0 |
| GGCGS.G | 22.69 | 24 | 0.0 |
| RRD.R..P | 22.69 | 25 | 0.0 |
| SAPT.Q | 22.69 | 25 | 0.0 |
| T…VTRN | 22.69 | 6 | 0.0 |
| T..GVS.ES | 22.69 | 2 | 0.0 |
| T.VTK.S | 22.69 | 6 | 0.0 |
| G..GP.GPP | 22.68 | 95 | 0.0 |
| G..GP.GE | 22.68 | 98 | 0.0 |
| G..GLPG | 22.67 | 96 | 0.0 |
| TNS.SST.S | 22.62 | 8 | 0.0 |
| GPPG.PG | 22.62 | 95 | 0.0 |
| C…C.C.N | 22.61 | 16 | 0.0 |
| C.QSSCC | 22.58 | 24 | 0.0 |
| GRRSK.E | 22.55 | 5 | 0.0 |
| KERRRGRK | 22.55 | 5 | 0.0 |
| KKERRRG | 22.55 | 5 | 0.0 |
| RGRRS..ER | 22.55 | 5 | 0.0 |
| RRG.RSK.E | 22.55 | 5 | 0.0 |
| RRSKKE..R | 22.55 | 5 | 0.0 |
| RSKKE.R.G | 22.55 | 5 | 0.0 |
| SKKER.R.R | 22.55 | 5 | 0.0 |
| G.PG.KG | 22.53 | 95 | 0.0 |
| G.LG.PG | 22.49 | 102 | 0.0 |
| PG.KG..G | 22.48 | 101 | 0.0 |
| S.GCGSSC | 22.45 | 21 | 0.0 |
| SPV.TT.TT | 22.43 | 2 | 0.0 |
| STT.GP.T | 22.43 | 2 | 0.0 |
| T..TS.PVS | 22.43 | 2 | 0.0 |
| CC.PC…S | 22.41 | 25 | 0.0 |
| G..G.KGE | 22.40 | 95 | 0.0 |
| G.QG.PG | 22.36 | 95 | 0.0 |
| G..CS..DG | 22.34 | 9 | 0.0 |
| TGS.HT | 22.34 | 8 | 0.0 |
| Y.PGPP | 22.34 | 8 | 0.0 |
| EIKPS | 22.28 | 6 | 0.0 |
| EPPSF | 22.28 | 3 | 0.0 |
| M…W.PP | 22.28 | 6 | 0.0 |
| PVCC..P | 22.28 | 24 | 0.0 |
| SM.V.W | 22.28 | 6 | 0.0 |
| TTSSG.S | 22.28 | 10 | 0.0 |
| P.TTPSPV | 22.19 | 2 | 0.0 |
| S..GS.HTT | 22.19 | 2 | 0.0 |
| S.K.APT | 22.19 | 30 | 0.0 |
| SSCCV..C | 22.19 | 23 | 0.0 |
| V.TTSTTSA | 22.19 | 2 | 0.0 |
| RG..G..GP | 22.11 | 115 | 0.0 |
| G..GP.G.P | 22.11 | 109 | 0.0 |
| G.GKPS | 22.10 | 8 | 0.0 |
| HTTS…S | 22.10 | 35 | 0.0 |
| S.GST..T | 22.09 | 85 | 0.0 |
| SPC.R..C | 22.04 | 17 | 0.0 |
| VPG.V…A | 22.04 | 16 | 0.0 |
| GPQGP | 21.94 | 100 | 0.0 |
| ST.SS.A.T | 21.92 | 10 | 0.0 |
| KG..GP.G | 21.92 | 95 | 0.0 |
| T..P.STT | 21.92 | 16 | 0.0 |
| G.PGP.GP | 21.90 | 95 | 0.0 |
| P.G.AG..G | 21.83 | 118 | 0.0 |
| D.PGPV | 21.78 | 6 | 0.0 |
| GVG.P.A | 21.78 | 26 | 0.0 |
| P.TTLS.A | 21.78 | 2 | 0.0 |
| T..VKA.E | 21.78 | 6 | 0.0 |
| T.T.HF..S | 21.78 | 2 | 0.0 |
| W.RVN | 21.78 | 6 | 0.0 |
| GP.G..GP | 21.77 | 112 | 0.0 |
| PGPPG..G | 21.75 | 95 | 0.0 |
| V..SE.STL | 21.71 | 5 | 0.0 |
| G.FHC.C | 21.68 | 21 | 0.0 |
| PGPP.N | 21.67 | 7 | 0.0 |
| A.N.AG..S | 21.64 | 6 | 0.0 |
| A.V.GRP | 21.64 | 7 | 0.0 |
| H.G.TG..G | 21.64 | 31 | 0.0 |
| P.CS.SSC | 21.64 | 34 | 0.0 |
| G.NG.PG | 21.64 | 75 | 0.0 |
| G.PGPPG | 21.63 | 95 | 0.0 |
| TS.PG.T.S | 21.61 | 2 | 0.0 |
| G..GFPG | 21.56 | 106 | 0.0 |
| CG.YN.D | 21.56 | 3 | 0.0 |
| NSE.ST.S | 21.56 | 10 | 0.0 |
| S.T.G.HG | 21.56 | 15 | 0.0 |
| PG.VG..G | 21.53 | 100 | 0.0 |
| TT..P.STT | 21.53 | 13 | 0.0 |
| G.PGV.G | 21.51 | 106 | 0.0 |
| S.T.A.GTS | 21.45 | 2 | 0.0 |
| STTPV.SN | 21.45 | 2 | 0.0 |
| G..GVPG | 21.45 | 109 | 0.0 |
| QS.CC.P.C | 21.44 | 38 | 0.0 |
| TVT.T.TPT | 21.43 | 4 | 0.0 |
| TST.S…T | 21.40 | 91 | 0.0 |
| K.PEVP | 21.37 | 3 | 0.0 |
| T.SE.STP | 21.37 | 16 | 0.0 |
| EGEEDQN.P | 21.36 | 5 | 0.0 |
| STTF…P | 21.36 | 2 | 0.0 |
| PQGPP.PP | 21.31 | 19 | 0.0 |
| S..YVLE.Q | 21.31 | 5 | 0.0 |
| G.PGL.G | 21.27 | 102 | 0.0 |
| G..GPPG | 21.27 | 116 | 0.0 |
| G..GP.GP | 21.24 | 98 | 0.0 |
| ASTLSTT.V | 21.18 | 2 | 0.0 |
| D.E.PP.V | 21.18 | 14 | 0.0 |
| E.STPS.G | 21.18 | 8 | 0.0 |
| KK.RG.RSK | 21.18 | 5 | 0.0 |
| KRRGR..KK | 21.18 | 5 | 0.0 |
| S.PVST..V | 21.18 | 2 | 0.0 |
| TT..TS.ST | 21.18 | 5 | 0.0 |
| TTF.S.P.S | 21.18 | 2 | 0.0 |
| VP.CCQ | 21.18 | 24 | 0.0 |
| VTPTPTPT | 21.18 | 4 | 0.0 |
| KG.KG.P | 21.15 | 82 | 0.0 |
| G..GAKG | 21.13 | 81 | 0.0 |
| I.G.PGP | 21.12 | 79 | 0.0 |
| P.G.QG..G | 21.12 | 92 | 0.0 |
| TTS.ST.S | 21.08 | 9 | 0.0 |
| GPPG..G | 21.04 | 101 | 0.0 |
| G.RG.YG | 21.04 | 27 | 0.0 |
| TSH.T…T | 21.04 | 3 | 0.0 |
| SPPGK | 21.02 | 12 | 0.0 |
| T..TTP.DT | 21.02 | 2 | 0.0 |
| T.T…S | 20.99 | 224 | 0.0 |
| S.T.SW..P | 20.99 | 7 | 0.0 |
| E.A.F..EL | 20.95 | 6 | 0.0 |
| K.L.S..KY | 20.95 | 13 | 0.0 |
| PEVT..T | 20.95 | 6 | 0.0 |
| V.GKP.P | 20.95 | 18 | 0.0 |
| S.PTT.E.T | 20.88 | 2 | 0.0 |
| T..PGSTT | 20.88 | 2 | 0.0 |
| T.SAPTT | 20.88 | 7 | 0.0 |
| PG..GP.G | 20.87 | 101 | 0.0 |
| T.TPTPTGT | 20.87 | 4 | 0.0 |
| DLGQPYS | 20.85 | 5 | 0.0 |
| LGQ.YS..V | 20.85 | 5 | 0.0 |
| LPD.G.P.S | 20.85 | 5 | 0.0 |
| PD..QPYSS | 20.85 | 5 | 0.0 |
| PYSSA.Y.L | 20.85 | 5 | 0.0 |
| QPYSSA..S | 20.85 | 5 | 0.0 |
| YSSAVY.L | 20.85 | 5 | 0.0 |
| C.P.CS.SS | 20.84 | 33 | 0.0 |
| SE..TL.TT | 20.84 | 2 | 0.0 |
| T.S..T | 20.82 | 201 | 0.0 |
| TTSAPTTS | 20.82 | 2 | 0.0 |
| W..DG…K | 20.82 | 6 | 0.0 |
| G..GQ.G.P | 20.79 | 93 | 0.0 |
| G.RGE.G | 20.79 | 93 | 0.0 |
| V.A.N..G | 20.78 | 7 | 0.0 |
| G.PG..GP | 20.77 | 96 | 0.0 |
| DQNPP.P.L | 20.75 | 5 | 0.0 |
| R.EGEED.N | 20.75 | 5 | 0.0 |
| STT.VDS | 20.75 | 2 | 0.0 |
| GPPGPPGP | 20.72 | 90 | 0.0 |
| PG.DG..G | 20.71 | 93 | 0.0 |
| TPTPT.T | 20.71 | 4 | 0.0 |
| I.G.P.P.V | 20.66 | 6 | 0.0 |
| PSPVPTT.T | 20.63 | 2 | 0.0 |
| V..PISG | 20.63 | 25 | 0.0 |
| H..PGST.T | 20.61 | 2 | 0.0 |
| R..RKEGEE | 20.61 | 5 | 0.0 |
| RGR.E.EED | 20.61 | 5 | 0.0 |
| ESSTVS | 20.57 | 8 | 0.0 |
| T.TVT.TPT | 20.55 | 4 | 0.0 |
| T.TPTGT | 20.55 | 4 | 0.0 |
| GP.GR.G | 20.52 | 87 | 0.0 |
| STLSTTPV | 20.52 | 2 | 0.0 |
| T..EGTS.P | 20.52 | 2 | 0.0 |
| TT.H..PS | 20.51 | 6 | 0.0 |
| G.PGP.G | 20.46 | 101 | 0.0 |
| GP.GP.G | 20.44 | 96 | 0.0 |
| CC.SGC.S | 20.42 | 19 | 0.0 |
| CCCSS..G | 20.42 | 19 | 0.0 |
| CP.N..Y | 20.42 | 29 | 0.0 |
| ECEVS | 20.42 | 6 | 0.0 |
| LS.TP..TS | 20.42 | 2 | 0.0 |
| P.STTTP | 20.42 | 4 | 0.0 |
| SPG.C…T | 20.42 | 18 | 0.0 |
| SSPTT.E | 20.42 | 2 | 0.0 |
| T.LTE..E | 20.42 | 6 | 0.0 |
| TTPSPV | 20.42 | 4 | 0.0 |
| G.PGF.G | 20.38 | 89 | 0.0 |
| PG.PG..G | 20.36 | 97 | 0.0 |
| LEVVEP.V | 20.35 | 5 | 0.0 |
| LLEVVE..V | 20.35 | 5 | 0.0 |
| H..G.C.C | 20.33 | 14 | 0.0 |
| G.PGM.G | 20.33 | 82 | 0.0 |
| TT..PTPT | 20.30 | 4 | 0.0 |
| N..G.G.P | 20.26 | 10 | 0.0 |
| F.K.KGK | 20.25 | 8 | 0.0 |
| DP.CPRL.R | 20.22 | 5 | 0.0 |
| GPPGP.G | 20.21 | 95 | 0.0 |
| PT..EGT | 20.20 | 17 | 0.0 |
| EEDQN..CP | 20.17 | 5 | 0.0 |
| V..EN..G | 20.16 | 10 | 0.0 |
| TST…T | 20.13 | 129 | 0.0 |
| PGAGIP | 20.13 | 16 | 0.0 |
| TVTPTPTP | 20.11 | 4 | 0.0 |
| P.G.RG..G | 20.08 | 93 | 0.0 |
| TPSGYLE.P | 20.08 | 5 | 0.0 |
| EVVEPEVL | 20.06 | 5 | 0.0 |
| F…STTS | 20.06 | 8 | 0.0 |
| LAL.VD..K | 20.06 | 5 | 0.0 |
| RRRGRKEG | 20.06 | 5 | 0.0 |
| S.EAS.L.T | 20.06 | 2 | 0.0 |
| PTGTQ | 20.04 | 23 | 0.0 |
| PTTSTTSAP | 20.03 | 2 | 0.0 |
| PTPTPTGT | 19.99 | 4 | 0.0 |
| AL.VD.T.K | 19.97 | 2 | 0.0 |
| D.D.TK.DQ | 19.97 | 2 | 0.0 |
| EQQLR.E | 19.97 | 2 | 0.0 |
| G..GV.G.P | 19.97 | 101 | 0.0 |
| KG.SG..G | 19.97 | 61 | 0.0 |
| LSE.STT | 19.97 | 2 | 0.0 |
| SAP.S.TT | 19.97 | 2 | 0.0 |
| SYSTPS | 19.97 | 5 | 0.0 |
| G..GIPG | 19.96 | 110 | 0.0 |
| T.TPI..TT | 19.95 | 4 | 0.0 |
| G.PGT.G | 19.94 | 94 | 0.0 |
| DRC.STPSG | 19.94 | 5 | 0.0 |
| EA.TL..TP | 19.91 | 2 | 0.0 |
| TSTTSAPTT | 19.91 | 2 | 0.0 |
| TTSTTSAPT | 19.91 | 2 | 0.0 |
| APTTSTTSA | 19.88 | 2 | 0.0 |
| G.CVN..G | 19.87 | 23 | 0.0 |
| G.PGK.G | 19.86 | 95 | 0.0 |
| KGEP.V | 19.86 | 35 | 0.0 |
| P..TVTW | 19.86 | 6 | 0.0 |
| STTPVD | 19.86 | 2 | 0.0 |
| T.P.T.T.F | 19.86 | 13 | 0.0 |
| G.PG.PG | 19.83 | 112 | 0.0 |
| DS.QP.GSS | 19.83 | 5 | 0.0 |
| GSSFYAL | 19.83 | 5 | 0.0 |
| PYGSS..A | 19.83 | 5 | 0.0 |
| QP.SCQP.G | 19.83 | 5 | 0.0 |
| QPYGSS..A | 19.83 | 5 | 0.0 |
| STPSSC..Q | 19.83 | 5 | 0.0 |
| YGSSF.A | 19.83 | 5 | 0.0 |
| C..AW..P | 19.80 | 6 | 0.0 |
| E…KGSD | 19.80 | 6 | 0.0 |
| QQ.RREQ | 19.80 | 2 | 0.0 |
| RREQQ..R | 19.80 | 2 | 0.0 |
| SS.C.SSC | 19.80 | 23 | 0.0 |
| V.GRP.P | 19.80 | 6 | 0.0 |
| LG.ALDVD | 19.78 | 5 | 0.0 |
| PSG.LE.PD | 19.78 | 5 | 0.0 |
| SG.LE.PD | 19.78 | 5 | 0.0 |
| Y.GLA.DVD | 19.78 | 5 | 0.0 |
| G..GI.G.P | 19.77 | 76 | 0.0 |
| S..TT.TTS | 19.76 | 5 | 0.0 |
| C.SC.GS | 19.70 | 27 | 0.0 |
| CP.GS.G | 19.67 | 17 | 0.0 |
| STTSGL | 19.67 | 2 | 0.0 |
| T…V..KW | 19.67 | 9 | 0.0 |
| TSA.T..TT | 19.64 | 4 | 0.0 |
| G.TG.PG | 19.63 | 85 | 0.0 |
| E.C.L.W | 19.62 | 13 | 0.0 |
| T.TGTQ.P | 19.61 | 4 | 0.0 |
| C..W.TP | 19.55 | 21 | 0.0 |
| GK.RRGRR | 19.55 | 5 | 0.0 |
| LDVDR…D | 19.55 | 5 | 0.0 |
| LYK..W | 19.55 | 7 | 0.0 |
| PPRF.R | 19.55 | 14 | 0.0 |
| PQ.STT | 19.55 | 16 | 0.0 |
| T.TE..TP | 19.55 | 22 | 0.0 |
| VDR.KK..E | 19.55 | 5 | 0.0 |
| STP.TS | 19.54 | 90 | 0.0 |
| G.KG..G | 19.49 | 129 | 0.0 |
| G.C.N..GS | 19.48 | 25 | 0.0 |
| T.STTS.P | 19.47 | 2 | 0.0 |
| I.W.K.N | 19.45 | 6 | 0.0 |
| ITKDS | 19.45 | 6 | 0.0 |
| TD..T.PG | 19.45 | 15 | 0.0 |
| TSAPTTSTT | 19.45 | 2 | 0.0 |
| STTSAPTTS | 19.41 | 2 | 0.0 |
| GE.GE.G | 19.40 | 89 | 0.0 |
| ST.TT..P | 19.40 | 10 | 0.0 |
| TTFY..P | 19.36 | 2 | 0.0 |
| TTTTVTP | 19.36 | 4 | 0.0 |
| CLCPP | 19.34 | 26 | 0.0 |
| G..EESTT | 19.32 | 2 | 0.0 |
| CL..P.SCQ | 19.32 | 5 | 0.0 |
| CQP.GSSF | 19.32 | 5 | 0.0 |
| EQPDS.QP | 19.32 | 5 | 0.0 |
| LEQPDS.Q | 19.32 | 5 | 0.0 |
| SCLEQ..SC | 19.32 | 5 | 0.0 |
| SCQPYG.S | 19.32 | 5 | 0.0 |
| SSC.E.P.S | 19.32 | 5 | 0.0 |
| GQP.SS.V | 19.29 | 5 | 0.0 |
| SAPTTSTTS | 19.29 | 2 | 0.0 |
| SSA.YSLE | 19.29 | 5 | 0.0 |
| G..G.KG | 19.25 | 100 | 0.0 |
| A…W.KP | 19.22 | 6 | 0.0 |
| DAGKY | 19.22 | 6 | 0.0 |
| PF.VP..P | 19.22 | 6 | 0.0 |
| YSTPS..LE | 19.21 | 5 | 0.0 |
| A.TTST.S | 19.21 | 2 | 0.0 |
| RG.AG..G | 19.19 | 85 | 0.0 |
| HS.PGST | 19.19 | 2 | 0.0 |
| DRSY.TP | 19.16 | 2 | 0.0 |
| P.TTLSP | 19.16 | 2 | 0.0 |
| DEKGPEV | 19.14 | 5 | 0.0 |
| P.PT.TW | 19.13 | 6 | 0.0 |
| G.CIN..G | 19.11 | 11 | 0.0 |
| GEEDQN | 19.11 | 5 | 0.0 |
| S..VTTS.E | 19.11 | 2 | 0.0 |
| ST..HSS.G | 19.11 | 2 | 0.0 |
| G..GMPG | 19.10 | 82 | 0.0 |
| P.CPRLSR | 19.09 | 5 | 0.0 |
| GGCGS | 19.09 | 26 | 0.0 |
| S..ALEEK | 19.08 | 5 | 0.0 |
| SS.YAL..K | 19.08 | 5 | 0.0 |
| AKLT..E | 19.06 | 6 | 0.0 |
| D..YSTPSS | 19.06 | 5 | 0.0 |
| G.KGEP | 19.06 | 90 | 0.0 |
| GKG.KRR.R | 19.06 | 5 | 0.0 |
| GR.EGEED | 19.06 | 5 | 0.0 |
| KGKGKKRR | 19.06 | 5 | 0.0 |
| KKGKG.KR | 19.06 | 5 | 0.0 |
| PT.T.SEG | 19.06 | 2 | 0.0 |
| Y.IEM | 19.06 | 9 | 0.0 |
| PGPPG | 19.05 | 104 | 0.0 |
| PG.PG.PG | 19.02 | 95 | 0.0 |
| DTSTP.T | 19.02 | 2 | 0.0 |
| GSTPL…P | 19.02 | 2 | 0.0 |
| RTKKD…E | 19.02 | 2 | 0.0 |
| TTFHS | 19.02 | 2 | 0.0 |
| CYSTPSG | 19.00 | 5 | 0.0 |
| RCYSTPSG | 19.00 | 5 | 0.0 |
| WG..CE | 19.00 | 30 | 0.0 |
| C.CAPG | 18.98 | 35 | 0.0 |
| PSHP.T | 18.98 | 11 | 0.0 |
| PPCP.LSR | 18.97 | 5 | 0.0 |
| CPRLSRE.L | 18.96 | 5 | 0.0 |
| KG..G..G | 18.96 | 136 | 0.0 |
| CYSTPS.CL | 18.94 | 5 | 0.0 |
| ESTTF | 18.94 | 2 | 0.0 |
| RCYSTPS.C | 18.94 | 5 | 0.0 |
| STT.A..TS | 18.91 | 2 | 0.0 |
| A..TS.PTS | 18.88 | 4 | 0.0 |
| TPI.TTT | 18.86 | 4 | 0.0 |
| E.P.PP.K | 18.85 | 9 | 0.0 |
| F.L.VGEIE | 18.83 | 5 | 0.0 |
| GF.LDVG.I | 18.83 | 5 | 0.0 |
| HVGFS..VG | 18.83 | 5 | 0.0 |
| PSSCL..PD | 18.83 | 5 | 0.0 |
| VG..LDVGE | 18.83 | 5 | 0.0 |
| QY.GLA.DV | 18.82 | 5 | 0.0 |
| SV..SW..P | 18.78 | 6 | 0.0 |
| DS.DRC.ST | 18.77 | 5 | 0.0 |
| TTP.D..T | 18.76 | 2 | 0.0 |
| LSR.LLDEK | 18.75 | 5 | 0.0 |
| RLSRE.LDE | 18.75 | 5 | 0.0 |
| G.KGA.G | 18.75 | 87 | 0.0 |
| PCPRLSR | 18.73 | 5 | 0.0 |
| S.TS.GVS | 18.72 | 10 | 0.0 |
| VPKK…P | 18.72 | 6 | 0.0 |
| TS..S.GST | 18.70 | 4 | 0.0 |
| E.STT..SS | 18.69 | 2 | 0.0 |
| T.T.T.TPI | 18.69 | 4 | 0.0 |
| G..GA.G.P | 18.67 | 100 | 0.0 |
| G.KGS.G | 18.66 | 81 | 0.0 |
| PGPPG.P | 18.66 | 103 | 0.0 |
| DGGAP | 18.66 | 7 | 0.0 |
| T…ASSSP | 18.66 | 3 | 0.0 |
| SD..YR | 18.65 | 17 | 0.0 |
| C.P.C..C | 18.63 | 33 | 0.0 |
| P..SL.PK | 18.63 | 25 | 0.0 |
| G.KGD.G | 18.63 | 95 | 0.0 |
| EEKHV..SL | 18.61 | 5 | 0.0 |
| EKHVG..L | 18.61 | 5 | 0.0 |
| LEEKH.G.S | 18.61 | 5 | 0.0 |
| GLALDV.R | 18.60 | 5 | 0.0 |
| IEKKGKG.K | 18.60 | 5 | 0.0 |
| VVEPEV.Q | 18.60 | 5 | 0.0 |
| P.PTGTQTP | 18.58 | 4 | 0.0 |
| PG.LG..G | 18.58 | 91 | 0.0 |
| T.TGT.TPT | 18.58 | 4 | 0.0 |
| TT.HS.P | 18.55 | 7 | 0.0 |
| WG.P.Y | 18.52 | 14 | 0.0 |
| RELLDE..P | 18.51 | 5 | 0.0 |
| YSTPS.C | 18.49 | 5 | 0.0 |
| AW.KP | 18.48 | 9 | 0.0 |
| V.DTP | 18.44 | 107 | 0.0 |
| PG.RG..G | 18.43 | 99 | 0.0 |
| QDS.DRCYS | 18.40 | 5 | 0.0 |
| N..GT.S | 18.38 | 107 | 0.0 |
| ED..PS.PR | 18.38 | 5 | 0.0 |
| EEDQDPS.P | 18.38 | 5 | 0.0 |
| PYR.AFY | 18.38 | 5 | 0.0 |
| S.PRLSRE | 18.38 | 5 | 0.0 |
| PEV.QDS.D | 18.38 | 5 | 0.0 |
| AVY.LE.Q | 18.37 | 5 | 0.0 |
| EQYLG.ALD | 18.37 | 5 | 0.0 |
| GYLELPD | 18.37 | 5 | 0.0 |
| LE.QYL.L | 18.37 | 5 | 0.0 |
| SAVYS.E.Q | 18.37 | 5 | 0.0 |
| SLEEQY..L | 18.37 | 5 | 0.0 |
| VYS.EEQ | 18.37 | 5 | 0.0 |
| Y.LEEQ..G | 18.37 | 5 | 0.0 |
| PTGTQT.T | 18.36 | 4 | 0.0 |
| SLDRCYSTP | 18.33 | 5 | 0.0 |
| LDRCYSTPS | 18.32 | 5 | 0.0 |
| SRE.LDEK | 18.27 | 5 | 0.0 |
| EVLQDSLDR | 18.23 | 5 | 0.0 |
| TT..TP..T | 18.22 | 21 | 0.0 |
| A.SSPTT | 18.15 | 2 | 0.0 |
| CCKP.C | 18.15 | 45 | 0.0 |
| CP.GT.G | 18.15 | 22 | 0.0 |
| DVDRIK.D | 18.15 | 3 | 0.0 |
| FY.LE.K.V | 18.15 | 5 | 0.0 |
| G.CGCS | 18.15 | 24 | 0.0 |
| G.SE.STT | 18.15 | 2 | 0.0 |
| GPPGPP | 18.15 | 102 | 0.0 |
| GST.TT..P | 18.15 | 2 | 0.0 |
| IEKK.KGK | 18.15 | 5 | 0.0 |
| KGK.RRGR | 18.15 | 5 | 0.0 |
| LSRELL.VV | 18.15 | 5 | 0.0 |
| P…GAGIP | 18.15 | 16 | 0.0 |
| P..TETT | 18.15 | 6 | 0.0 |
| P.C.R..CH | 18.15 | 12 | 0.0 |
| P.K.PGP | 18.15 | 12 | 0.0 |
| P.LAV.P | 18.15 | 32 | 0.0 |
| P.RSTTS | 18.15 | 2 | 0.0 |
| PRLSRE.LD | 18.15 | 5 | 0.0 |
| Q.P.CPRLS | 18.15 | 5 | 0.0 |
| RELLEVVE | 18.15 | 5 | 0.0 |
| RLSRELLEV | 18.15 | 5 | 0.0 |
| S.GSTP.T | 18.15 | 4 | 0.0 |
| SR.LLEV.E | 18.15 | 5 | 0.0 |
| T…C..TW | 18.15 | 6 | 0.0 |
| T.G.SE.ST | 18.15 | 2 | 0.0 |
| T.R.SY..S | 18.15 | 14 | 0.0 |
| T.SEGST | 18.15 | 2 | 0.0 |
| TPTPT.TQT | 18.15 | 4 | 0.0 |
| TS..VE..T | 18.15 | 8 | 0.0 |
| TSTP.T | 18.15 | 13 | 0.0 |
| TT.GR.EE | 18.15 | 2 | 0.0 |
| TTS.L..ES | 18.15 | 2 | 0.0 |
| V.TV..AN | 18.15 | 9 | 0.0 |
| YALEEKH | 18.15 | 5 | 0.0 |
| E.EEDQ.P | 18.08 | 5 | 0.0 |
| E..PEVLQD | 18.04 | 5 | 0.0 |
| Y.TPS.C | 18.04 | 5 | 0.0 |
| STPSG.LE | 18.04 | 5 | 0.0 |
| L.EG..Y | 17.99 | 9 | 0.0 |
| EEQYLGL | 17.94 | 5 | 0.0 |
| ELP.LGQP | 17.94 | 5 | 0.0 |
| TQTPT.TP | 17.94 | 4 | 0.0 |
| YL..PD.GQ | 17.94 | 5 | 0.0 |
| DVGEIEK | 17.94 | 5 | 0.0 |
| GEIEK..K | 17.94 | 5 | 0.0 |
| LDVGEIE | 17.94 | 5 | 0.0 |
| SLD.G.IE | 17.94 | 5 | 0.0 |
| TPSSC..Q | 17.94 | 5 | 0.0 |
| DSCQP..S | 17.94 | 5 | 0.0 |
| C…CQC | 17.93 | 28 | 0.0 |
| EVEEDQ..S | 17.93 | 5 | 0.0 |
| G.PGD.G | 17.92 | 88 | 0.0 |
| VG.AVD.DE | 17.91 | 5 | 0.0 |
| G.QG..G | 17.90 | 105 | 0.0 |
| D.GEYT | 17.85 | 6 | 0.0 |
| G.RG.PG | 17.84 | 96 | 0.0 |
| SS.GST..T | 17.84 | 5 | 0.0 |
| TGTQTP..T | 17.84 | 4 | 0.0 |
| VLQDSLDRC | 17.84 | 5 | 0.0 |
| ELLDEK.PE | 17.81 | 5 | 0.0 |
| LLDEK.PEV | 17.81 | 5 | 0.0 |
| S.PVTTS | 17.76 | 3 | 0.0 |
| EKKGKG.K | 17.73 | 5 | 0.0 |
| KD.EEEEDQ | 17.73 | 5 | 0.0 |
| KH.GFSLD | 17.72 | 5 | 0.0 |
| E.T.SCQPY | 17.71 | 5 | 0.0 |
| PRLSRE.LE | 17.69 | 5 | 0.0 |
| LQDSLDRCY | 17.68 | 5 | 0.0 |
| C.C.H…C | 17.68 | 17 | 0.0 |
| SC.SCG | 17.68 | 27 | 0.0 |
| GP.GA.G | 17.67 | 96 | 0.0 |
| EKSTT | 17.65 | 11 | 0.0 |
| GKL.GPK | 17.62 | 15 | 0.0 |
| TS..IST | 17.62 | 15 | 0.0 |
| T.STTP | 17.60 | 9 | 0.0 |
| GP.G..G | 17.59 | 153 | 0.0 |
| PGPPGPPGP | 17.59 | 59 | 0.0 |
| SE.ST..HS | 17.55 | 2 | 0.0 |
| G.IG..G | 17.54 | 95 | 0.0 |
| GSF.C.C | 17.53 | 27 | 0.0 |
| GK.G.SG | 17.53 | 39 | 0.0 |
| PG.AG..G | 17.53 | 108 | 0.0 |
| G..GE.G | 17.52 | 105 | 0.0 |
| G..C.Q.C | 17.51 | 22 | 0.0 |
| AF..LEQQ | 17.51 | 5 | 0.0 |
| DQ.PS.PR | 17.51 | 5 | 0.0 |
| QEVEEDQD | 17.51 | 5 | 0.0 |
| RSAFY.LE | 17.51 | 5 | 0.0 |
| YRSAF..LE | 17.51 | 5 | 0.0 |
| PSGCLE..D | 17.49 | 5 | 0.0 |
| TPSGCLE | 17.49 | 5 | 0.0 |
| AVD.DEI | 17.46 | 5 | 0.0 |
| DT.T..TT | 17.46 | 2 | 0.0 |
| P..ST…T | 17.45 | 118 | 0.0 |
| TTT..PT | 17.44 | 20 | 0.0 |
| TPT.TPI | 17.44 | 4 | 0.0 |
| GLAVD.D.I | 17.44 | 5 | 0.0 |
| LAVDM.E.E | 17.44 | 5 | 0.0 |
| GPPGPPGPP | 17.43 | 61 | 0.0 |
| C.C.NG | 17.42 | 47 | 0.0 |
| EQQ.VGL.V | 17.42 | 5 | 0.0 |
| G..GP.G | 17.41 | 156 | 0.0 |
| VL..D.C.P | 17.40 | 9 | 0.0 |
| T..EG.TP | 17.38 | 3 | 0.0 |
| P..GY..E | 17.36 | 6 | 0.0 |
| DR.KKD.EE | 17.33 | 5 | 0.0 |
| ED..PPCP | 17.33 | 5 | 0.0 |
| G..SC.CS | 17.33 | 21 | 0.0 |
| TT..TH.P | 17.33 | 7 | 0.0 |
| G..G.PG | 17.33 | 139 | 0.0 |
| LEL.D..QP | 17.32 | 5 | 0.0 |
| G.KGG.G | 17.30 | 42 | 0.0 |
| GCLELTDSC | 17.27 | 5 | 0.0 |
| SGCLE.T.S | 17.27 | 5 | 0.0 |
| ELLEV.EP | 17.22 | 5 | 0.0 |
| T.HSS…T | 17.22 | 4 | 0.0 |
| PPGPPGPPG | 17.21 | 55 | 0.0 |
| P.PPK..E | 17.20 | 20 | 0.0 |
| FTVT.L | 17.15 | 10 | 0.0 |
| HF..S.T.S | 17.15 | 2 | 0.0 |
| VGEIEKK | 17.12 | 5 | 0.0 |
| GAGIP | 17.12 | 16 | 0.0 |
| G.PG..G | 17.11 | 155 | 0.0 |
| DSC.PY.SA | 17.10 | 5 | 0.0 |
| EEDQ.PSC | 17.10 | 5 | 0.0 |
| K.QEVEED | 17.10 | 5 | 0.0 |
| KYQEV.E.Q | 17.10 | 5 | 0.0 |
| QPYRSAF | 17.10 | 5 | 0.0 |
| C…H.G.Y | 17.09 | 13 | 0.0 |
| CLELT.S.Q | 17.07 | 5 | 0.0 |
| PG..G..G | 17.07 | 160 | 0.0 |
| YK.DY | 17.05 | 7 | 0.0 |
| P..IRQ.P | 17.02 | 3 | 0.0 |
| P.STQT | 17.02 | 8 | 0.0 |
| PL..V.V.E | 17.02 | 6 | 0.0 |
| SL.VE..K | 17.02 | 17 | 0.0 |
| VDM.EIEKY | 17.02 | 5 | 0.0 |
| CPPG..G | 17.00 | 54 | 0.0 |
| S.T.SG..E | 16.99 | 19 | 0.0 |
| R.RSRTP | 16.97 | 3 | 0.0 |
| TS.P.ST | 16.95 | 9 | 0.0 |
| EK.YR…E | 16.94 | 9 | 0.0 |
| S.P.ST.SE | 16.94 | 2 | 0.0 |
| GLCGN | 16.93 | 20 | 0.0 |
| G.LGP.G | 16.92 | 77 | 0.0 |
| ALEEKH.G | 16.92 | 5 | 0.0 |
| GPPGP | 16.89 | 103 | 0.0 |
| DQEEEE.Q | 16.89 | 5 | 0.0 |
| TDSCQPY | 16.89 | 5 | 0.0 |
| PVKV.E | 16.86 | 7 | 0.0 |
| V.L.W..P | 16.86 | 7 | 0.0 |
| TTPSP | 16.82 | 16 | 0.0 |
| V.SS.AST | 16.81 | 10 | 0.0 |
| CQ.VCC.P | 16.79 | 16 | 0.0 |
| DG..F.F.G | 16.79 | 13 | 0.0 |
| G..TA.SF | 16.76 | 6 | 0.0 |
| P..NY..E | 16.76 | 6 | 0.0 |
| W.K.G..I | 16.74 | 7 | 0.0 |
| P..STTS | 16.73 | 7 | 0.0 |
| PEVLQDS | 16.73 | 5 | 0.0 |
| DEIEK..EV | 16.71 | 5 | 0.0 |
| DMDEIEKY | 16.71 | 5 | 0.0 |
| M.EIEK..E | 16.71 | 5 | 0.0 |
| PPG.P.V | 16.71 | 52 | 0.0 |
| W.G.DC | 16.69 | 23 | 0.0 |
| Y..EQQ.VG | 16.68 | 5 | 0.0 |
| G…ESTA | 16.64 | 6 | 0.0 |
| GSTHT | 16.64 | 6 | 0.0 |
| P..THTT | 16.64 | 2 | 0.0 |
| TT.TE..S | 16.64 | 8 | 0.0 |
| EVLQDSL | 16.61 | 5 | 0.0 |
| S..S..TT | 16.60 | 119 | 0.0 |
| P.C.QS..C | 16.58 | 38 | 0.0 |
| G.PGA.G | 16.58 | 101 | 0.0 |
| G.CG..NG | 16.58 | 20 | 0.0 |
| ESTA.H | 16.55 | 5 | 0.0 |
| G..GL.C.E | 16.55 | 17 | 0.0 |
| IKAPK | 16.55 | 16 | 0.0 |
| VPD.P..P | 16.55 | 9 | 0.0 |
| T.MP.ST | 16.54 | 2 | 0.0 |
| G..GYPG | 16.53 | 63 | 0.0 |
| VGLAVDM | 16.52 | 5 | 0.0 |
| ITTTT.VT | 16.52 | 4 | 0.0 |
| P..C.F.C | 16.50 | 13 | 0.0 |
| PTSTT | 16.50 | 21 | 0.0 |
| C..G.C.N | 16.50 | 27 | 0.0 |
| TP..IT | 16.48 | 117 | 0.0 |
| T.S.IS.P | 16.47 | 14 | 0.0 |
| G.PGS.G | 16.47 | 96 | 0.0 |
| TL.TTP | 16.45 | 9 | 0.0 |
| E.EEDQGPP | 16.44 | 5 | 0.0 |
| EE.QGPP.P | 16.44 | 5 | 0.0 |
| EEDQGPP | 16.44 | 5 | 0.0 |
| G.AG.PG | 16.42 | 108 | 0.0 |
| G..C.E.C | 16.42 | 23 | 0.0 |
| EIE.Y.EVE | 16.34 | 5 | 0.0 |
| FY..EQQ | 16.34 | 5 | 0.0 |
| I.K.QEVEE | 16.34 | 5 | 0.0 |
| M.ISW | 16.34 | 6 | 0.0 |
| N.AG…C | 16.34 | 28 | 0.0 |
| PP.GPV | 16.34 | 9 | 0.0 |
| PP.PKV | 16.34 | 23 | 0.0 |
| SCQ.YR.A | 16.34 | 5 | 0.0 |
| D..EPEVLQ | 16.30 | 5 | 0.0 |
| LDE.EPEVL | 16.30 | 5 | 0.0 |
| G.TPL…P | 16.29 | 2 | 0.0 |
| P.CCQS..C | 16.29 | 30 | 0.0 |
| TTSAP | 16.29 | 4 | 0.0 |
| STTPV | 16.28 | 4 | 0.0 |
| TT.PG.S | 16.28 | 6 | 0.0 |
| T…TS.PT | 16.23 | 12 | 0.0 |
| P..L.VKE | 16.21 | 13 | 0.0 |
| R.D.K..W | 16.21 | 9 | 0.0 |
| S.SSCC | 16.21 | 35 | 0.0 |
| G..GL.G | 16.21 | 133 | 0.0 |
| GP.G.P | 16.19 | 140 | 0.0 |
| ESTT..S | 16.19 | 3 | 0.0 |
| G.C.NT | 16.18 | 30 | 0.0 |
| ITTTTT.TP | 16.17 | 4 | 0.0 |
| EEK.R.QEE | 16.16 | 5 | 0.0 |
| PGFKG | 16.16 | 53 | 0.0 |
| KKDQE.EE | 16.14 | 5 | 0.0 |
| TF..SP..P | 16.14 | 2 | 0.0 |
| C..NP..C | 16.09 | 20 | 0.0 |
| I…G.KGE | 16.09 | 36 | 0.0 |
| RS.S.QR | 16.06 | 17 | 0.0 |
| V.WFK | 16.06 | 7 | 0.0 |
| TTT.TVTPT | 16.04 | 4 | 0.0 |
| SM.TST | 16.04 | 5 | 0.0 |
| TT.TT..P | 16.03 | 9 | 0.0 |
| G..GH.G | 16.02 | 82 | 0.0 |
| GY.L..DG | 16.02 | 12 | 0.0 |
| G.CG.S.G | 16.01 | 24 | 0.0 |
| W.KD..E | 16.01 | 7 | 0.0 |
| G.SG.PG | 16.01 | 76 | 0.0 |
| G..GE.CQ | 15.99 | 19 | 0.0 |
| C.PYRSA | 15.98 | 5 | 0.0 |
| SG..E.ST | 15.98 | 4 | 0.0 |
| PG.PQ.P | 15.95 | 33 | 0.0 |
| T.SP.TT | 15.92 | 23 | 0.0 |
| T…P..TT | 15.91 | 108 | 0.0 |
| T.D.TT | 15.91 | 105 | 0.0 |
| TT..P..T | 15.89 | 116 | 0.0 |
| C.CVP..S | 15.88 | 21 | 0.0 |
| KW.RV | 15.88 | 30 | 0.0 |
| SIGG..G | 15.88 | 14 | 0.0 |
| TT.GLS | 15.88 | 5 | 0.0 |
| YSTPL | 15.88 | 14 | 0.0 |
| G.P.P.VT | 15.84 | 7 | 0.0 |
| GY.VE | 15.83 | 8 | 0.0 |
| I.V.W.K | 15.82 | 10 | 0.0 |
| G..GQ.G | 15.79 | 103 | 0.0 |
| KG.PTP | 15.77 | 14 | 0.0 |
| P.CC..PV | 15.77 | 33 | 0.0 |
| G..GAPG | 15.74 | 100 | 0.0 |
| P..YY..C | 15.73 | 21 | 0.0 |
| L.V.EP..F | 15.71 | 3 | 0.0 |
| EQQ.VGL | 15.68 | 5 | 0.0 |
| GCGGC | 15.68 | 20 | 0.0 |
| P.TL.FT | 15.63 | 9 | 0.0 |
| T.PGLQ | 15.63 | 25 | 0.0 |
| Y..PGPP | 15.63 | 7 | 0.0 |
| S..STT.H | 15.60 | 3 | 0.0 |
| STTSA | 15.60 | 11 | 0.0 |
| G..GSPG | 15.57 | 91 | 0.0 |
| A.L.V.EP | 15.56 | 9 | 0.0 |
| EGPD.K | 15.56 | 15 | 0.0 |
| V.GL.ED | 15.56 | 6 | 0.0 |
| W.R.D.D | 15.56 | 12 | 0.0 |
| EKI.EQE | 15.54 | 5 | 0.0 |
| G.C.N..G | 15.50 | 41 | 0.0 |
| P…DTT.S | 15.48 | 9 | 0.0 |
| T.STTS | 15.47 | 5 | 0.0 |
| EKI.EQEE | 15.46 | 5 | 0.0 |
| DQGPP.PR | 15.43 | 6 | 0.0 |
| PGV.P.G | 15.43 | 37 | 0.0 |
| STT..RS | 15.43 | 4 | 0.0 |
| TT.SG.S.E | 15.43 | 2 | 0.0 |
| SPP.KP | 15.40 | 12 | 0.0 |
| C..G..CD | 15.38 | 24 | 0.0 |
| G..GRPG | 15.37 | 88 | 0.0 |
| RG..G..G | 15.32 | 128 | 0.0 |
| QEEKIREQE | 15.32 | 5 | 0.0 |
| T.T.QTS | 15.32 | 7 | 0.0 |
| TSA.L..E | 15.32 | 8 | 0.0 |
| PGPPI | 15.29 | 20 | 0.0 |
| T..TST | 15.23 | 114 | 0.0 |
| GP.C…C | 15.20 | 25 | 0.0 |
| ST..V.S.E | 15.20 | 2 | 0.0 |
| G.AGP.G | 15.19 | 93 | 0.0 |
| G..CG.CG | 15.18 | 43 | 0.0 |
| G.F.C.C | 15.18 | 34 | 0.0 |
| QSSC…C | 15.18 | 34 | 0.0 |
| A..SG.MS | 15.13 | 2 | 0.0 |
| A.FEC | 15.13 | 8 | 0.0 |
| G.DG.VG | 15.13 | 33 | 0.0 |
| KM..QEEK | 15.13 | 5 | 0.0 |
| W.R.N.T | 15.13 | 6 | 0.0 |
| G..CA..C | 15.04 | 20 | 0.0 |
| TSIP..T | 15.02 | 6 | 0.0 |
| WTG..C | 15.00 | 32 | 0.0 |
| REQEEK..E | 15.00 | 5 | 0.0 |
| T..TA..ET | 15.00 | 19 | 0.0 |
| K.R.QEEKI | 14.98 | 5 | 0.0 |
| T…P..ST | 14.95 | 112 | 0.0 |
| DK..KAE | 14.95 | 21 | 0.0 |
| PTP..GT | 14.95 | 18 | 0.0 |
| STT.G..E | 14.90 | 4 | 0.0 |
| K.G.S.PS | 14.85 | 6 | 0.0 |
| RIKKDQ | 14.85 | 3 | 0.0 |
| C…PG.C | 14.83 | 18 | 0.0 |
| G.RG..G | 14.81 | 133 | 0.0 |
| A..IS.PS | 14.79 | 14 | 0.0 |
| S..TTST | 14.79 | 12 | 0.0 |
| G..GF.G | 14.76 | 129 | 0.0 |
| C..GF.LS | 14.75 | 16 | 0.0 |
| G..SC.T.C | 14.75 | 13 | 0.0 |
| PGLC..G | 14.75 | 17 | 0.0 |
| STTSG | 14.75 | 4 | 0.0 |
| EERPP | 14.62 | 15 | 0.0 |
| SRPPE | 14.62 | 15 | 0.0 |
| S..TT.T.S | 14.62 | 7 | 0.0 |
| KG.PG | 14.60 | 99 | 0.0 |
| G..CE..C | 14.58 | 30 | 0.0 |
| VSTT…S | 14.58 | 7 | 0.0 |
| G..GD.G | 14.57 | 122 | 0.0 |
| KI.EQEE | 14.56 | 5 | 0.0 |
| G..GK.G | 14.55 | 133 | 0.0 |
| QE.KIR | 14.52 | 11 | 0.0 |
| RQER..K | 14.52 | 2 | 0.0 |
| T…SS.TT | 14.52 | 20 | 0.0 |
| VE.ST..H | 14.52 | 2 | 0.0 |
| T..E..TT | 14.47 | 110 | 0.0 |
| YK..F.K | 14.43 | 8 | 0.0 |
| C…G..WG | 14.43 | 13 | 0.0 |
| KE.E.TA | 14.42 | 9 | 0.0 |
| QEEK..EQ | 14.39 | 5 | 0.0 |
| CQ..CC.P | 14.38 | 52 | 0.0 |
| K.T.T..W | 14.37 | 8 | 0.0 |
| P.PK..W | 14.35 | 21 | 0.0 |
| P.P…T | 14.27 | 295 | 1.0 |
| I.EQEEK | 14.26 | 6 | 0.0 |
| L.S.PV.A | 14.21 | 6 | 0.0 |
| NG.C.N | 14.18 | 15 | 0.0 |
| T.V.SSE | 14.18 | 2 | 0.0 |
| TPITTTTT | 14.18 | 4 | 0.0 |
| G..CS..C | 14.17 | 33 | 0.0 |
| L.VKEP | 14.16 | 6 | 0.0 |
| GPS.P.G | 14.15 | 69 | 0.0 |
| G..GV.G | 14.13 | 118 | 0.0 |
| G..CN..C | 14.08 | 19 | 0.0 |
| STTSH | 14.08 | 3 | 0.0 |
| P…HY..C | 14.07 | 4 | 0.0 |
| WMKG | 14.07 | 10 | 0.0 |
| KG.AG..G | 14.02 | 66 | 0.0 |
| SEPSD | 13.99 | 7 | 0.0 |
| TT.VAS | 13.99 | 3 | 0.0 |
| G..WG..C | 13.95 | 17 | 0.0 |
| F.C.C..G | 13.87 | 38 | 0.0 |
| G..GI.G | 13.82 | 115 | 0.0 |
| PG.PG | 13.74 | 140 | 0.0 |
| C…G..HY | 13.73 | 18 | 0.0 |
| CCVP.C | 13.73 | 35 | 0.0 |
| C.CLPG | 13.71 | 44 | 0.0 |
| T..AS | 13.70 | 212 | 0.0 |
| D..EC…P | 13.67 | 28 | 0.0 |
| C.V.W..P | 13.62 | 15 | 0.0 |
| N…C.PI | 13.62 | 19 | 0.0 |
| TTH.S…T | 13.62 | 2 | 0.0 |
| CC.SS.C | 13.59 | 55 | 0.0 |
| G.EG.PG | 13.57 | 72 | 0.0 |
| C.C.PG..G | 13.56 | 58 | 0.0 |
| SL.W.K | 13.54 | 7 | 0.0 |
| G..GTPG | 13.51 | 84 | 0.0 |
| V.W.K.D | 13.49 | 7 | 0.0 |
| TTST | 13.47 | 118 | 0.0 |
| SA..A.TT | 13.43 | 11 | 0.0 |
| V.W.K…E | 13.43 | 6 | 0.0 |
| G.MG..G | 13.39 | 93 | 0.0 |
| S.PTG..P | 13.38 | 4 | 0.0 |
| D.W.RC | 13.35 | 7 | 0.0 |
| PT..W.K | 13.27 | 6 | 0.0 |
| EQEEKM | 13.23 | 5 | 0.0 |
| G..GM.G | 13.21 | 97 | 0.0 |
| EK.S..W | 13.20 | 6 | 0.0 |
| T…A.STT | 13.20 | 6 | 0.0 |
| SE..YK | 13.19 | 16 | 0.0 |
| I.A.N..G | 13.19 | 6 | 0.0 |
| G.PGI | 13.16 | 112 | 0.0 |
| T.Q.T | 13.10 | 183 | 0.0 |
| P.KPVP | 13.08 | 21 | 0.0 |
| CC.SSC | 13.07 | 45 | 0.0 |
| ST..VT..T | 13.07 | 14 | 0.0 |
| R.S.T…T | 13.07 | 113 | 0.0 |
| C…KG..G | 13.05 | 61 | 0.0 |
| PEAP..V | 13.05 | 3 | 0.0 |
| ER.EQQ | 13.01 | 15 | 0.0 |
| PVSTT | 13.00 | 7 | 0.0 |
| CG.VC..Q | 12.97 | 20 | 0.0 |
| CC.SSC.R | 12.93 | 16 | 0.0 |
| C..D.C.C | 12.92 | 21 | 0.0 |
| CP.G.W | 12.92 | 19 | 0.0 |
| P..TPIT.T | 12.89 | 4 | 0.0 |
| G.VG..G | 12.89 | 126 | 0.0 |
| C.Q.CP | 12.86 | 21 | 0.0 |
| GP.AK.K | 12.81 | 15 | 0.0 |
| S..S.ETT | 12.81 | 3 | 0.0 |
| C.RPSC..S | 12.79 | 16 | 0.0 |
| T.KW.K | 12.76 | 16 | 0.0 |
| I…V.D.P | 12.74 | 13 | 0.0 |
| EEKM..QE | 12.71 | 5 | 0.0 |
| QE.E.KM | 12.71 | 9 | 0.0 |
| QEEKM | 12.71 | 5 | 0.0 |
| R.S.T..W | 12.71 | 6 | 0.0 |
| G..CQ..C | 12.68 | 23 | 0.0 |
| S..STTS | 12.65 | 24 | 0.0 |
| T..E.ST | 12.64 | 112 | 0.0 |
| CC..Q.CQ | 12.59 | 14 | 0.0 |
| P.G.TG | 12.57 | 183 | 0.0 |
| T.TP.TP | 12.57 | 16 | 0.0 |
| PQGP | 12.56 | 128 | 0.0 |
| EYT.V | 12.53 | 8 | 0.0 |
| S.P.A..TT | 12.53 | 9 | 0.0 |
| SPGST | 12.53 | 5 | 0.0 |
| TS..VST | 12.53 | 18 | 0.0 |
| STE.S…T | 12.52 | 17 | 0.0 |
| C…CP.G | 12.52 | 37 | 0.0 |
| D..EYT | 12.48 | 6 | 0.0 |
| KDGKE | 12.48 | 6 | 0.0 |
| D.C.S.P | 12.47 | 31 | 0.0 |
| WEPP | 12.46 | 8 | 0.0 |
| WT.VN | 12.42 | 8 | 0.0 |
| C.CQ.G | 12.39 | 53 | 0.0 |
| CLC..G | 12.38 | 60 | 0.0 |
| K..VH..W | 12.38 | 6 | 0.0 |
| T.TTL.P | 12.38 | 8 | 0.0 |
| CC.PVC | 12.36 | 37 | 0.0 |
| P…GG.K | 12.36 | 28 | 0.0 |
| S.C.Q.TC | 12.32 | 19 | 0.0 |
| TTS.E.S | 12.30 | 12 | 0.0 |
| C.NT.GS | 12.30 | 29 | 0.0 |
| S.PSE.S | 12.30 | 10 | 0.0 |
| CP.G..G | 12.29 | 72 | 0.0 |
| P…FG..C | 12.28 | 26 | 0.0 |
| GCSG..G | 12.25 | 27 | 0.0 |
| E.EKR..QE | 12.23 | 7 | 0.0 |
| HYE.C | 12.23 | 7 | 0.0 |
| C.C.AG..G | 12.20 | 33 | 0.0 |
| A.N.AG | 12.18 | 12 | 0.0 |
| G.PG.G.P | 12.13 | 73 | 0.0 |
| TT..S..GS | 12.13 | 3 | 0.0 |
| C.H.SG.C | 12.10 | 14 | 0.0 |
| EE..QEQE | 12.10 | 5 | 0.0 |
| R.EQQ.R | 12.10 | 2 | 0.0 |
| ST.VH.S | 12.10 | 2 | 0.0 |
| TK..CC | 12.10 | 14 | 0.0 |
| TTA.P.S | 12.10 | 4 | 0.0 |
| GGC.S | 12.09 | 47 | 0.0 |
| C.SSCC | 12.00 | 48 | 0.0 |
| G..GN.G | 11.97 | 110 | 0.0 |
| C.RPSCC | 11.95 | 16 | 0.0 |
| G.NG..G | 11.93 | 91 | 0.0 |
| L.P.STT | 11.90 | 3 | 0.0 |
| C.TTC.RP | 11.89 | 15 | 0.0 |
| G..W..PC | 11.89 | 9 | 0.0 |
| CEP.PC | 11.87 | 13 | 0.0 |
| T…TT.T | 11.84 | 121 | 0.0 |
| GIG.P | 11.84 | 39 | 0.0 |
| C.E.C.C | 11.83 | 27 | 0.0 |
| TPP.PK | 11.80 | 21 | 0.0 |
| TST.L…T | 11.77 | 16 | 0.0 |
| I.G.P.P | 11.76 | 86 | 0.0 |
| G..S.PSE | 11.75 | 8 | 0.0 |
| S…S.TTT | 11.74 | 19 | 0.0 |
| G.D.W.R | 11.67 | 6 | 0.0 |
| SP.TTT | 11.67 | 14 | 0.0 |
| VI..AP.V | 11.67 | 3 | 0.0 |
| YI.EQQ | 11.67 | 3 | 0.0 |
| E…A.F.C | 11.65 | 8 | 0.0 |
| S.T.A.T | 11.65 | 112 | 0.0 |
| SSYGQ | 11.63 | 9 | 0.0 |
| P.CCQ…C | 11.63 | 45 | 0.0 |
| G.P.ET | 11.62 | 109 | 0.0 |
| CGC.H | 11.60 | 4 | 0.0 |
| EK..EQE | 11.60 | 10 | 0.0 |
| C.CH.G | 11.55 | 44 | 0.0 |
| TT..ISS | 11.55 | 6 | 0.0 |
| N..GS..C | 11.52 | 38 | 0.0 |
| PG.SG..C | 11.52 | 39 | 0.0 |
| S..S.YGQ | 11.52 | 9 | 0.0 |
| TS..T.T | 11.50 | 118 | 0.0 |
| D.G.Y..K | 11.45 | 11 | 0.0 |
| T..PD.TT | 11.43 | 4 | 0.0 |
| T…GE..T | 11.43 | 100 | 0.0 |
| V.W.KD | 11.42 | 7 | 0.0 |
| C.CPP | 11.35 | 79 | 0.0 |
| CPP.F | 11.32 | 56 | 0.0 |
| T…VS.T | 11.31 | 108 | 0.0 |
| CCC..G | 11.31 | 39 | 0.0 |
| G.PGTP | 11.31 | 62 | 0.0 |
| D.DEC | 11.30 | 36 | 0.0 |
| C..N.D.C | 11.30 | 26 | 0.0 |
| DV.EC | 11.30 | 37 | 0.0 |
| DG.NC | 11.26 | 25 | 0.0 |
| M.RQEE | 11.24 | 5 | 0.0 |
| S…YR..Y | 11.24 | 10 | 0.0 |
| T.TP.T | 11.24 | 117 | 0.0 |
| P..T.TT | 11.23 | 33 | 0.0 |
| C.C.A…Y | 11.19 | 25 | 0.0 |
| P…RC.C | 11.18 | 42 | 0.0 |
| C.NT.G | 11.18 | 31 | 0.0 |
| GP..DLK | 11.17 | 7 | 0.0 |
| YKAD | 11.17 | 7 | 0.0 |
| C.P.CS | 11.15 | 59 | 0.0 |
| G.HG..G | 11.14 | 92 | 0.0 |
| TTT…S | 11.11 | 127 | 0.0 |
| EEKM..Q | 11.11 | 6 | 0.0 |
| PCHG | 11.09 | 12 | 0.0 |
| QE..R.F.E | 11.09 | 3 | 0.0 |
| S.PSKP | 11.09 | 9 | 0.0 |
| PS..I.S.C | 11.05 | 17 | 0.0 |
| PGPP | 11.04 | 144 | 0.0 |
| CQC..G | 11.04 | 46 | 0.0 |
| GPE.KL..P | 11.02 | 1 | 0.0 |
| T.STE.S | 11.02 | 6 | 0.0 |
| C…DEC | 11.01 | 28 | 0.0 |
| C.S.PC..G | 11.00 | 24 | 0.0 |
| E.MQE.E | 10.99 | 5 | 0.0 |
| TK.T.T | 10.96 | 95 | 0.0 |
| C.Q.C.C | 10.95 | 20 | 0.0 |
| TT..T.GSE | 10.93 | 1 | 0.0 |
| C..GA.C | 10.93 | 39 | 0.0 |
| TC.RP.C | 10.92 | 16 | 0.0 |
| G..GA.G | 10.92 | 137 | 0.0 |
| ITTT | 10.90 | 21 | 0.0 |
| C.C.AG | 10.89 | 56 | 0.0 |
| CCVS..C | 10.84 | 22 | 0.0 |
| C.D..EC | 10.83 | 23 | 0.0 |
| GW.G..C | 10.82 | 35 | 0.0 |
| Q..SR.GS | 10.81 | 21 | 0.0 |
| C.CP.G | 10.80 | 86 | 0.0 |
| C.CA.G | 10.80 | 59 | 0.0 |
| PG..G..C | 10.80 | 56 | 0.0 |
| ETT..ST | 10.79 | 16 | 0.0 |
| TT.HS | 10.79 | 28 | 0.0 |
| V.WY..G | 10.79 | 6 | 0.0 |
| C.C.PG | 10.78 | 78 | 0.0 |
| PGF.G.G | 10.78 | 22 | 0.0 |
| C.PV…C | 10.74 | 46 | 0.0 |
| C..SSC | 10.73 | 69 | 0.0 |
| S..TT..T | 10.73 | 109 | 0.0 |
| PF.PP..P | 10.73 | 26 | 0.0 |
| CEP..C | 10.71 | 31 | 0.0 |
| G.TG..G | 10.70 | 109 | 0.0 |
| W.K.D..L | 10.69 | 7 | 0.0 |
| K..MPG..G | 10.68 | 5 | 0.0 |
| P.GP..F | 10.67 | 90 | 0.0 |
| G..GSGS.Q | 10.66 | 2 | 0.0 |
| K.SA…W | 10.66 | 6 | 0.0 |
| P..CCP | 10.65 | 16 | 0.0 |
| DSSNSSDSS | 10.59 | 1 | 0.0 |
| N..R.D.G | 10.59 | 8 | 0.0 |
| TTP..P | 10.59 | 59 | 0.0 |
| C.N..GS | 10.58 | 33 | 0.0 |
| C.NG.C | 10.57 | 17 | 0.0 |
| G.NC.D | 10.55 | 20 | 0.0 |
| T.T.V…P | 10.55 | 35 | 0.0 |
| PPG.P | 10.54 | 180 | 0.0 |
| EC..NP | 10.54 | 20 | 0.0 |
| C..PC.C | 10.53 | 47 | 0.0 |
| C.L.CP | 10.53 | 21 | 0.0 |
| CC.SVC | 10.52 | 17 | 0.0 |
| E..MW..E | 10.52 | 5 | 0.0 |
| T.TS.TT | 10.49 | 24 | 0.0 |
| RQAPP | 10.47 | 3 | 0.0 |
| PC..G.C | 10.46 | 39 | 0.0 |
| A..ISMP | 10.44 | 1 | 0.0 |
| P..AV..PP | 10.44 | 23 | 0.0 |
| T..N.AG | 10.44 | 28 | 0.0 |
| GSGS.QS | 10.43 | 2 | 0.0 |
| N.PG…C | 10.43 | 29 | 0.0 |
| C…RW.C | 10.42 | 13 | 0.0 |
| C..GW.G | 10.42 | 43 | 0.0 |
| V…QC.C | 10.41 | 19 | 0.0 |
| G.CCP | 10.41 | 20 | 0.0 |
| GS.SGQSS | 10.41 | 2 | 0.0 |
| CH.G.C | 10.40 | 22 | 0.0 |
| D.DV..PK | 10.37 | 3 | 0.0 |
| G.CTC | 10.37 | 16 | 0.0 |
| SGS.QS.G | 10.37 | 2 | 0.0 |
| CTC..G | 10.36 | 60 | 0.0 |
| C…ND.G | 10.36 | 9 | 0.0 |
| TT.S.A.S | 10.34 | 8 | 0.0 |
| CC.P.C | 10.34 | 58 | 0.0 |
| C..SC..C | 10.30 | 23 | 0.0 |
| E.TTS | 10.29 | 109 | 0.0 |
| T…ETTT | 10.29 | 14 | 0.0 |
| T..TT…T | 10.27 | 42 | 0.0 |
| HGS.SG.SS | 10.27 | 2 | 0.0 |
| S..QHGS.S | 10.27 | 2 | 0.0 |
| P.T.P.PP | 10.25 | 35 | 0.0 |
| P..CVSS.C | 10.24 | 22 | 0.0 |
| S..TS..T | 10.23 | 132 | 0.0 |
| C…C..SC | 10.22 | 43 | 0.0 |
| P.F.MPG | 10.21 | 1 | 0.0 |
| R.TG.P.P | 10.21 | 6 | 0.0 |
| T.ST.S | 10.21 | 125 | 0.0 |
| C.NG..C | 10.18 | 50 | 0.0 |
| G.AG..G | 10.18 | 139 | 0.0 |
| CTC.P | 10.18 | 46 | 0.0 |
| T.IT.T | 10.16 | 37 | 0.0 |
| T.G.NC | 10.12 | 26 | 0.0 |
| TT.T..T | 10.12 | 114 | 0.0 |
| C.N.NG | 10.11 | 20 | 0.0 |
| G.SE.SE | 10.09 | 7 | 0.0 |
| GQHG.G | 10.09 | 2 | 0.0 |
| ISMP..D | 10.09 | 1 | 0.0 |
| PF..CH | 10.09 | 19 | 0.0 |
| C.P.CC | 10.08 | 55 | 0.0 |
| SS..GQ.GS | 10.07 | 2 | 0.0 |
| K.KMP.M | 10.05 | 1 | 0.0 |
| PK..MPG | 10.03 | 1 | 0.0 |
| P..G.C.C | 10.02 | 21 | 0.0 |
| T…S..TS | 9.99 | 128 | 0.0 |
| C.DVD | 9.98 | 19 | 0.0 |
| S.CQQ..C | 9.98 | 13 | 0.0 |
| V.WYK | 9.98 | 7 | 0.0 |
| S..GQH.S | 9.97 | 4 | 0.0 |
| VTW.K | 9.95 | 7 | 0.0 |
| CP.G.Y | 9.94 | 33 | 0.0 |
| G.C.C.P | 9.94 | 36 | 0.0 |
| C..G..GP | 9.94 | 61 | 0.0 |
| C…CC.P | 9.92 | 55 | 0.0 |
| S.K.YR | 9.91 | 27 | 0.0 |
| GIPP..PL | 9.90 | 1 | 0.0 |
| PT.TW | 9.90 | 7 | 0.0 |
| SCCQQS | 9.85 | 10 | 0.0 |
| PPG.SG | 9.85 | 62 | 0.0 |
| PD..LK.PK | 9.83 | 1 | 0.0 |
| GY..D..G | 9.82 | 16 | 0.0 |
| HGSGSG.S | 9.80 | 2 | 0.0 |
| P..PP..PK | 9.80 | 21 | 0.0 |
| C.PG..G | 9.78 | 80 | 0.0 |
| P..STT | 9.78 | 51 | 0.0 |
| S..YG.HG | 9.78 | 2 | 0.0 |
| S.PSEP | 9.78 | 6 | 0.0 |
| D.G.Y.F | 9.76 | 9 | 0.0 |
| Q..CQ..CC | 9.74 | 18 | 0.0 |
| KA.N..G | 9.73 | 7 | 0.0 |
| C.P.C…S | 9.70 | 61 | 0.0 |
| C.C..NG | 9.68 | 14 | 0.0 |
| KGPK..MP | 9.66 | 1 | 0.0 |
| HS.P..T | 9.63 | 11 | 0.0 |
| C.CN.G | 9.63 | 32 | 0.0 |
| DV.GP…L | 9.63 | 7 | 0.0 |
| PG.C.N | 9.61 | 17 | 0.0 |
| P.CC…C | 9.61 | 55 | 0.0 |
| T…CCC | 9.60 | 13 | 0.0 |
| PK.SMP | 9.59 | 1 | 0.0 |
| G..CG.C | 9.59 | 46 | 0.0 |
| RCEC | 9.57 | 21 | 0.0 |
| GWG..C | 9.53 | 14 | 0.0 |
| G.GG.PG | 9.53 | 91 | 0.0 |
| CAC..G | 9.51 | 51 | 0.0 |
| MP..K..GP | 9.51 | 1 | 0.0 |
| T..CKV | 9.50 | 12 | 0.0 |
| T…TS..T | 9.49 | 46 | 0.0 |
| G.HGSG.G | 9.48 | 2 | 0.0 |
| GSRH..S | 9.47 | 2 | 0.0 |
| R.QCC | 9.46 | 27 | 0.0 |
| G.HC..R | 9.44 | 13 | 0.0 |
| S.HST.S | 9.44 | 4 | 0.0 |
| G..GS.G | 9.43 | 167 | 0.0 |
| GP.VD.N | 9.43 | 1 | 0.0 |
| G..QC.C | 9.42 | 42 | 0.0 |
| S..GSET | 9.42 | 16 | 0.0 |
| C.N.PG | 9.42 | 27 | 0.0 |
| G.YG..C | 9.41 | 24 | 0.0 |
| GQ..S.SG | 9.40 | 8 | 0.0 |
| C.CL.G | 9.40 | 56 | 0.0 |
| P.P.V.W | 9.40 | 7 | 0.0 |
| C.CD.G | 9.40 | 71 | 0.0 |
| DSSDSSDSS | 9.36 | 1 | 0.0 |
| GQH.S.S | 9.34 | 2 | 0.0 |
| C..S.G.C | 9.34 | 44 | 0.0 |
| PK.KG..D | 9.34 | 1 | 0.0 |
| SDSSDSSDS | 9.34 | 1 | 0.0 |
| CV.D.C | 9.32 | 19 | 0.0 |
| GS.QS…G | 9.32 | 2 | 0.0 |
| GPKV..D | 9.29 | 2 | 0.0 |
| STTS | 9.28 | 46 | 0.0 |
| DG.C…C | 9.28 | 18 | 0.0 |
| C..GG.C | 9.28 | 79 | 0.0 |
| EYTF | 9.27 | 8 | 0.0 |
| CQNG | 9.27 | 41 | 0.0 |
| C.PTC | 9.26 | 47 | 0.0 |
| P..KP..P | 9.26 | 93 | 0.0 |
| C.TTC.R | 9.26 | 19 | 0.0 |
| GSG.S.S.G | 9.25 | 2 | 0.0 |
| TI.W.K | 9.24 | 13 | 0.0 |
| G..GP.C | 9.24 | 49 | 0.0 |
| PC..C.C | 9.24 | 16 | 0.0 |
| PG.CC | 9.24 | 15 | 0.0 |
| G..G..G | 9.23 | 245 | 0.0 |
| PP..P..PK | 9.20 | 22 | 0.0 |
| S.VKY | 9.19 | 15 | 0.0 |
| S.TL.W | 9.17 | 8 | 0.0 |
| STTS..S | 9.17 | 9 | 0.0 |
| G.PG | 9.16 | 294 | 2.0 |
| G.TG..C | 9.15 | 54 | 0.0 |
| TT..TS | 9.14 | 44 | 0.0 |
| T.T.T.P | 9.12 | 114 | 0.0 |
| AAKA | 9.11 | 34 | 0.0 |
| A…Y..AC | 9.08 | 17 | 0.0 |
| CCQQS | 9.08 | 10 | 0.0 |
| D..N.F.C | 9.08 | 25 | 0.0 |
| D.VW.K | 9.08 | 11 | 0.0 |
| G..G.RC | 9.08 | 42 | 0.0 |
| G..H..TF | 9.08 | 28 | 0.0 |
| G.TTA.S | 9.08 | 5 | 0.0 |
| GQ..S.G.H | 9.08 | 2 | 0.0 |
| HGA.C | 9.08 | 24 | 0.0 |
| KGPKFK | 9.08 | 1 | 0.0 |
| LKGP.FK | 9.08 | 1 | 0.0 |
| LKGPKF | 9.08 | 1 | 0.0 |
| P…I.W.K | 9.08 | 6 | 0.0 |
| PK.EG..K | 9.08 | 1 | 0.0 |
| RCSC | 9.08 | 50 | 0.0 |
| S…GRHG | 9.08 | 2 | 0.0 |
| S..HGQ.G | 9.08 | 1 | 0.0 |
| S.SGHSS | 9.08 | 2 | 0.0 |
| S.SGQSS | 9.08 | 2 | 0.0 |
| SSDSSDSSD | 9.08 | 1 | 0.0 |
| TCY…C | 9.08 | 22 | 0.0 |
| VD..LPK | 9.08 | 1 | 0.0 |
| W.KDG | 9.08 | 7 | 0.0 |
| C.PGF | 9.04 | 61 | 0.0 |
| TST | 9.01 | 201 | 0.0 |
| NGG.C | 9.00 | 47 | 0.0 |
| TT.S..T | 8.96 | 123 | 0.0 |
| S.CCQ | 8.96 | 56 | 0.0 |
| LKGP..K | 8.95 | 1 | 0.0 |
| G.C.Y.L | 8.93 | 21 | 0.0 |
| CE..I..C | 8.93 | 19 | 0.0 |
| SG.GQH | 8.89 | 2 | 0.0 |
| CVC..G | 8.87 | 71 | 0.0 |
| E.STT | 8.86 | 28 | 0.0 |
| T.P.T.T | 8.85 | 113 | 0.0 |
| C..S.C.P | 8.85 | 59 | 0.0 |
| C.TTC..T | 8.82 | 19 | 0.0 |
| C.PIC | 8.82 | 28 | 0.0 |
| CT.S.C.Q | 8.81 | 10 | 0.0 |
| CQ.ACC | 8.81 | 14 | 0.0 |
| PCE..P | 8.77 | 34 | 0.0 |
| SG.SSG.G | 8.75 | 17 | 0.0 |
| P..TG..C | 8.75 | 41 | 0.0 |
| C.PSCC | 8.74 | 34 | 0.0 |
| G..EIT | 8.74 | 13 | 0.0 |
| C.HG.C | 8.73 | 41 | 0.0 |
| P.I.W.K | 8.70 | 6 | 0.0 |
| F.G.AC | 8.69 | 41 | 0.0 |
| G..C.D.D | 8.69 | 25 | 0.0 |
| P…G..G | 8.67 | 319 | 1.0 |
| C..GF.G | 8.64 | 59 | 0.0 |
| Q..DS..HS | 8.64 | 1 | 0.0 |
| SD.SDSS | 8.64 | 7 | 0.0 |
| TT..DT | 8.63 | 27 | 0.0 |
| G.EG..G | 8.62 | 90 | 0.0 |
| C.CS.G | 8.62 | 64 | 0.0 |
| G..GR.G | 8.61 | 128 | 0.0 |
| T..RTTC | 8.61 | 17 | 0.0 |
| SGFG.H | 8.60 | 2 | 0.0 |
| TT.AST | 8.60 | 10 | 0.0 |
| C.LPC | 8.58 | 21 | 0.0 |
| KY.I..K | 8.57 | 9 | 0.0 |
| RRQE.E | 8.57 | 2 | 0.0 |
| D.NEC | 8.57 | 28 | 0.0 |
| G..GH.C | 8.56 | 25 | 0.0 |
| GPD..LK | 8.56 | 1 | 0.0 |
| P..TQ.PTT | 8.55 | 4 | 0.0 |
| TP.A.T | 8.54 | 125 | 0.0 |
| C.Q.SC.P | 8.53 | 12 | 0.0 |
| G.FG..C | 8.52 | 36 | 0.0 |
| D..GP.VD | 8.51 | 1 | 0.0 |
| G..CE…N | 8.50 | 17 | 0.0 |
| TK..MT | 8.49 | 11 | 0.0 |
| C.C..G | 8.45 | 154 | 0.0 |
| G.CE…G | 8.45 | 35 | 0.0 |
| C..TC..TT | 8.43 | 19 | 0.0 |
| C.NGG | 8.43 | 55 | 0.0 |
| PV.CK..C | 8.43 | 12 | 0.0 |
| DL.LKG | 8.41 | 8 | 0.0 |
| QK..S…Y | 8.40 | 8 | 0.0 |
| PC..GG.C | 8.38 | 29 | 0.0 |
| C..G.TC | 8.35 | 48 | 0.0 |
| P.VDI..P | 8.34 | 1 | 0.0 |
| TT.CR.T | 8.34 | 19 | 0.0 |
| A.I.G.P | 8.32 | 49 | 0.0 |
| S…RQGS | 8.32 | 8 | 0.0 |
| T.S.TS | 8.31 | 130 | 0.0 |
| DI.EC | 8.31 | 32 | 0.0 |
| PA.RGR | 8.30 | 17 | 0.0 |
| T..WS.P | 8.30 | 8 | 0.0 |
| C..GRC | 8.30 | 34 | 0.0 |
| G.SG..G | 8.29 | 143 | 0.0 |
| C.RP.C | 8.29 | 26 | 0.0 |
| GQ.G..SG | 8.28 | 3 | 0.0 |
| NEC.S | 8.27 | 27 | 0.0 |
| CH..V.P | 8.27 | 20 | 0.0 |
| CRTT…P | 8.25 | 19 | 0.0 |
| LKGP.V | 8.25 | 7 | 0.0 |
| K.D.G.Y | 8.24 | 9 | 0.0 |
| CHC..G | 8.21 | 41 | 0.0 |
| STHT | 8.20 | 16 | 0.0 |
| C.S.PC..A | 8.20 | 11 | 0.0 |
| GP.VD…P | 8.19 | 1 | 0.0 |
| CP.GY | 8.18 | 53 | 0.0 |
| D.GEY | 8.17 | 10 | 0.0 |
| GP.G | 8.16 | 315 | 2.0 |
| S…EC.V | 8.16 | 13 | 0.0 |
| KGPKV | 8.15 | 1 | 0.0 |
| GC.CP | 8.14 | 34 | 0.0 |
| Y.F.G.C | 8.14 | 14 | 0.0 |
| G.CH…G | 8.11 | 24 | 0.0 |
| C..GF.P | 8.11 | 20 | 0.0 |
| CP..SH | 8.11 | 15 | 0.0 |
| RG.CH | 8.10 | 25 | 0.0 |
| CP.GF | 8.09 | 57 | 0.0 |
| T.TT..S | 8.09 | 135 | 0.0 |
| N…C.C.P | 8.05 | 22 | 0.0 |
| G..C…C | 8.03 | 97 | 0.0 |
| TGT.TP.TT | 8.02 | 4 | 0.0 |
| P.VDV..P | 8.02 | 1 | 0.0 |
| T..PTTTPI | 8.01 | 4 | 0.0 |
| TT.E.T | 8.01 | 30 | 0.0 |
| SSDSS.SS | 7.99 | 1 | 0.0 |
| PC.N…C | 7.99 | 29 | 0.0 |
| NG.TC | 7.99 | 46 | 0.0 |
| C.S.PC | 7.98 | 43 | 0.0 |
| SG.SS…Q | 7.98 | 12 | 0.0 |
| G.Y.C.C | 7.97 | 40 | 0.0 |
| TS..TS | 7.93 | 125 | 0.0 |
| TT.K.T | 7.93 | 30 | 0.0 |
| GT.TPTTT | 7.93 | 4 | 0.0 |
| G..TC.C | 7.91 | 39 | 0.0 |
| K.PK..MP | 7.90 | 1 | 0.0 |
| G.TC.P | 7.89 | 30 | 0.0 |
| C..AC..G | 7.87 | 25 | 0.0 |
| GC..D.C | 7.87 | 13 | 0.0 |
| T..L.W.K | 7.84 | 14 | 0.0 |
| GA.HC | 7.82 | 25 | 0.0 |
| PPG.I | 7.81 | 90 | 0.0 |
| GQC..T | 7.81 | 26 | 0.0 |
| DG..C.D | 7.80 | 29 | 0.0 |
| CSC..G | 7.80 | 70 | 0.0 |
| AC.PI | 7.74 | 18 | 0.0 |
| CEI…G | 7.74 | 11 | 0.0 |
| GTC..T | 7.72 | 32 | 0.0 |
| TP.TTPI.T | 7.70 | 4 | 0.0 |
| P..RG.GG | 7.70 | 14 | 0.0 |
| CH.SC | 7.68 | 17 | 0.0 |
| GHSE.S | 7.68 | 4 | 0.0 |
| T.S.TP | 7.67 | 122 | 0.0 |
| C.C.SG | 7.65 | 73 | 0.0 |
| P..CG…C | 7.64 | 24 | 0.0 |
| G..C.CP | 7.64 | 42 | 0.0 |
| N..G..TC | 7.62 | 21 | 0.0 |
| P.PKG…T | 7.62 | 2 | 0.0 |
| CED..E | 7.61 | 32 | 0.0 |
| Y..EYE | 7.60 | 17 | 0.0 |
| T.I.KD | 7.60 | 13 | 0.0 |
| PC..G..C | 7.59 | 38 | 0.0 |
| T.S…T | 7.58 | 188 | 0.0 |
| C..FNG | 7.56 | 22 | 0.0 |
| C..N.TC | 7.56 | 23 | 0.0 |
| C.NGR | 7.56 | 24 | 0.0 |
| G.RCQ | 7.56 | 39 | 0.0 |
| MPEM | 7.56 | 1 | 0.0 |
| T.STVS | 7.56 | 12 | 0.0 |
| C.C.T…G | 7.50 | 26 | 0.0 |
| G..CID | 7.50 | 22 | 0.0 |
| G..GQ.C | 7.49 | 27 | 0.0 |
| C..SCP | 7.48 | 20 | 0.0 |
| G.CSY | 7.48 | 24 | 0.0 |
| S…SG.GH | 7.48 | 2 | 0.0 |
| P..TTS | 7.46 | 113 | 0.0 |
| T.R…T | 7.45 | 198 | 0.0 |
| LKGPK | 7.45 | 2 | 0.0 |
| IP..W…G | 7.44 | 23 | 0.0 |
| NG.SC | 7.44 | 27 | 0.0 |
| GS.TTT | 7.44 | 1 | 0.0 |
| PCH…T | 7.43 | 14 | 0.0 |
| QTP.TTP | 7.43 | 4 | 0.0 |
| PK.KMP | 7.40 | 1 | 0.0 |
| A.V.W.K | 7.38 | 9 | 0.0 |
| G..GD.C | 7.38 | 34 | 0.0 |
| EEE.L..EE | 7.37 | 8 | 0.0 |
| SS.SS.SSD | 7.34 | 1 | 0.0 |
| S.S.TT | 7.33 | 126 | 0.0 |
| S.TTT.S | 7.33 | 10 | 0.0 |
| D.KFR | 7.33 | 12 | 0.0 |
| TW..VS | 7.30 | 7 | 0.0 |
| TTL..T | 7.29 | 131 | 0.0 |
| CE.P.G | 7.26 | 30 | 0.0 |
| DECQ | 7.23 | 20 | 0.0 |
| T…T…T | 7.23 | 189 | 0.0 |
| GPF..C | 7.22 | 9 | 0.0 |
| CV..RC | 7.21 | 29 | 0.0 |
| CT..PC | 7.20 | 37 | 0.0 |
| D.R.G.C | 7.19 | 17 | 0.0 |
| C..FPG | 7.18 | 18 | 0.0 |
| CEC..G | 7.10 | 37 | 0.0 |
| CQQ.C | 7.08 | 29 | 0.0 |
| T.F..TT | 7.06 | 33 | 0.0 |
| T.Y..PG | 7.06 | 35 | 0.0 |
| T…T | 7.05 | 404 | 3.0 |
| F.C.C | 7.04 | 45 | 0.0 |
| EC..SP | 7.04 | 35 | 0.0 |
| Y.CD…D | 7.03 | 12 | 0.0 |
| C.C.EG | 6.99 | 59 | 0.0 |
| V.EP…V | 6.98 | 36 | 0.0 |
| G.RCE | 6.96 | 41 | 0.0 |
| PC.H.G | 6.96 | 27 | 0.0 |
| K.K.H.V | 6.95 | 10 | 0.0 |
| CL..PG | 6.95 | 37 | 0.0 |
| C…C.C | 6.95 | 103 | 0.0 |
| DV..PK.D | 6.94 | 1 | 0.0 |
| CP.GH | 6.93 | 20 | 0.0 |
| P.MT.T | 6.93 | 12 | 0.0 |
| CVS..C | 6.92 | 45 | 0.0 |
| C…P..C | 6.92 | 82 | 0.0 |
| D..K.D.G | 6.90 | 35 | 0.0 |
| EPP.F | 6.89 | 7 | 0.0 |
| P.PP.K | 6.87 | 82 | 0.0 |
| T…TT | 6.86 | 197 | 0.0 |
| TT..S.S | 6.85 | 127 | 0.0 |
| R.N.E.T | 6.85 | 13 | 0.0 |
| GP.CE | 6.83 | 38 | 0.0 |
| S…ST..T | 6.82 | 89 | 0.0 |
| TT…T | 6.81 | 205 | 0.0 |
| C..SC.C | 6.81 | 24 | 0.0 |
| KC.CP | 6.81 | 19 | 0.0 |
| P..CQ.G | 6.81 | 35 | 0.0 |
| C.P.C | 6.80 | 130 | 0.0 |
| S.C.Q.CC | 6.75 | 14 | 0.0 |
| T…T..T | 6.72 | 186 | 0.0 |
| C…PC..P | 6.72 | 21 | 0.0 |
| PP..V.W | 6.70 | 6 | 0.0 |
| G..CE…D | 6.68 | 46 | 0.0 |
| C.C.RG | 6.65 | 34 | 0.0 |
| GS.SC | 6.63 | 45 | 0.0 |
| G.RGSG | 6.60 | 26 | 0.0 |
| PG.C..G | 6.60 | 29 | 0.0 |
| CPP..C | 6.59 | 15 | 0.0 |
| P.GP…C | 6.57 | 43 | 0.0 |
| PTT.H | 6.54 | 21 | 0.0 |
| CRC.P | 6.52 | 25 | 0.0 |
| TC..K…G | 6.51 | 9 | 0.0 |
| CL..PC | 6.50 | 24 | 0.0 |
| QL..QQQ | 6.48 | 27 | 0.0 |
| T..R.RSR | 6.48 | 6 | 0.0 |
| DECS | 6.47 | 25 | 0.0 |
| T..T.TS | 6.47 | 125 | 0.0 |
| G.GCQ | 6.46 | 21 | 0.0 |
| N..G…C | 6.46 | 81 | 0.0 |
| TFDG | 6.44 | 31 | 0.0 |
| CQ..C | 6.44 | 128 | 0.0 |
| C.C.QG | 6.44 | 30 | 0.0 |
| WTK.G | 6.40 | 7 | 0.0 |
| C.C.DG | 6.37 | 47 | 0.0 |
| IC.PG | 6.35 | 42 | 0.0 |
| C…VG.F | 6.34 | 21 | 0.0 |
| C.S.C | 6.28 | 139 | 0.0 |
| REQEE | 6.27 | 8 | 0.0 |
| C.C.N | 6.27 | 68 | 0.0 |
| T.SFT | 6.27 | 15 | 0.0 |
| QHGS | 6.26 | 6 | 0.0 |
| P..ST | 6.23 | 234 | 0.0 |
| TT.S…S | 6.22 | 122 | 0.0 |
| PC.NG | 6.22 | 37 | 0.0 |
| P…EGP | 6.20 | 88 | 0.0 |
| S…S..CQ | 6.19 | 52 | 0.0 |
| P.F.K…P | 6.17 | 10 | 0.0 |
| PP.FV | 6.15 | 22 | 0.0 |
| V..D.PD | 6.14 | 23 | 0.0 |
| C.H.SG | 6.12 | 19 | 0.0 |
| H..GLC | 6.12 | 20 | 0.0 |
| CVD..E | 6.11 | 15 | 0.0 |
| LC.NG | 6.08 | 27 | 0.0 |
| TTTPIT | 6.08 | 4 | 0.0 |
| C.PV.G | 6.05 | 24 | 0.0 |
| T..LT..T | 6.04 | 106 | 0.0 |
| C.P.R.G | 6.00 | 29 | 0.0 |
| W.KAG | 5.96 | 8 | 0.0 |
| T..T.T | 5.95 | 174 | 0.0 |
| G…C.C | 5.95 | 88 | 0.0 |
| TC.DL | 5.95 | 52 | 0.0 |
| C..P..C | 5.94 | 129 | 0.0 |
| P.C.VS | 5.92 | 43 | 0.0 |
| S.PC..G | 5.90 | 34 | 0.0 |
| K..VP.K | 5.88 | 17 | 0.0 |
| T..VT | 5.88 | 195 | 0.0 |
| C…D.C | 5.85 | 85 | 0.0 |
| T…V..TT | 5.84 | 48 | 0.0 |
| T.TT | 5.84 | 200 | 0.0 |
| C.P..C | 5.83 | 123 | 0.0 |
| Q.PTTT.IT | 5.80 | 4 | 0.0 |
| P.G.P | 5.80 | 263 | 1.0 |
| P.GY.C | 5.79 | 25 | 0.0 |
| C…N.AG | 5.78 | 9 | 0.0 |
| P.PC..G | 5.74 | 44 | 0.0 |
| FD.R…F | 5.70 | 15 | 0.0 |
| C.P.PC | 5.69 | 33 | 0.0 |
| PA..TT | 5.68 | 36 | 0.0 |
| PK..MP | 5.68 | 7 | 0.0 |
| CV..PG | 5.66 | 41 | 0.0 |
| TG..CQ | 5.66 | 30 | 0.0 |
| P..PT.T | 5.66 | 49 | 0.0 |
| P.C.R.C | 5.65 | 24 | 0.0 |
| W.CD…D | 5.65 | 3 | 0.0 |
| R.PPG | 5.63 | 125 | 0.0 |
| S.PG.T | 5.62 | 27 | 0.0 |
| VP..K…P | 5.60 | 16 | 0.0 |
| DLC..G | 5.60 | 27 | 0.0 |
| TT.IT | 5.59 | 24 | 0.0 |
| C.S..C | 5.59 | 124 | 0.0 |
| I..AKK | 5.57 | 18 | 0.0 |
| C.Q..C | 5.56 | 108 | 0.0 |
| C…NG.C | 5.54 | 22 | 0.0 |
| IS.P.T | 5.53 | 33 | 0.0 |
| PTT.P.TTT | 5.53 | 4 | 0.0 |
| T.PITT.TT | 5.53 | 4 | 0.0 |
| P…T | 5.52 | 420 | 7.0 |
| S..MST | 5.50 | 20 | 0.0 |
| G..SG.S.S | 5.50 | 7 | 0.0 |
| GR.C.D | 5.49 | 15 | 0.0 |
| W.K.E.V | 5.49 | 11 | 0.0 |
| DV.GP | 5.49 | 16 | 0.0 |
| T.R.D.K | 5.48 | 14 | 0.0 |
| C…SC | 5.47 | 138 | 0.0 |
| TT..F.T | 5.47 | 23 | 0.0 |
| Y.C.C | 5.45 | 62 | 0.0 |
| C..G..G | 5.45 | 182 | 0.0 |
| G..G..C | 5.42 | 141 | 0.0 |
| VVE.R | 5.40 | 14 | 0.0 |
| P…L.VT | 5.37 | 22 | 0.0 |
| RC.C | 5.36 | 96 | 0.0 |
| V.DVT | 5.36 | 28 | 0.0 |
| TT..T | 5.35 | 168 | 0.0 |
| T..K.TT | 5.35 | 33 | 0.0 |
| APKI | 5.34 | 11 | 0.0 |
| T..P | 5.34 | 405 | 6.0 |
| C..SC | 5.33 | 128 | 0.0 |
| C.CE.G | 5.32 | 35 | 0.0 |
| PCP.G | 5.30 | 59 | 0.0 |
| GPDC | 5.29 | 32 | 0.0 |
| HGQ.G | 5.26 | 7 | 0.0 |
| P..T.T | 5.25 | 199 | 0.0 |
| IT.N..T | 5.23 | 13 | 0.0 |
| T.PIT | 5.19 | 28 | 0.0 |
| T…S.T | 5.14 | 216 | 0.0 |
| D.N..GP | 5.14 | 6 | 0.0 |
| TT.T | 5.13 | 191 | 0.0 |
| VK..EP | 5.13 | 11 | 0.0 |
| C…GTP | 5.13 | 6 | 0.0 |
| C..IPG | 5.12 | 11 | 0.0 |
| G..TC…N | 5.11 | 7 | 0.0 |
| T.T.T | 5.10 | 179 | 0.0 |
| A.V.EP | 5.10 | 14 | 0.0 |
| CQ…C | 5.08 | 141 | 0.0 |
| PGP | 5.07 | 230 | 0.0 |
| K..MPK..M | 5.07 | 1 | 0.0 |
| VT | 5.05 | 398 | 4.0 |
| ST | 5.02 | 377 | 6.0 |
| V..IK.P | 5.01 | 10 | 0.0 |
| D…R..PP | 5.00 | 55 | 0.0 |
| I..PS.P | 5.00 | 40 | 0.0 |
| G.P.P | 4.99 | 309 | 1.0 |
| T..TV.D | 4.98 | 11 | 0.0 |
| K..I..DG | 4.97 | 7 | 0.0 |
| IPI.G | 4.95 | 19 | 0.0 |
| W.K.N | 4.92 | 10 | 0.0 |
| V.W.K | 4.91 | 15 | 0.0 |
| C..TLG | 4.91 | 26 | 0.0 |
| D…PP.I | 4.90 | 8 | 0.0 |
| P.D.P.F | 4.90 | 20 | 0.0 |
| CE..C | 4.90 | 108 | 0.0 |
| DG.EI | 4.89 | 8 | 0.0 |
| VIE.R | 4.89 | 12 | 0.0 |
| T..S | 4.87 | 404 | 6.0 |
| C..GF.L | 4.87 | 33 | 0.0 |
| C.P.SC | 4.85 | 36 | 0.0 |
| C…C..G | 4.85 | 113 | 0.0 |
| CP..CP | 4.84 | 21 | 0.0 |
| P…AP..C | 4.84 | 29 | 0.0 |
| T.KAG | 4.84 | 24 | 0.0 |
| TT | 4.83 | 382 | 3.0 |
| S..GQ..S | 4.82 | 26 | 0.0 |
| S.G.HG | 4.81 | 8 | 0.0 |
| A.NS.G | 4.80 | 29 | 0.0 |
| QQ..LQ.Q | 4.78 | 13 | 0.0 |
| PP.F…L | 4.77 | 15 | 0.0 |
| KGE | 4.77 | 161 | 0.0 |
| C.N..C | 4.76 | 73 | 0.0 |
| TCQ.S | 4.75 | 17 | 0.0 |
| C…N.C | 4.73 | 59 | 0.0 |
| MSTP | 4.72 | 11 | 0.0 |
| GP.VD | 4.72 | 7 | 0.0 |
| C..D…C | 4.70 | 73 | 0.0 |
| PG.CG | 4.69 | 39 | 0.0 |
| TP | 4.68 | 440 | 5.5 |
| V.SS…T | 4.68 | 28 | 0.0 |
| C…G.C | 4.68 | 121 | 0.0 |
| G.C…C | 4.67 | 97 | 0.0 |
| TP.T..S | 4.67 | 53 | 0.0 |
| T..GT.P | 4.67 | 50 | 0.0 |
| S.P.T…P | 4.65 | 80 | 0.0 |
| P..PP..P | 4.61 | 187 | 0.0 |
| IT..S.T | 4.61 | 23 | 0.0 |
| VDTT | 4.59 | 17 | 0.0 |
| T..TT.S | 4.58 | 50 | 0.0 |
| C…PC | 4.58 | 84 | 0.0 |
| TD..T | 4.58 | 175 | 0.0 |
| KVT…K | 4.57 | 15 | 0.0 |
| TS | 4.56 | 403 | 5.0 |
| T..TS | 4.55 | 201 | 0.0 |
| S…S…T | 4.55 | 240 | 0.0 |
| CP.G | 4.55 | 133 | 0.0 |
| C.CR.G | 4.54 | 47 | 0.0 |
| CP.GL | 4.54 | 49 | 0.0 |
| C.Q.C | 4.52 | 59 | 0.0 |
| D…DC.D | 4.51 | 8 | 0.0 |
| TT…P | 4.50 | 225 | 0.0 |
| C..TS..V | 4.48 | 13 | 0.0 |
| T..A | 4.46 | 410 | 4.0 |
| TPA.C | 4.46 | 15 | 0.0 |
| G.CVP | 4.45 | 46 | 0.0 |
| TS..T | 4.45 | 196 | 0.0 |
| C..N…C | 4.44 | 55 | 0.0 |
| GH.E.S | 4.43 | 14 | 0.0 |
| VERR | 4.43 | 20 | 0.0 |
| RVN..L | 4.40 | 37 | 0.0 |
| G.C.C | 4.40 | 102 | 0.0 |
| T…STC | 4.39 | 30 | 0.0 |
| WFK | 4.39 | 16 | 0.0 |
| TPSP | 4.38 | 52 | 0.0 |
| T…SS..T | 4.35 | 49 | 0.0 |
| C…T..C | 4.33 | 73 | 0.0 |
| T…I..T | 4.32 | 152 | 0.0 |
| C.P…C | 4.29 | 102 | 0.0 |
| K..PFP | 4.29 | 12 | 0.0 |
| VDV..P | 4.29 | 20 | 0.0 |
| T.S.H…S | 4.27 | 30 | 0.0 |
| T.S.T | 4.27 | 216 | 0.0 |
| C..TC | 4.26 | 92 | 0.0 |
| TP.F..A | 4.25 | 16 | 0.0 |
| TT.S | 4.23 | 191 | 0.0 |
| C…TC | 4.22 | 97 | 0.0 |
| PV | 4.22 | 461 | 7.0 |
| C…LC.P | 4.21 | 26 | 0.0 |
| GP.C | 4.18 | 152 | 0.0 |
| T.A…T | 4.18 | 201 | 0.0 |
| S.TT | 4.17 | 206 | 0.0 |
| T…G..T | 4.17 | 195 | 0.0 |
| T.T | 4.16 | 402 | 4.0 |
| GRCI | 4.15 | 21 | 0.0 |
| C…RC | 4.13 | 78 | 0.0 |
| C..GC | 4.12 | 98 | 0.0 |
| P…C.C | 4.11 | 55 | 0.0 |
| S..S.TH | 4.11 | 24 | 0.0 |
| G.P.I | 4.10 | 193 | 0.0 |
| T.K..TT | 4.10 | 27 | 0.0 |
| EC.C | 4.08 | 80 | 0.0 |
| Q..DS..H | 4.08 | 1 | 0.0 |
| TC.C | 4.07 | 90 | 0.0 |
| T.TA…T | 4.05 | 40 | 0.0 |
| C.PG | 4.05 | 149 | 0.0 |
| V.IK.P | 4.03 | 13 | 0.0 |
| PP.A..K | 4.02 | 65 | 0.0 |
| KTS.R | 4.01 | 20 | 0.0 |
| C..GY | 4.00 | 89 | 0.0 |
| W.K…E | 3.97 | 29 | 0.0 |
| SC.C | 3.96 | 145 | 0.0 |
| G..CE | 3.96 | 113 | 0.0 |
| C…A.C | 3.94 | 96 | 0.0 |
| TT..G | 3.92 | 177 | 0.0 |
| V.TAT | 3.90 | 42 | 0.0 |
| PDV…G | 3.88 | 8 | 0.0 |
| E.TT | 3.88 | 174 | 0.0 |
| T.H…T | 3.87 | 146 | 0.0 |
| E.T.S.T | 3.86 | 27 | 0.0 |
| W.K.G | 3.85 | 14 | 0.0 |
| D.G.Y | 3.84 | 39 | 0.0 |
| P.S..PV | 3.83 | 80 | 0.0 |
| PV.PP | 3.81 | 89 | 0.0 |
| P..DLK | 3.81 | 10 | 0.0 |
| R..ER.RER | 3.80 | 2 | 0.0 |
| CR..C | 3.76 | 67 | 0.0 |
| TP.T | 3.75 | 211 | 0.0 |
| T…S.P | 3.74 | 252 | 1.0 |
| TT.H | 3.74 | 70 | 0.0 |
| T..M.T | 3.73 | 128 | 0.0 |
| CH..C | 3.73 | 57 | 0.0 |
| P..C..G | 3.69 | 125 | 0.0 |
| G..C.D | 3.69 | 87 | 0.0 |
| G..CQ | 3.67 | 104 | 0.0 |
| TP.V…P | 3.64 | 35 | 0.0 |
| T.P..T | 3.64 | 186 | 0.0 |
| W..DG | 3.64 | 20 | 0.0 |
| P..P..P | 3.61 | 257 | 1.0 |
| P..D | 3.59 | 392 | 6.0 |
| C..Q.C | 3.59 | 87 | 0.0 |
| PGF | 3.58 | 203 | 0.0 |
| S.S | 3.55 | 455 | 9.0 |
| K.KTT | 3.55 | 31 | 0.0 |
| PC.N | 3.54 | 61 | 0.0 |
| V..PKV | 3.54 | 10 | 0.0 |
| TAK.K | 3.54 | 33 | 0.0 |
| IS.PS | 3.51 | 34 | 0.0 |
| NG.C | 3.50 | 65 | 0.0 |
| TLT..P | 3.50 | 41 | 0.0 |
| Q.TC | 3.49 | 87 | 0.0 |
| N.AG | 3.48 | 88 | 0.0 |
| W..GG..G | 3.46 | 19 | 0.0 |
| PP.LP | 3.42 | 143 | 0.0 |
| T…E.T | 3.42 | 184 | 0.0 |
| T..L..TT | 3.41 | 27 | 0.0 |
| V.D.P | 3.40 | 151 | 0.0 |
| C..G…C | 3.40 | 100 | 0.0 |
| G…C.P | 3.38 | 137 | 0.0 |
| G.TC | 3.38 | 145 | 0.0 |
| C…C..T | 3.37 | 72 | 0.0 |
| C.S…G | 3.37 | 129 | 0.0 |
| R.G.S.R | 3.34 | 28 | 0.0 |
| C..N.C | 3.33 | 72 | 0.0 |
| TPS | 3.30 | 239 | 0.0 |
| TAT | 3.29 | 197 | 0.0 |
| PSPC | 3.27 | 34 | 0.0 |
| CE…C | 3.27 | 87 | 0.0 |
| CS…C | 3.26 | 136 | 0.0 |
| P.P | 3.26 | 456 | 24.0 |
| RTT | 3.24 | 182 | 0.0 |
| TI…T | 3.23 | 147 | 0.0 |
| QQQQQQQQQ | 3.23 | 11 | 0.0 |
| TL..T | 3.21 | 215 | 0.0 |
| C..H..C | 3.21 | 59 | 0.0 |
| C…QC | 3.20 | 80 | 0.0 |
| CA…C | 3.19 | 86 | 0.0 |
| DV..P..E | 3.18 | 13 | 0.0 |
| TTA | 3.18 | 178 | 0.0 |
| TG..C | 3.15 | 89 | 0.0 |
| S..S.T | 3.15 | 210 | 0.0 |
| PCH | 3.15 | 54 | 0.0 |
| WE.P | 3.14 | 27 | 0.0 |
| KYK | 3.14 | 37 | 0.0 |
| P..P.PPP | 3.13 | 74 | 0.0 |
| C..P…T | 3.13 | 133 | 0.0 |
| S.PLT | 3.13 | 19 | 0.0 |
| KG.C | 3.13 | 122 | 0.0 |
| C…C | 3.12 | 223 | 0.0 |
| C.PV | 3.12 | 107 | 0.0 |
| G.NC | 3.12 | 68 | 0.0 |
| S…RHG | 3.11 | 11 | 0.0 |
| T.S | 3.11 | 386 | 6.0 |
| G.GG..G | 3.08 | 141 | 0.0 |
| PP..R…T | 3.08 | 51 | 0.0 |
| S..M.T | 3.06 | 150 | 0.0 |
| C.CT | 3.05 | 59 | 0.0 |
| VPG | 3.05 | 289 | 1.0 |
| L.V.D.T | 3.04 | 18 | 0.0 |
| C..N..C | 3.04 | 71 | 0.0 |
| GQ..S | 3.04 | 270 | 1.0 |
| QQ.QQQQ | 3.04 | 13 | 0.0 |
| P..F…C | 3.03 | 71 | 0.0 |
| TC..G | 3.02 | 101 | 0.0 |
| HG.C | 3.01 | 71 | 0.0 |
| TK…T | 3.01 | 138 | 0.0 |
| PCL..G | 3.00 | 16 | 0.0 |
| T.R.S | 2.99 | 183 | 0.0 |
| G…GG.C | 2.97 | 23 | 0.0 |
| AT | 2.97 | 397 | 4.0 |
| P..G…C | 2.96 | 131 | 0.0 |
| C.C | 2.96 | 246 | 0.0 |
| D..APD | 2.96 | 17 | 0.0 |
| G.RC | 2.93 | 103 | 0.0 |
| D.T | 2.92 | 345 | 3.0 |
| P.K…P | 2.91 | 212 | 0.0 |
| PP..P | 2.90 | 329 | 1.0 |
| V..T | 2.90 | 361 | 3.0 |
| Q..QA…Q | 2.89 | 33 | 0.0 |
| C.H.C | 2.88 | 37 | 0.0 |
| T..F..S | 2.88 | 145 | 0.0 |
| TS.T | 2.87 | 180 | 0.0 |
| C..D..C | 2.86 | 92 | 0.0 |
| TCQ | 2.86 | 119 | 0.0 |
| TC.D | 2.85 | 101 | 0.0 |
| G…SG.G | 2.85 | 64 | 0.0 |
| M..PG | 2.85 | 186 | 0.0 |
| GG.G.P | 2.84 | 108 | 0.0 |
| P.PPS | 2.84 | 175 | 0.0 |
| G.CH | 2.84 | 81 | 0.0 |
| TTF | 2.83 | 127 | 0.0 |
| EQ..PP | 2.82 | 26 | 0.0 |
| S.PT | 2.80 | 230 | 0.0 |
| C.EG | 2.79 | 102 | 0.0 |
| G.C..C | 2.79 | 64 | 0.0 |
| G.CQ | 2.78 | 98 | 0.0 |
| LP | 2.78 | 429 | 14.0 |
| S.A..T | 2.77 | 226 | 0.0 |
| G.C.P | 2.77 | 128 | 0.0 |
| Q…R.E.E | 2.77 | 23 | 0.0 |
| G..G..PP | 2.77 | 74 | 0.0 |
| K.T.T | 2.76 | 167 | 0.0 |
| R.T.T | 2.76 | 188 | 0.0 |
| T..M..T | 2.74 | 126 | 0.0 |
| V…K..DV | 2.71 | 16 | 0.0 |
| T..W.R | 2.70 | 29 | 0.0 |
| PG.C | 2.70 | 126 | 0.0 |
| PP.K | 2.68 | 169 | 0.0 |
| Q…Q.QQ | 2.67 | 37 | 0.0 |
| TK.L…E | 2.66 | 18 | 0.0 |
| T.G…T | 2.66 | 201 | 0.0 |
| P..T.P | 2.65 | 306 | 1.0 |
| P.AP..P | 2.63 | 118 | 0.0 |
| TS..L..T | 2.63 | 44 | 0.0 |
| PC…C | 2.62 | 54 | 0.0 |
| G.CV | 2.62 | 113 | 0.0 |
| PG | 2.62 | 445 | 11.0 |
| V..E.S.S | 2.60 | 50 | 0.0 |
| T..V…T | 2.57 | 165 | 0.0 |
| G.DC | 2.55 | 83 | 0.0 |
| S..I.T | 2.54 | 167 | 0.0 |
| CE.P | 2.54 | 138 | 0.0 |
| G…S.C | 2.52 | 114 | 0.0 |
| PSP.R | 2.51 | 67 | 0.0 |
| KD.K…S | 2.51 | 32 | 0.0 |
| P..E…P | 2.50 | 244 | 0.0 |
| G…E.C | 2.50 | 100 | 0.0 |
| GSC | 2.49 | 150 | 0.0 |
| T..P..P | 2.49 | 283 | 1.0 |
| TTC | 2.48 | 78 | 0.0 |
| TC..V | 2.47 | 82 | 0.0 |
| T..IS | 2.46 | 191 | 0.0 |
| CP..C | 2.46 | 62 | 0.0 |
| DG.C | 2.44 | 87 | 0.0 |
| CD…G | 2.42 | 79 | 0.0 |
| S..S.SS | 2.40 | 182 | 0.0 |
| DG..C | 2.39 | 78 | 0.0 |
| VT..T | 2.38 | 160 | 0.0 |
| C..R.C | 2.38 | 68 | 0.0 |
| C.DG | 2.38 | 83 | 0.0 |
| GP | 2.37 | 433 | 8.0 |
| C..S…G | 2.37 | 148 | 0.0 |
| LEEK | 2.36 | 46 | 0.0 |
| Y..EM | 2.35 | 30 | 0.0 |
| Y.L..D | 2.34 | 54 | 0.0 |
| A…P | 2.34 | 437 | 10.0 |
| R.SC | 2.34 | 128 | 0.0 |
| PTP | 2.34 | 241 | 0.0 |
| T.T.D | 2.33 | 181 | 0.0 |
| G.G.G..G | 2.33 | 148 | 0.0 |
| P.FK | 2.33 | 158 | 0.0 |
| PPP…P | 2.32 | 105 | 0.0 |
| P.APP | 2.32 | 63 | 0.0 |
| T…EC | 2.30 | 82 | 0.0 |
| T.PI | 2.30 | 90 | 0.0 |
| TLP.S | 2.29 | 59 | 0.0 |
| SG..C | 2.28 | 119 | 0.0 |
| V.EP | 2.28 | 119 | 0.0 |
| G.QC | 2.27 | 82 | 0.0 |
| TN…T | 2.27 | 136 | 0.0 |
| D.G…C | 2.26 | 63 | 0.0 |
| V.AK | 2.25 | 81 | 0.0 |
| PP…T | 2.25 | 211 | 0.0 |
| SC..G | 2.24 | 109 | 0.0 |
| VS.T | 2.24 | 169 | 0.0 |
| G.HC | 2.23 | 69 | 0.0 |
| GQ..C | 2.22 | 94 | 0.0 |
| E..PP | 2.22 | 299 | 1.0 |
| P…M..P | 2.20 | 66 | 0.0 |
| P..S.E | 2.20 | 254 | 1.0 |
| P…HC | 2.20 | 69 | 0.0 |
| C..H.C | 2.19 | 34 | 0.0 |
| G..G…G | 2.19 | 263 | 1.0 |
| T.F.T | 2.18 | 139 | 0.0 |
| YT..C | 2.18 | 41 | 0.0 |
| T.L…T | 2.18 | 204 | 0.0 |
| N.T…C | 2.16 | 49 | 0.0 |
| QERE | 2.16 | 30 | 0.0 |
| CV.T | 2.15 | 73 | 0.0 |
| KTT | 2.13 | 160 | 0.0 |
| S.T.P | 2.13 | 223 | 0.0 |
| E…D.C | 2.12 | 79 | 0.0 |
| N.TC | 2.11 | 77 | 0.0 |
| P…P | 2.11 | 441 | 18.5 |
| C.A..C | 2.11 | 75 | 0.0 |
| V..P | 2.09 | 422 | 8.0 |
| G…G..PA | 2.08 | 51 | 0.0 |
| V..CQ | 2.08 | 65 | 0.0 |
| P.LP | 2.08 | 319 | 1.0 |
| TCK | 2.06 | 70 | 0.0 |
| Q.QQQ | 2.06 | 37 | 0.0 |
| RPG | 2.03 | 226 | 0.0 |
| GHC | 2.02 | 93 | 0.0 |
| P…G..P | 2.02 | 196 | 0.0 |
| P…VP | 2.01 | 242 | 0.0 |
| C.P | 2.01 | 313 | 1.0 |
| C..LS | 2.00 | 93 | 0.0 |
| RCH | 2.00 | 77 | 0.0 |
| GA.G.G | 2.00 | 116 | 0.0 |
| EP.E | 2.00 | 218 | 0.0 |
| VK..V..S | 2.00 | 10 | 0.0 |
| C..V…T | 2.00 | 92 | 0.0 |
| G..C | 2.00 | 327 | 1.0 |
| CEI | 1.99 | 41 | 0.0 |
| G.SC | 1.98 | 114 | 0.0 |
| P.PC | 1.97 | 121 | 0.0 |
| Q.T | 1.97 | 388 | 3.0 |
| P…QC | 1.96 | 95 | 0.0 |
| P..I…P | 1.95 | 142 | 0.0 |
| C.A.N | 1.94 | 45 | 0.0 |
| T..L…P | 1.92 | 231 | 0.0 |
| TTL | 1.92 | 173 | 0.0 |
| T..V | 1.92 | 385 | 3.0 |
| P..P | 1.91 | 430 | 11.0 |
| CR.T | 1.91 | 66 | 0.0 |
| TCT | 1.90 | 77 | 0.0 |
| TIT | 1.90 | 155 | 0.0 |
| YG..G | 1.90 | 153 | 0.0 |
| Y…Y.E | 1.88 | 79 | 0.0 |
| TVS | 1.87 | 189 | 0.0 |
| V…E..T | 1.87 | 109 | 0.0 |
| R.TC | 1.86 | 89 | 0.0 |
| P.PPP | 1.86 | 108 | 0.0 |
| T.PG | 1.86 | 270 | 1.0 |
| T.EC | 1.85 | 63 | 0.0 |
| R.C.C | 1.85 | 59 | 0.0 |
| P.G.Y | 1.83 | 114 | 0.0 |
| P…PP | 1.83 | 277 | 1.0 |
| E..KG | 1.82 | 194 | 0.0 |
| GLGE | 1.82 | 36 | 0.0 |
| NV…D | 1.82 | 62 | 0.0 |
| V..G | 1.81 | 404 | 4.0 |
| DG..R | 1.80 | 184 | 0.0 |
| P..A..T | 1.80 | 216 | 0.0 |
| P.D…P | 1.80 | 145 | 0.0 |
| GG..G | 1.79 | 327 | 1.0 |
| P.RF | 1.79 | 100 | 0.0 |
| CQ | 1.78 | 297 | 1.0 |
| G.C | 1.78 | 318 | 1.0 |
| K.TT | 1.76 | 86 | 0.0 |
| Q..P | 1.76 | 412 | 9.0 |
| NCT | 1.76 | 62 | 0.0 |
| T…AT | 1.75 | 169 | 0.0 |
| PP | 1.75 | 429 | 11.0 |
| F.S.P | 1.74 | 188 | 0.0 |
| P..L..P | 1.73 | 253 | 1.0 |
| D.PD | 1.72 | 85 | 0.0 |
| TTK | 1.72 | 86 | 0.0 |
| EYR | 1.70 | 45 | 0.0 |
| EP..I | 1.69 | 88 | 0.0 |
| P.S | 1.69 | 438 | 11.0 |
| G…G…G | 1.68 | 248 | 0.0 |
| D..D…C | 1.67 | 33 | 0.0 |
| S.P | 1.65 | 452 | 9.0 |
| AT.T | 1.64 | 182 | 0.0 |
| D.C | 1.64 | 256 | 1.0 |
| P.S..P | 1.64 | 294 | 1.0 |
| P.R…P | 1.63 | 180 | 0.0 |
| N.FG | 1.63 | 42 | 0.0 |
| CQA | 1.62 | 74 | 0.0 |
| K.VT | 1.62 | 182 | 0.0 |
| R..PP | 1.61 | 169 | 0.0 |
| G…C | 1.59 | 335 | 1.0 |
| E.S..T | 1.57 | 198 | 0.0 |
| DPP | 1.57 | 81 | 0.0 |
| G.PV | 1.56 | 201 | 0.0 |
| I..TR | 1.56 | 91 | 0.0 |
| S..I.S | 1.55 | 196 | 0.0 |
| P.I..G | 1.54 | 119 | 0.0 |
| C…S.P | 1.54 | 108 | 0.0 |
| TS..L | 1.54 | 222 | 0.0 |
| C..T | 1.54 | 312 | 1.0 |
| WR.P | 1.53 | 37 | 0.0 |
| E.PK | 1.53 | 125 | 0.0 |
| K…A.P | 1.52 | 107 | 0.0 |
| P…A..P | 1.51 | 202 | 0.0 |
| SG..S | 1.51 | 261 | 1.0 |
| GVC | 1.51 | 95 | 0.0 |
| C..GS | 1.49 | 95 | 0.0 |
| T…K..V | 1.49 | 70 | 0.0 |
| SC | 1.49 | 346 | 1.0 |
| T…G | 1.48 | 422 | 9.0 |
| T…L..T | 1.48 | 189 | 0.0 |
| VP.P | 1.47 | 182 | 0.0 |
| K…E..K | 1.47 | 90 | 0.0 |
| Q.Q…Q | 1.46 | 140 | 0.0 |
| CK..T | 1.46 | 48 | 0.0 |
| PA…P | 1.45 | 282 | 1.0 |
| G..L…G | 1.45 | 262 | 1.0 |
| S..V | 1.44 | 428 | 4.0 |
| G.S..C | 1.44 | 116 | 0.0 |
| V…D | 1.44 | 343 | 2.0 |
| G…T | 1.44 | 421 | 8.0 |
| R.G…S | 1.43 | 222 | 0.0 |
| T..I..S | 1.42 | 123 | 0.0 |
| G.G.G | 1.42 | 274 | 1.0 |
| P..Q…P | 1.41 | 175 | 0.0 |
| P…L.P | 1.41 | 208 | 0.0 |
| PL…P | 1.41 | 212 | 0.0 |
| IE..E | 1.39 | 55 | 0.0 |
| P…EP | 1.38 | 146 | 0.0 |
| C.H…G | 1.38 | 53 | 0.0 |
| E…V…E | 1.38 | 89 | 0.0 |
| PP..E | 1.37 | 172 | 0.0 |
| TA | 1.36 | 404 | 5.0 |
| PSP | 1.35 | 179 | 0.0 |
| KE.T | 1.34 | 105 | 0.0 |
| CH | 1.34 | 224 | 0.0 |
| S..F.C | 1.34 | 41 | 0.0 |
| P…Q…A | 1.34 | 131 | 0.0 |
| K…V…K | 1.33 | 55 | 0.0 |
| P…FP | 1.33 | 156 | 0.0 |
| MP | 1.33 | 329 | 2.0 |
| RC | 1.33 | 244 | 0.0 |
| E.T | 1.32 | 367 | 3.0 |
| I.E.E | 1.32 | 82 | 0.0 |
| S.SA | 1.32 | 283 | 1.0 |
| CE | 1.31 | 293 | 1.0 |
| EVS | 1.31 | 133 | 0.0 |
| S…M..Q | 1.30 | 54 | 0.0 |
| V.KD | 1.30 | 78 | 0.0 |
| K.DA | 1.30 | 96 | 0.0 |
| QQ..Q | 1.30 | 98 | 0.0 |
| R.T.I | 1.30 | 54 | 0.0 |
| E..T | 1.29 | 385 | 4.0 |
| T..LE | 1.29 | 195 | 0.0 |
| GT | 1.28 | 413 | 9.0 |
| C..N | 1.28 | 192 | 0.0 |
| G.FC | 1.28 | 52 | 0.0 |
| Y…C | 1.28 | 216 | 0.0 |
| E.S.S | 1.28 | 261 | 1.0 |
| G..IC | 1.27 | 47 | 0.0 |
| M.EC | 1.27 | 31 | 0.0 |
| LK.T | 1.27 | 129 | 0.0 |
| G.S | 1.27 | 475 | 12.0 |
| T.V.E | 1.26 | 145 | 0.0 |
| P…L | 1.26 | 442 | 7.0 |
| QA | 1.25 | 421 | 4.0 |
| TI | 1.25 | 299 | 1.0 |
| RPP | 1.25 | 138 | 0.0 |
| E..K.K | 1.25 | 89 | 0.0 |
| P.V | 1.25 | 415 | 6.0 |
| P…H.P | 1.25 | 112 | 0.0 |
| GC | 1.25 | 314 | 1.0 |
| D.R…T | 1.25 | 63 | 0.0 |
| DF.T | 1.24 | 105 | 0.0 |
| T..M | 1.24 | 277 | 1.0 |
| EC.I | 1.23 | 38 | 0.0 |
| EVT | 1.22 | 78 | 0.0 |
| Q..VT | 1.22 | 91 | 0.0 |
| G…I | 1.22 | 400 | 3.0 |
| P…C | 1.22 | 341 | 1.0 |
| T.V | 1.21 | 388 | 3.0 |
| Q.G | 1.21 | 430 | 5.0 |
| PI | 1.21 | 395 | 2.0 |
| I..P | 1.21 | 380 | 3.0 |
| MT | 1.20 | 288 | 1.0 |
| PPA | 1.20 | 172 | 0.0 |
| H…S | 1.19 | 368 | 2.0 |
| VP | 1.19 | 445 | 6.0 |
| S.F..C | 1.18 | 63 | 0.0 |
| ET | 1.18 | 399 | 4.0 |
| Q..SS | 1.18 | 166 | 0.0 |
| CQL | 1.17 | 76 | 0.0 |
| K…P | 1.17 | 412 | 3.0 |
| PG.L | 1.17 | 217 | 0.0 |
| IK..D | 1.17 | 55 | 0.0 |
| E.KV | 1.17 | 68 | 0.0 |
| I.D.T | 1.16 | 36 | 0.0 |
| E…F..E | 1.15 | 82 | 0.0 |
| L.PP | 1.15 | 206 | 0.0 |
| IT | 1.15 | 327 | 1.0 |
| L..KE | 1.15 | 91 | 0.0 |
| K.D | 1.15 | 362 | 2.0 |
| K.T | 1.15 | 357 | 3.0 |
| AP | 1.14 | 433 | 8.5 |
| N…T | 1.13 | 318 | 2.0 |
| P..M | 1.13 | 338 | 2.0 |
| VA..K | 1.13 | 103 | 0.0 |
| S…P | 1.13 | 481 | 9.0 |
| E..P | 1.12 | 420 | 5.0 |
| I…D..D | 1.12 | 64 | 0.0 |
| K…T | 1.11 | 348 | 2.0 |
| PG..A | 1.10 | 201 | 0.0 |
| T..I | 1.10 | 342 | 1.0 |
| T…K | 1.09 | 370 | 2.0 |
| L.G.C | 1.08 | 86 | 0.0 |
| E…T | 1.08 | 362 | 4.0 |
| R.T | 1.07 | 399 | 3.0 |
| E..R..E | 1.06 | 113 | 0.0 |
| C.LN | 1.06 | 43 | 0.0 |
| T.F | 1.06 | 303 | 1.0 |
| R.P | 1.06 | 423 | 6.5 |
| FT.T | 1.06 | 74 | 0.0 |
| GL | 1.05 | 424 | 9.0 |
| P.L | 1.05 | 425 | 12.0 |
| T.K | 1.04 | 383 | 2.0 |
| V..K | 1.04 | 380 | 2.0 |
| E..EI | 1.03 | 102 | 0.0 |
| T.I | 1.02 | 352 | 1.0 |
| G..L.G | 1.02 | 226 | 0.0 |
| P…R | 1.02 | 428 | 4.0 |
| F.G | 1.02 | 398 | 3.0 |
| Q..S | 1.02 | 429 | 6.0 |
| G..E | 1.01 | 393 | 4.0 |
| T.Y | 1.01 | 300 | 1.0 |
| R.G | 1.01 | 404 | 4.0 |
| T.VV | 1.01 | 69 | 0.0 |
| P..F | 1.01 | 349 | 4.0 |
| YT | 1.00 | 313 | 1.0 |
| K.E | 1.00 | 381 | 2.0 |
| L..P | 0.98 | 428 | 7.0 |
| E.S | 0.98 | 431 | 5.0 |
| E.Q | 0.98 | 380 | 3.0 |
| D…T | 0.98 | 364 | 2.0 |
| VC | 0.98 | 315 | 1.0 |
| M…T | 0.97 | 312 | 1.0 |
| E..S | 0.97 | 419 | 6.0 |
| TH | 0.97 | 313 | 1.0 |
| V…K | 0.97 | 309 | 1.0 |
| L.TS | 0.96 | 185 | 0.0 |
| H…G | 0.96 | 355 | 2.0 |
| V..D | 0.96 | 371 | 2.0 |
| KAK | 0.96 | 65 | 0.0 |
| EIS | 0.96 | 81 | 0.0 |
| V.E | 0.96 | 386 | 3.0 |
| R.S | 0.95 | 442 | 5.0 |
| E..L…E | 0.93 | 105 | 0.0 |
| QAS | 0.93 | 168 | 0.0 |
| G..SL | 0.93 | 226 | 0.0 |
| E..E | 0.93 | 373 | 4.0 |
| E.P | 0.92 | 400 | 4.0 |
| EL.P | 0.92 | 155 | 0.0 |
| I…K | 0.91 | 375 | 1.0 |
| S..M | 0.91 | 300 | 1.0 |
| TL | 0.90 | 390 | 3.5 |
| Y.E | 0.90 | 255 | 1.0 |
| V…C | 0.89 | 296 | 1.0 |
| QE | 0.89 | 397 | 4.0 |
| V…E | 0.88 | 346 | 1.0 |
| R.E | 0.88 | 353 | 3.0 |
| H.C | 0.87 | 132 | 0.0 |
| K…D | 0.87 | 331 | 1.0 |
| Y.T | 0.86 | 261 | 1.0 |
| R.D | 0.85 | 327 | 1.0 |
| N.T | 0.85 | 355 | 1.0 |
| S.F…S | 0.84 | 106 | 0.0 |
| I..K | 0.83 | 316 | 1.0 |
| E.K | 0.82 | 273 | 1.0 |
| L…N | 0.82 | 378 | 2.0 |
| L..AK | 0.82 | 117 | 0.0 |
| LQ.Q | 0.82 | 96 | 0.0 |
| V..S | 0.82 | 403 | 4.0 |
| VD | 0.81 | 348 | 1.0 |
| K.S | 0.81 | 388 | 3.0 |
| V..E | 0.81 | 414 | 2.0 |
| E…K | 0.81 | 385 | 1.0 |
| KCE | 0.81 | 57 | 0.0 |
| R…E | 0.80 | 303 | 2.0 |
| E.E | 0.79 | 314 | 2.0 |
| C..L | 0.79 | 278 | 1.0 |
| LT..E | 0.78 | 103 | 0.0 |
| A.D | 0.78 | 357 | 2.0 |
| S..E | 0.77 | 419 | 6.0 |
| QQ.L | 0.77 | 95 | 0.0 |
| VE | 0.76 | 348 | 1.0 |
| YE | 0.76 | 255 | 1.0 |
| I…E | 0.74 | 294 | 1.0 |
| V..Q | 0.73 | 379 | 2.0 |
| I…Q | 0.73 | 315 | 1.0 |
| Q.R | 0.72 | 369 | 1.0 |
| S.L | 0.72 | 417 | 7.0 |
| L..E | 0.72 | 414 | 3.0 |
| E…H | 0.70 | 282 | 1.0 |
| LE.R | 0.70 | 107 | 0.0 |
| K…I | 0.69 | 293 | 1.0 |
| Q.K | 0.68 | 346 | 1.0 |
| F.T | 0.67 | 305 | 1.0 |
| F..G | 0.66 | 359 | 2.0 |
| LE | 0.66 | 375 | 2.0 |
| EL | 0.61 | 373 | 3.0 |
| E..M | 0.59 | 227 | 0.0 |
| K…L | 0.59 | 381 | 1.0 |
| E.L | 0.59 | 376 | 2.0 |
| L…Q | 0.56 | 384 | 3.0 |
| QL | 0.55 | 330 | 2.0 |

The data in the table above can be visualized as wordclouds. To avoid plotting problems, any infinitely enriched motifs were first removed from the data. In this analyses we are not interested in the peculiarities of particular proteins, but in the properties of this group of proteins as a whole, so any motifs found in less than 5% of the proteins or in only one protein, were also removed before plotting.

In the wordcoulds the height of the letters relates to either the number of proteins in the set of interest containing a given motif (left), the enrichment of a motif relative to the background proteome (middle), or the product of the scaled values of these two measures (right). In some datasets with very few motifs, the scaled values are numerically undefined and no wordcloud will be plotted.

In general there tends to be a negative correlation between the enrichment of a motif and the number of proteins in which it is found. It is possible that motifs that deviate from this trend (ie they are unusually enriched given the number of proteins in which they are found, or are in an unusually large number of proteins given their enrichment) might have particular biological significance. The scatter plots below plot the enrichment of the motifs against the number of proteins in which they are found (and vice versa) to help you visualize any deviations from the expected negative correlation. If a linear regression is appropriate, a regression line (either linear or polynomial) is shown in red and the shaded area represents the 95% confidence interval of the regression. No regression line is shown in cases with strong non-linearity. Points are labelled only if there is sufficient space in the plot.

You may be interested in grouping or subdividing your PSOI based on the motif content of the proteins. The heatmaps below provide a starting point for such approaches. The top heatmap displays a hierarchical clustering of the proteins based on the number of motifs they contain, and simultaneously a clustering of motifs based on their distributions amongst proteins. In contrast to the above analyses, the filters that remove the infinitely enriched motifs and motifs found in less than 5% of proteins are not applied. For clarity however, only the 30 motifs found most frequently amongst the proteins are used in the clustering for the first heatmap. Proteins containing none of the motifs are not displayed.

In the second heatmap, proteins are clustered based on motif enrichment in a given protein sequence with respect the the background sequences, and motifs are clustered based on their enrichment amongst proteins. The filter that removes motifs found in less than 5% of proteins is not applied, but infinitely enriched motifs are excluded. However clustering is restricted to the 30 motifs with the greatest overall enrichment in the dataset. Proteins containing none of the motifs are not displayed.

In the third heatmap, proteins are clustered based on motif enrichment in a given protein sequence with respect the the background sequences, and motifs are clustered based on their enrichment amongst proteins. The difference to the second heatmap is that the filter that requires a motif to be in 5% of proteins (and more than one protein) is applied. Clustering subseqeunctly restricted to the 30 motifs with the greatest overall enrichment in the dataset. Proteins containing none of the motifs are not displayed.
