# Supplementary material for: ProminTools: shedding light on proteins of unknown function in biomineralization with user friendly tools illustrated using mollusc shell matrix protein sequences
Source: PeerJ. 2020 Sep 11;8:e9852. doi: 10.7717/peerj.9852 (PMC7489238; doi:10.7717/peerj.9852)
Supplement: Supplemental Information 10 [file peerj-08-9852-s010.zip › cluster3/clus6fig.nb.html]

Cluster 6 figure


Code 

- Show All Code
- Hide All Code
- Download Rmd

# Cluster 6 figure


```
library(wordcloud)
```


```
Loading required package: RColorBrewer
```


```
library(dplyr)
```


```
Attaching package: ‘dplyr’

The following objects are masked from ‘package:stats’:

    filter, lag

The following objects are masked from ‘package:base’:

    intersect, setdiff, setequal, union
```


```
library(ggplot2)
library(ggrepel)
library(gplots)
```


```
Attaching package: ‘gplots’

The following object is masked from ‘package:wordcloud’:

    textplot

The following object is masked from ‘package:stats’:

    lowess
```


```
library(knitr)
library(kableExtra)
```


```
Attaching package: ‘kableExtra’

The following object is masked from ‘package:dplyr’:

    group_rows
```


```
library(ComplexHeatmap)
```


```
Loading required package: grid
========================================
ComplexHeatmap version 2.5.4
Bioconductor page: http://bioconductor.org/packages/ComplexHeatmap/
Github page: https://github.com/jokergoo/ComplexHeatmap
Documentation: http://jokergoo.github.io/ComplexHeatmap-reference

If you use it in published research, please cite:
Gu, Z. Complex heatmaps reveal patterns and correlations in multidimensional 
  genomic data. Bioinformatics 2016.

This message can be suppressed by:
  suppressPackageStartupMessages(library(ComplexHeatmap))
========================================
```


```
library(matrixStats)
```


```
Attaching package: ‘matrixStats’

The following object is masked from ‘package:dplyr’:

    count
```


```
library(energy)
library(tibble)
library(circlize)
```


```
========================================
circlize version 0.4.10
CRAN page: https://cran.r-project.org/package=circlize
Github page: https://github.com/jokergoo/circlize
Documentation: https://jokergoo.github.io/circlize_book/book/

If you use it in published research, please cite:
Gu, Z. circlize implements and enhances circular visualization
  in R. Bioinformatics 2014.

This message can be suppressed by:
  suppressPackageStartupMessages(library(circlize))
========================================
```


```
scat1<-ggplot(mots_finite, aes(enrichment, positiveprots))
  scat1<-scat1 + geom_point() + theme_classic() + geom_text_repel(aes(label = mots_finite$motif), size = 3.5) +
    labs(x="Fold motif enrichment", y = "Number of proteins containing motif") +
    theme(axis.title = element_text(size = 12), axis.text=element_text(size = 11)) +
    scale_x_continuous(expand = c(0, 0), limits(0, 320)) + 
    scale_y_continuous(expand = c(0, 0), limits=c(0,5.2))
```


```
Error in limits(0, 320) : could not find function "limits"
```


```
svg(file = "/home/alastair/Documents/MPI/cyverse_tools/Lg_analyses/motifs_clusters_June/clus6mots/clus6scatter.svg", width = 7, height = 7)
scat1
dev.off()
```


```
null device 
          1
```


LS0tCnRpdGxlOiAiQ2x1c3RlciA2ICBmaWd1cmUiCm91dHB1dDogaHRtbF9ub3RlYm9vawotLS0KCmBgYHtyfQpsaWJyYXJ5KHdvcmRjbG91ZCkKbGlicmFyeShkcGx5cikKbGlicmFyeShnZ3Bsb3QyKQpsaWJyYXJ5KGdncmVwZWwpCmxpYnJhcnkoZ3Bsb3RzKQpsaWJyYXJ5KGtuaXRyKQpsaWJyYXJ5KGthYmxlRXh0cmEpCmxpYnJhcnkoQ29tcGxleEhlYXRtYXApCmxpYnJhcnkobWF0cml4U3RhdHMpCmxpYnJhcnkoZW5lcmd5KQpsaWJyYXJ5KHRpYmJsZSkKbGlicmFyeShjaXJjbGl6ZSkKYGBgCgpgYGB7cn0KbW90cy5wYXRoID0gIi9ob21lL2FsYXN0YWlyL0RvY3VtZW50cy9NUEkvY3l2ZXJzZV90b29scy9MZ19hbmFseXNlcy9tb3RpZnNfY2x1c3RlcnNfSnVuZS9jbHVzNm1vdHMvY2x1czZfbW90aWZzdW1tYXJ5LnR4dCIKbW90c19udW0ucGF0aCAgPSAiL2hvbWUvYWxhc3RhaXIvRG9jdW1lbnRzL01QSS9jeXZlcnNlX3Rvb2xzL0xnX2FuYWx5c2VzL21vdGlmc19jbHVzdGVyc19KdW5lL2NsdXM2bW90cy9jbHVzNl9mZ21vdGlmcy50eHQiCmZnX2VucmljaC5wYXRoID0gIi9ob21lL2FsYXN0YWlyL0RvY3VtZW50cy9NUEkvY3l2ZXJzZV90b29scy9MZ19hbmFseXNlcy9tb3RpZnNfY2x1c3RlcnNfSnVuZS9jbHVzNm1vdHMvY2x1czZfZmdlbnJpY2gudHh0IgpiaWFzLnBhdGggPSAiL2hvbWUvYWxhc3RhaXIvRG9jdW1lbnRzL01QSS9jeXZlcnNlX3Rvb2xzL0xnX2FuYWx5c2VzL21vdGlmc19jbHVzdGVyc19KdW5lL2NsdXM2bW90cy9jbHVzNl9wb3NpdGlvbnMudHh0IgoKZmxwc3B2YWwgPC0gMWUtMjAKCmRjb3JjbyA8LSAwLjY1CgoKCm1vdHM8LXJlYWQudGFibGUobW90cy5wYXRoLCBzZXA9Ilx0IiwgaGVhZGVyPVRSVUUpCm1vdHM8LW1vdHNbLDE6NF0KbW90X251bTwtcmVhZC50YWJsZShtb3RzX251bS5wYXRoLCBzZXA9Ilx0IiwgaGVhZGVyPVRSVUUsIHJvdy5uYW1lcz0xKQpmZ19lbnJpY2g8LXJlYWQudGFibGUoZmdfZW5yaWNoLnBhdGgsIHNlcD0iXHQiLCBoZWFkZXI9VFJVRSwgcm93Lm5hbWVzPTEpCgptb3RfbnVtID0gbW90X251bVsgcm93U3Vtcyhtb3RfbnVtKSE9MCwgXQpwcm90Y291bnQ8LW5yb3cobW90X251bSkKCm1vdHM8LW1vdHNbb3JkZXIobW90cyRlbnJpY2htZW50LCBkZWNyZWFzaW5nID0gVFJVRSksIF0KbW90cyRlbnJpY2htZW50PC1yb3VuZChtb3RzJGVucmljaG1lbnQsIDIpCgptb3RzX2Zpbml0ZTwtc3Vic2V0KG1vdHMsIGVucmljaG1lbnQhPSJJbmYiKQpwcm90c181cGM8LTAuMDUqcHJvdGNvdW50Cm1vdHNfZmluaXRlPC1zdWJzZXQobW90c19maW5pdGUsIHBvc2l0aXZlcHJvdHMgPj0gcHJvdHNfNXBjICYgcG9zaXRpdmVwcm90cyA+IDEpCgptb3RzX2Zpbml0ZSRlbnJpY2htZW50MjwtbW90c19maW5pdGUkZW5yaWNobWVudAptb3RzX2Zpbml0ZSRlbnJpY2htZW50Mlttb3RzX2Zpbml0ZSRlbnJpY2htZW50MiA9PSAnSW5mJ108LTEwMDAwMDAKbW90c19maW5pdGUkZW5yaWNobWVudDI8LWFzLm51bWVyaWMobW90c19maW5pdGUkZW5yaWNobWVudDIpCm1vdHNfZmluaXRlJGVucmljaG1lbnRfc2NhPC0gKG1vdHNfZmluaXRlJGVucmljaG1lbnQyIC0gbWluKG1vdHNfZmluaXRlJGVucmljaG1lbnQyKSkgLyAobWF4KG1vdHNfZmluaXRlJGVucmljaG1lbnQyKSAtIG1pbihtb3RzX2Zpbml0ZSRlbnJpY2htZW50MikpCm1vdHNfZmluaXRlJHBvc2l0aXZlcHJvdHNfc2NhPC0gKG1vdHNfZmluaXRlJHBvc2l0aXZlcHJvdHMgLSBtaW4obW90c19maW5pdGUkcG9zaXRpdmVwcm90cykpIC8gKG1heChtb3RzX2Zpbml0ZSRwb3NpdGl2ZXByb3RzKSAtIG1pbihtb3RzX2Zpbml0ZSRwb3NpdGl2ZXByb3RzKSkKCm1vdHNfZmluaXRlJHNjYWxwcm9kPC0gKDYgKiBtb3RzX2Zpbml0ZSRlbnJpY2htZW50X3NjYSArIDEpICogKDYgKiBtb3RzX2Zpbml0ZSRwb3NpdGl2ZXByb3RzX3NjYSArIDEpCgpzdmcoZmlsZSA9ICIvaG9tZS9hbGFzdGFpci9Eb2N1bWVudHMvTVBJL2N5dmVyc2VfdG9vbHMvTGdfYW5hbHlzZXMvbW90aWZzX2NsdXN0ZXJzX0p1bmUvY2x1czZtb3RzL2NsdXM2V0Muc3ZnIikKCWxheW91dChtYXRyaXgoYygxLDIsMyw0LDUsNiksIG5yb3c9MiwgYnlyb3cgPSBUUlVFKSwgaGVpZ2h0cz1jKDAuNywgMS41KSkKCXBhcihtYXI9cmVwKDAsIDQpKQoJcGxvdC5uZXcoKQoJdGV4dCh4PTAuNSwgeT0wLjIsICJOdW1iZXIgb2YgcHJvdGVpbnNcbmNvbnRhaW5pbmcgbW90aWYiLCBjZXg9MS41LCBwb3M9MykKCXBsb3QubmV3KCkKCXRleHQoeD0wLjUsIHk9MC4yLCAiRW5yaWNobWVudCIsIGNleD0xLjUsIHBvcz0zKQoKCXBsb3QubmV3KCkKCXRleHQoeD0wLjUsIHk9MC4yLCAiUHJvZHVjdCBvZiBzY2FsZWRcbnZhbHVlcyIsIGNleD0xLjUsIHBvcz0zKQoJd29yZGNsb3VkKG1vdHNfZmluaXRlJG1vdGlmLCBtb3RzX2Zpbml0ZSRwb3NpdGl2ZXByb3RzLCBzY2FsZT1jKDMsIDAuMiksIHJhbmRvbS5vcmRlcj1GQUxTRSwgbWluLmZyZXEgPSAxLCByb3QucGVyPSIwIiwgY29sb3JzPSAoYnJld2VyLnBhbCg5LCAiWWxPclJkIikpKQoKCXdvcmRjbG91ZChtb3RzX2Zpbml0ZSRtb3RpZiwgbW90c19maW5pdGUkZW5yaWNobWVudCwgc2NhbGU9YygzLCAwLjIpLCBtaW4uZnJlcSA9IDEsIHJhbmRvbS5vcmRlcj1GQUxTRSwgcm90LnBlcj0iMCIsIGNvbG9ycz0gKGJyZXdlci5wYWwoOSwgIllsT3JSZCIpKSkKCgl3b3JkY2xvdWQobW90c19maW5pdGUkbW90aWYsIG1vdHNfZmluaXRlJHNjYWxwcm9kLCBzY2FsZT1jKDMsIDAuMiksIHJhbmRvbS5vcmRlcj1GQUxTRSwgbWluLmZyZXEgPSAxLCByb3QucGVyPSIwIiwgY29sb3JzPSAoYnJld2VyLnBhbCg5LCAiWWxPclJkIikpLCBtYWluPSJwcm9kdWN0IikKCglkZXYub2ZmKCkKCnNjYXQxPC1nZ3Bsb3QobW90c19maW5pdGUsIGFlcyhlbnJpY2htZW50LCBwb3NpdGl2ZXByb3RzKSkKICBzY2F0MTwtc2NhdDEgKyBnZW9tX3BvaW50KCkgKyB0aGVtZV9jbGFzc2ljKCkgKyBnZW9tX3RleHRfcmVwZWwoYWVzKGxhYmVsID0gbW90c19maW5pdGUkbW90aWYpLCBzaXplID0gMy41KSArCiAgICBsYWJzKHg9IkZvbGQgbW90aWYgZW5yaWNobWVudCIsIHkgPSAiTnVtYmVyIG9mIHByb3RlaW5zIGNvbnRhaW5pbmcgbW90aWYiKSArCiAgICB0aGVtZShheGlzLnRpdGxlID0gZWxlbWVudF90ZXh0KHNpemUgPSAxMiksIGF4aXMudGV4dD1lbGVtZW50X3RleHQoc2l6ZSA9IDExKSkgKwogICAgc2NhbGVfeF9jb250aW51b3VzKGV4cGFuZCA9IGMoMCwgMCksIGxpbWl0cz1jKDAsIDMyMCkpICsgCiAgICBzY2FsZV95X2NvbnRpbnVvdXMoZXhwYW5kID0gYygwLCAwKSwgbGltaXRzPWMoMCwgNC44KSkKYGBgCgpgYGB7cn0KCnN2ZyhmaWxlID0gIi9ob21lL2FsYXN0YWlyL0RvY3VtZW50cy9NUEkvY3l2ZXJzZV90b29scy9MZ19hbmFseXNlcy9tb3RpZnNfY2x1c3RlcnNfSnVuZS9jbHVzNm1vdHMvY2x1czZzY2F0dGVyLnN2ZyIsIHdpZHRoID0gNywgaGVpZ2h0ID0gNykKc2NhdDEKZGV2Lm9mZigpCmBgYAoKCg==
