# Supplementary material for: ProminTools: shedding light on proteins of unknown function in biomineralization with user friendly tools illustrated using mollusc shell matrix protein sequences
Source: PeerJ. 2020 Sep 11;8:e9852. doi: 10.7717/peerj.9852 (PMC7489238; doi:10.7717/peerj.9852)
Supplement: Supplemental Information 10 [file peerj-08-9852-s010.zip › cluster6/ZTEST.html]

ZTEST.R


# ZTEST.R

#### root

#### 2020-07-03

#’ — title: Overrepresented motifs in your proteins of interest author: A tool by Alastair Skeffington hosted by Cyverse and making use of the motif-x motif finding engine —

Here we go

```
x<-TRUE

if(x){print("ONE Eval is true, in print statement\nAfter new line.")}
```

```
## [1] "ONE Eval is true, in print statement\nAfter new line."
```

Blablabla

```
print("Eval is true, in print statement\nAfter new line.")
```

```
## [1] "Eval is true, in print statement\nAfter new line."
```

```
y<-FALSE
```

```
if(y){print("This shouldn't be printed.")}else{print("Whne main block doesn't run")}
```

Bye
